# Macrocyclic Peptidomimetic Plasmepsin X Inhibitors with Potent *In Vitro* and *In Vivo* Antimalarial Activity

**DOI:** 10.1021/acs.jmedchem.3c00812

**Published:** 2023-07-28

**Authors:** Vadims Kovada, Chrislaine Withers-Martinez, Raitis Bobrovs, Hele̅na Ce̅rule, Edgars Liepins, Solveiga Grinberga, Fiona Hackett, Christine R. Collins, Agrita Kreicberga, María Belén Jiménez-Díaz, Iñigo Angulo-Barturen, Dace Rasina, Edgars Suna, Kristaps Jaudzems, Michael J. Blackman, Aigars Jirgensons

**Affiliations:** † Latvian Institute of Organic Synthesis, Riga LV-1006, Latvia; ‡ Malaria Biochemistry Laboratory, The Francis Crick Institute, London NW1 1AT, United Kingdom; § The Art of Discovery SL, Biscay Science and Technology Park, Derio, 48160 Bizkaia, Basque Country, Spain; ∥ Faculty of Infectious and Tropical Diseases, London School of Hygiene & Tropical Medicine, London WC1E 7HT, United Kingdom

## Abstract

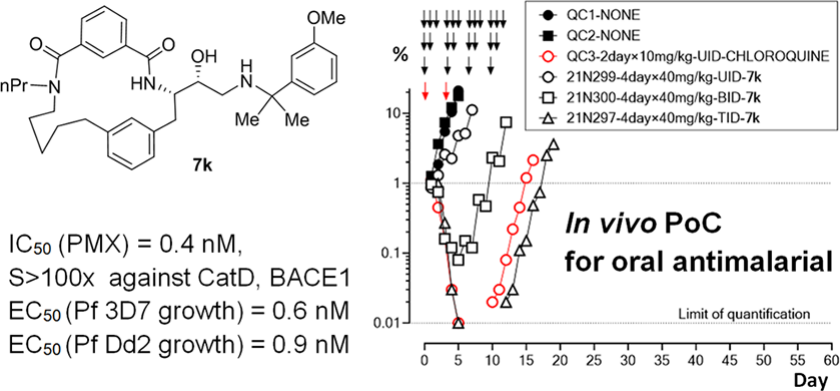

The *Plasmodium falciparum* aspartic protease plasmepsin X (PMX) is essential for the egress of invasive merozoite forms of the parasite. PMX has therefore emerged as a new potential antimalarial target. Building on peptidic amino alcohols originating from a phenotypic screening hit, we have here developed a series of macrocyclic analogues as PMX inhibitors. Incorporation of an extended linker between the S1 phenyl group and S3 amide led to a lead compound that displayed a 10-fold improved PMX inhibitory potency and a 3-fold improved half-life in microsomal stability assays compared to the acyclic analogue. The lead compound was also the most potent of the new macrocyclic compounds in *in vitro* parasite growth inhibition. Inhibitor **7k** cleared blood-stage *P. falciparum* in a dose-dependent manner when administered orally to infected humanized mice. Consequently, lead compound **7k** represents a promising orally bioavailable molecule for further development as a PMX-targeting antimalarial drug.

## 1 Introduction

The mosquito-borne disease malaria impacts half of the world′s population in tropical and subtropical areas causing ∼600 thousand deaths annually, with infants and pregnant women representing particularly high-risk groups.^1^ Malaria is also a significant cause of poverty in endemic countries.^2^ Malaria morbidity and mortality has been significantly reduced over recent decades through a combination of approaches, including vector control, insecticide-impregnated bed nets, and chemoprophylactic and chemotherapeutic means.^3^ In its 2015 Global Technical Strategy, the World Health Organization set a target of 90% reduction in clinical cases and deaths due to malaria by 2030. However, efforts to reach this target are challenged by the spread of drug-resistant *Plasmodium* parasites, the causative agent of malaria. *Plasmodium* strains resistant to nearly all clinically used drugs, including artemisinins, the current front-line antimalarial drug class,^4−6^ have been reported. New drugs for the treatment of acute uncomplicated malaria (Medicines for Malaria Target Product Profile-1, TPP-1) and chemoprotection (TPP-2) are therefore urgently needed to replenish the arsenal of antimalarial drugs.^7^ While recently approved antimalarial drug products have been dominated by combination therapies based on existing drugs,^8^ a current focus is on the discovery of new drugs that act through novel modes of action.

**Figure 1:**
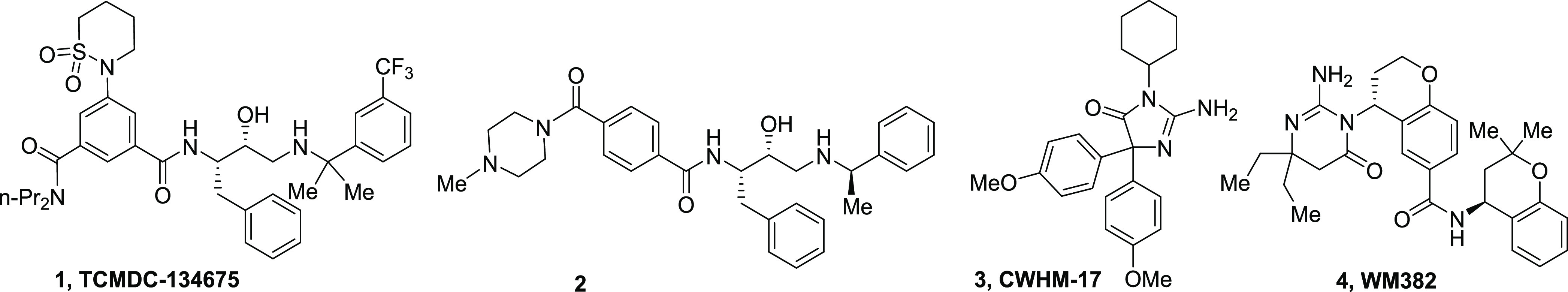
Representative PMIX and/or PMX inhibitors.

*Plasmodium* aspartic proteases, termed plasmepsins (PMs), have been explored as potential antimalarial drug targets for nearly three decades,^9−12^ but to date, no PM inhibitor has advanced to the clinic. Ten PM isoforms are encoded in the genome of *Plasmodium falciparum* (Pf), the deadliest species affecting humans. Seven *P. falciparum* PMs (I–V, IX, and X) are expressed during the asexual blood stages of the parasite lifecycle that are responsible for all clinical manifestations of the disease, so these enzymes have been considered as potential drug targets. Extensive work on PMs I–IV, which play roles in the proteolytic breakdown of hemoglobin in the parasite digestive vacuole, has shown this group of enzymes to be highly redundant, rendering them unfit as drug targets.^13^ More recent efforts have focused on the non-degradative PMs, all of which appear essential for parasite viability. PMV is expressed in the parasite endoplasmic reticulum, where it functions to modify a subclass of parasite proteins that are exported into the host red blood cell (RBC).^14^ PMIX and PMX are maturases essential for the egress (escape) of invasive forms of the parasite, called merozoites, from the host RBC and for merozoite invasion of fresh RBCs.^15,16^ Several amino alcohol-based peptidomimetic inhibitors of PMIX and/or PMX have been described that potently inhibit parasite proliferation *in vitro*. Phenotypic screening hit **1** was the first identified compound of this type, proving to be a potent inhibitor of PMX, while another amino alcohol-based compound **2**, was shown to inhibit both PMIX and PMX (Figure 1).^15,16^ Aminohydantoins have also been developed as non-peptidic PMIX and PMX inhibitors. A potent PMX inhibitor, compound **3**, resulted from the optimization of an HTS hit and was further optimized to generate analogues with *in vivo* activity in murine malaria models.^17,18^ Aminohydantoin **4** was shown to be a dual PMIX and PMX inhibitor and also displayed high antimalarial potency *in vitro* and *in vivo*.^19^

**Figure 2:**
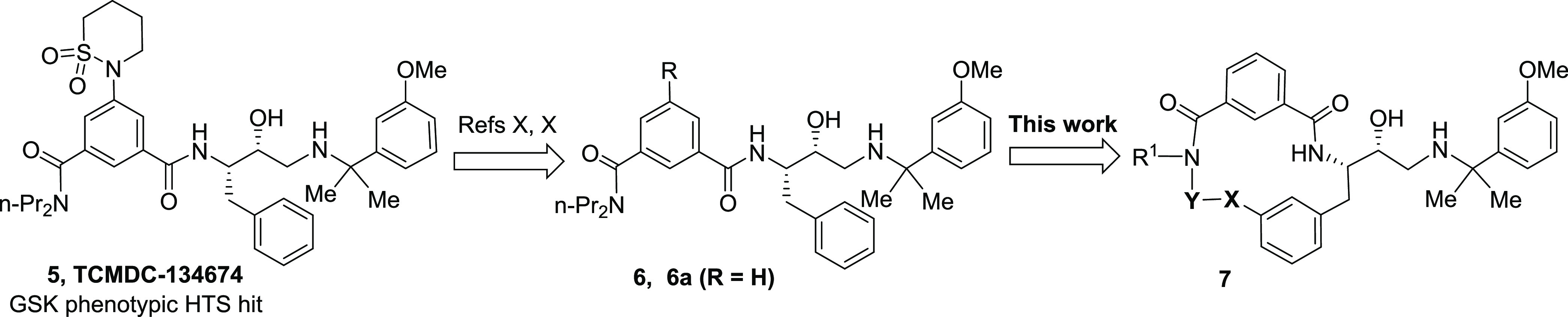
Development of phenotypic screening hit **5** to macrocyclic peptidic inhibitor series **7**.

Our efforts in PM inhibitor antimalarial drug discovery started with peptidomimetic hydroxyethylamine compound **5**, which was identified as a potent inhibitor of *P. falciparum* growth in a phenotypic HTS screen performed by GSK (Figure 2).^20^ Early analysis showed that hit **5** was a PM inhibitor, using PMI, PMII, and PMIV as accessible model proteins.^21^ Work aimed at determining crucial substructures for PM inhibitory potency and reducing inhibition of the human aspartic protease cathepsin D (CatD) resulted in analogue series **6**.^22^ The structurally simplest analogue from this series, compound **6a**, was one of the most potent inhibitors of parasite growth. Further examination showed that compound **6a** inhibits not only degradative PMs but also PMX, as indicated by the capacity of **6a** to inhibit maturation of the *P. falciparum* PMX substrate subtilisin-like protease-1 (SUB1), a key effector in merozoite egress.^23^ However, *in vitro* absorption, distribution, metabolism, and excretion (ADME) profiling of inhibitor **6a** (*vide infra*) revealed that it has low microsomal stability; thus, it was not reasonable to further investigate efficacy *in vivo*. Since macrocyclization of peptidic compounds can often improve ADME properties,^24−27^ we were prompted to use **6a** as a basis to design macrocyclic analogues **7**. Here, we describe the generation of potent, subnanomolar macrocyclic PMX inhibitors that hold potential for development as a new class of antimalarial drugs.

## 2 Results and Discussion

### 2.1 Synthesis of Macrocyclic PM Inhibitors

**Scheme 1:**
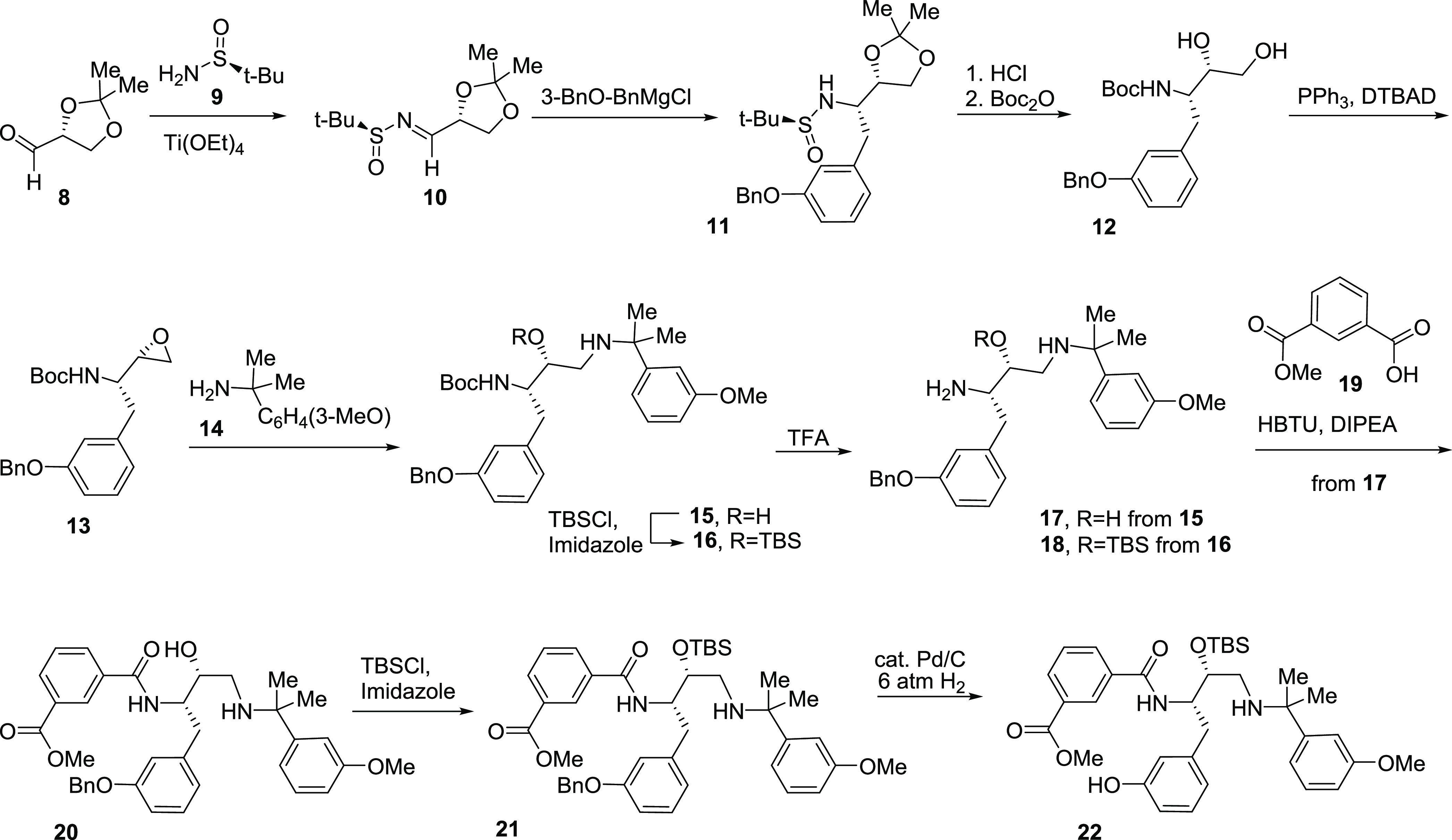
Synthesis of Building Blocks **16**, **18**, and **22** for the Preparation of Inhibitors **7a**–**k**

Principal building blocks **16**, **18**, and **22** for the synthesis of macrocyclic amides **7a**–**k** were prepared according to Scheme 1. Enantioenriched protected glyceraldehyde **8** was transformed into sulfinimine **10** in the reaction with (*S*)-*tert*-butanesulfinamide (**9**),^28^ which was subjected to the diastereoselective Grignard reaction to give sulfonamide **11**.^29,30^

Acetonide and sulfinyl groups in intermediate **11** were removed by treatment with acid, and amine was *N*-Boc protected to give amino alcohol derivative **12**. The Mitsunobu reaction provided aminoepoxide **13**, which was ring-opened with benzylamine derivative **14.** The resulting diaminoalcohol **15** was *O*-TBS protected to give an intermediate **16.** Both compounds **15** and **16** were subjected to Boc-deprotection to give amines **17** and **18**, respectively. Amine **17** was *N*-coupled with isophthalic acid monoester **19** to produce amide **20**. *O*-TBS protection provided intermediate **21**, which was *O*-debenzylated under hydrogenolysis conditions to provide the building block **22**.

**Scheme 2:**
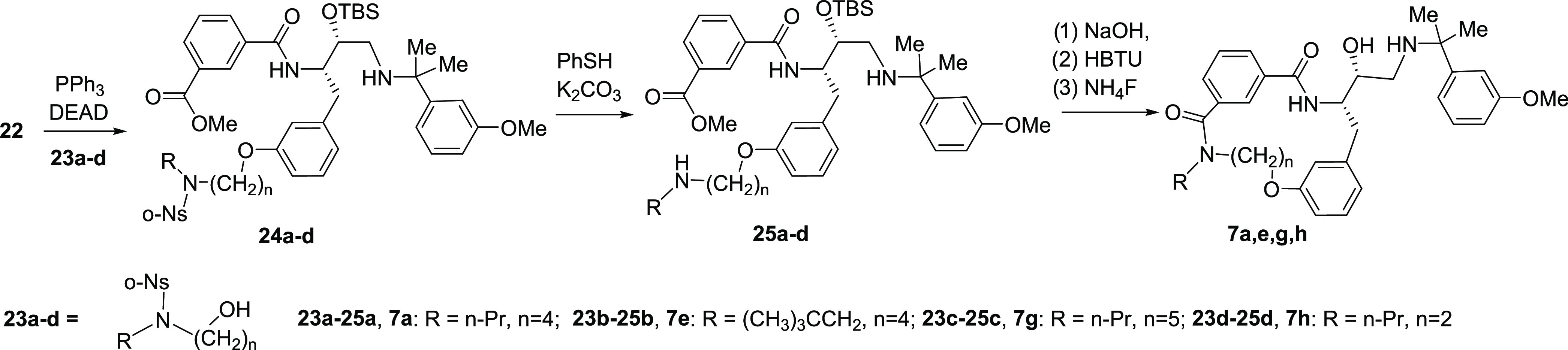
Synthesis of Inhibitors **7a,e,g,h**

Building block **22** was used to prepare the target compounds **7a,e,g,h** (Scheme 2). *O*-Alkylation with alcohols **23a**–**d** in Mitsunobu reaction conditions provided intermediates **24a**–**d**. These were subjected to cleavage of the *N*-nosyl group leading to amines **25a**–**d**. Hydrolysis of the ester group in intermediates **25a**–**d** followed by macrolactamization and removal of the *O*-TBS group resulted in target compounds **7a,e,g,h**.

**Scheme 3:**
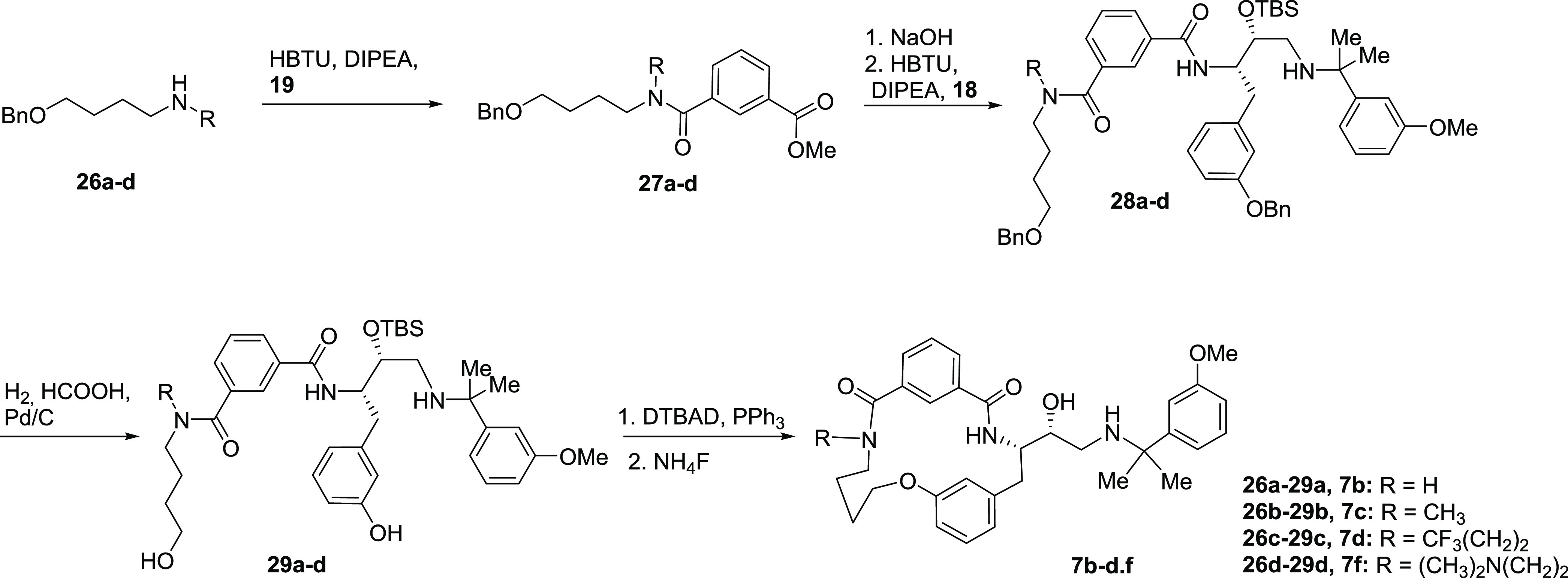
Synthesis of Inhibitors **7b**–**d,f**

Inhibitors **7b**–**d,f** were prepared starting from amines **26a**–**d** (Scheme 3). These were acylated with isophthalic acid monoester **19** to give isophthalic monoamides **27a**–**d.** Hydrolysis and subsequent coupling with the building block **18** (Scheme 1) provided intermediates **28a**–**d**. *O-*Benzyl deprotection in compounds **28a**–**d** provided alcohols **29a**–**d**, which were subjected to macrocyclization and *O*-desilylation, leading to target compounds **7b**–**d,f**.

**Scheme 4:**
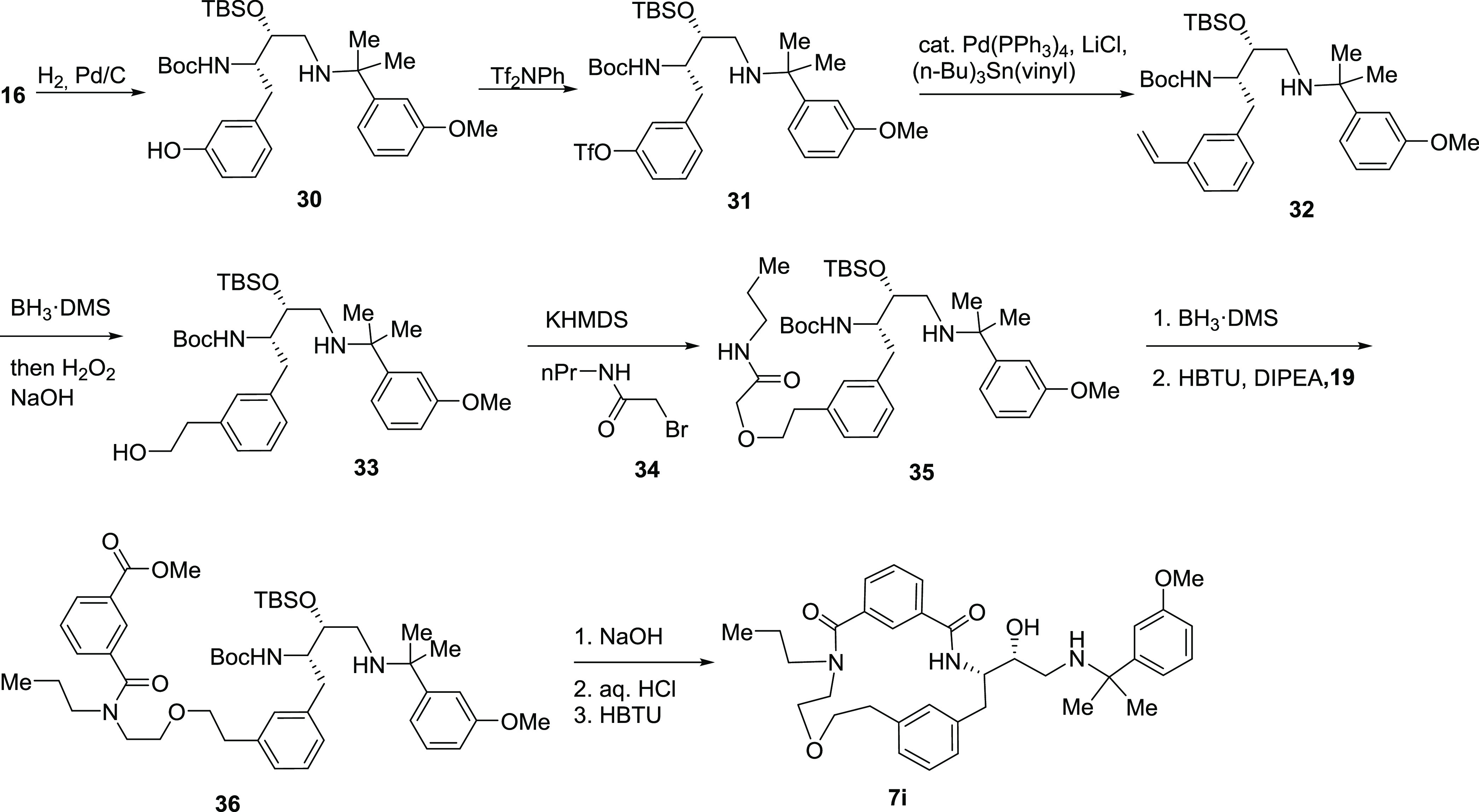
Synthesis of Inhibitor **7i**

Target compound **7i** was prepared from building block **16** (Scheme 4). This was *O*-debenzylated by hydrogenolysis to give an intermediate **30**. The phenolic hydroxyl group in compound **30** was triflated, and the triflate **31** was subjected to coupling with a vinyltin reagent to give intermediate **32**. Hydroboration–oxidation of the double bond in intermediate **32** provided alcohol **33**, which was *O*-alkylated with alfa-bromo amide **34**. The resulting amide **35** was reduced with borane to an intermediate amine, which was coupled to isophthalic acid monoester **19** to give amide **36**. Hydrolysis of the ester group and the cleavage of the *N*-Boc in intermediate **36** was followed by macrocyclization to give target compound **7i**.

**Scheme 5:**
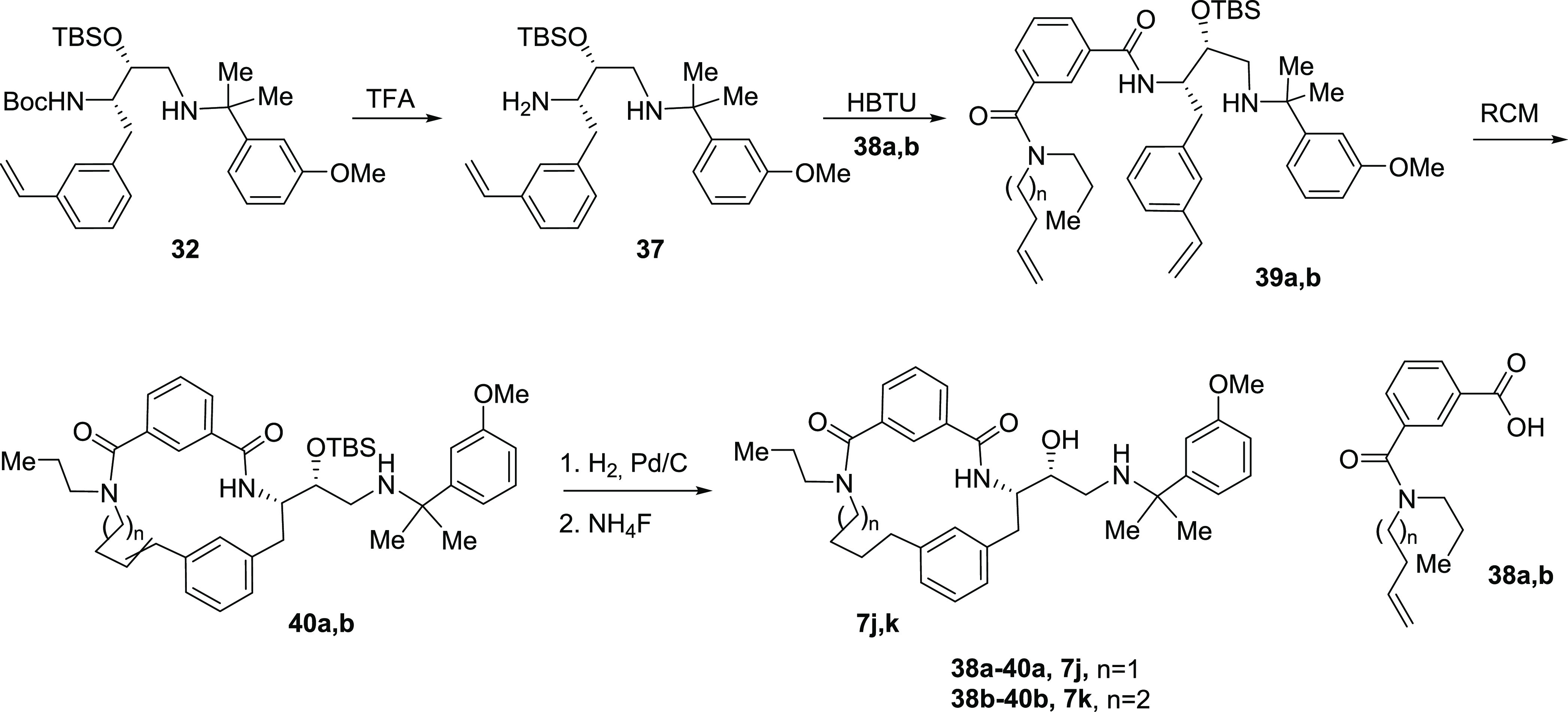
Synthesis of Inhibitors **7j,k**

Target compounds **7j,k** were prepared according to Scheme 5. Intermediate **32** was subjected to Boc-deprotection, and the resulting amine **37** was coupled to amines **38a,b**. Ring-closing metathesis of intermediates **39a,b** resulted in macrocyclic amides **40a,b**. Hydrogenation of a double bond and desilylation provided target compounds **7j,k**.

### 2.2 Plasmepsin Inhibitory Potency and Structure–Activity Relationship (SAR)

Macrocycles **7a**–**k** were tested for their ability to inhibit recombinant *P. falciparum* PMX as well as the hemoglobinase PMIV in order to determine selectivity for these enzymes and to examine the association between parasite growth-inhibitory activity and inhibition of the enzymatic activity of the PM isoforms. Human aspartic proteases CatD and BACE1 were used for selectivity counter screens (Tables 1 and 2). Acyclic inhibitor **6a** was previously shown to be a PMX inhibitor by its capacity to inhibit intracellular maturation of *P. falciparum* SUB1. Using recombinant PMX, the PMX inhibitory potency of compound **6a** was confirmed in enzymatic assays to be in the low nanomolar range (Table 1, entry 1). Macrocyclization *via* the phenolic oxygen and carboxamide resulted in inhibitor **7a** with very similar PMX and PMIV inhibitory potency but somewhat reduced selectivity against CatD. Exploration of substitution of the carboxamide nitrogen showed that hydrogen, methyl, and trifluoropropyl had little impact on PMX inhibitory potency, while PMIV inhibition was significantly reduced (compounds **7b**–**d**) (Table 1, entries 2–4). Compound **7e** bearing a more lipophilic pivaloyl substituent showed slightly improved PMX inhibitory potency and good selectivity against CatD and BACE1. Installation of a dimethylaminoethyl substituent as a solubilizing group at the carboxamide resulted in compound **7f** with 4-fold reduced PMX inhibitory potency compared to analogue **7a** but increased selectivity against PMIV, CatD, and BACE1.

**Table 1:**
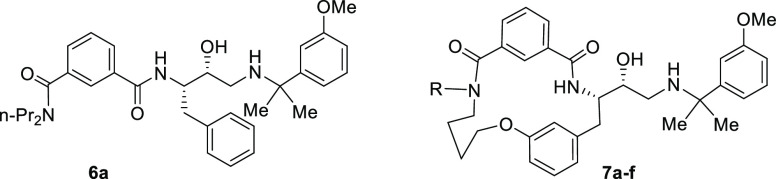
PMX, PMIV, CatD, and BACE1 Inhibition by Macrocycles **6a** and **7a**–**f**

#	cmpd.	R	PMX, nM	PMIV, nM	CatD, nM	BACE1, nM
1	**6a**	-	6.2 ± 0.3	58 ± 3^a^	1150 ± 50^a^	n.d.
2	**7a**	*n*-Pr	4.1 ± 0.3	60 ± 3	270 ± 20	380 ± 20
3	**7b**	H	11.2 ± 0.7	510 ± 30	650 ± 30	910 ± 40
4	**7c**	Me	5.3 ± 3.0	170 ± 20	320 ± 20	1030 ± 50
5	**7d**	CF_3_(CH_2_)_2_	8.6 ± 2.4	61 ± 4	230 ± 20	2100 ± 100
6	**7e**	(CH_3_)_3_CCH_2_	2.5 ± 0.2	40	630	1900 ± 100
7	**7f**	(CH_3_)_2_NCH_2_CH_2_	16.7 ± 1.5	1730 ± 90	6800 ± 340	52400 ± 1600

a Data from ref (22).

**Table 2:**
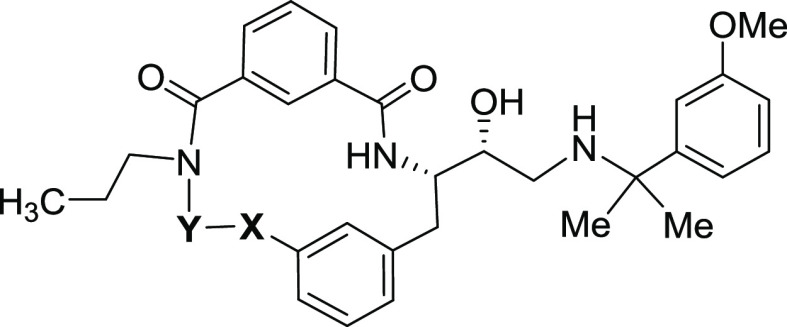
PMX, PMIV, CatD, and BACE1 Inhibition by Macrocycles **7g**–**h**

#	cmpd.	Y	X	PMX, nM	PMIV, nM	CatD, nM	BACE1, nM
1	**7g**	–(CH_2_)_5_–	O	6.9 ± 0.49	190	1200	6000 ± 300
2	**7h**	–(CH_2_)_2_–	O	6.8 ± 0.59	200	600	1850 ± 90
3	**7i**	–(CH_2_)_2_–O–CH_2_–	CH_2_	4.6 ± 0.07	81±4	1650±80	22 700 ± 1100
4	**7j**	–(CH_2_)_3_–	CH_2_	3.8 ± 0.69	74±4	1370±70	3020 ± 150
5	**7k**	–(CH_2_)_4_–	CH_2_	0.4 ± 0.03	17±2	330±20	2030 ± 100

Installation of a longer or a shorter linker between amide and phenyl groups (compounds **7g** and **7h**; Table 2, entries 1,2) did not significantly alter PMX inhibitory potency but reduced the capability to inhibit PMIV, CatD, and BACE1. Shifting an oxygen atom position in the linker (compound **7i**; Table 2, entry 3) or use of a shorter linker comprising only CH_2_ groups (compound **7j**; Table 2, entry 4) also maintained PMX and PMIV inhibitory potency similar to the parent compound, but reduced potency against CatD and BACE1.

Surprisingly an analogue of macrocycle **7a** containing only CH_2_ groups within the linker, compound **7k** (Table 2, entry 5), showed 10-fold improved PMX inhibitory potency and 3-fold improved PMIV inhibitory potency, while inhibition of CatD and BACE1 was similar to the parent compound **7a**. Collectively, these results showed that potent and selective macrocyclic PMX inhibitors could be derived through appropriate cyclization of compounds based on the linear peptidic hydroxyethylamine compounds **6** (Figure 2).

To gain insights into the structural features of **7a** and **7k** that might confer their differential PMX inhibitory potency, the crystal structure of *P. falciparum* PMX bound to the small inhibitor WM382 (compound **4**) (PDB: 7TBC) was used for *in silico* docking of **7a** and **7k** in ICM-Pro (Molsoft^31^).

**Figure 3:**
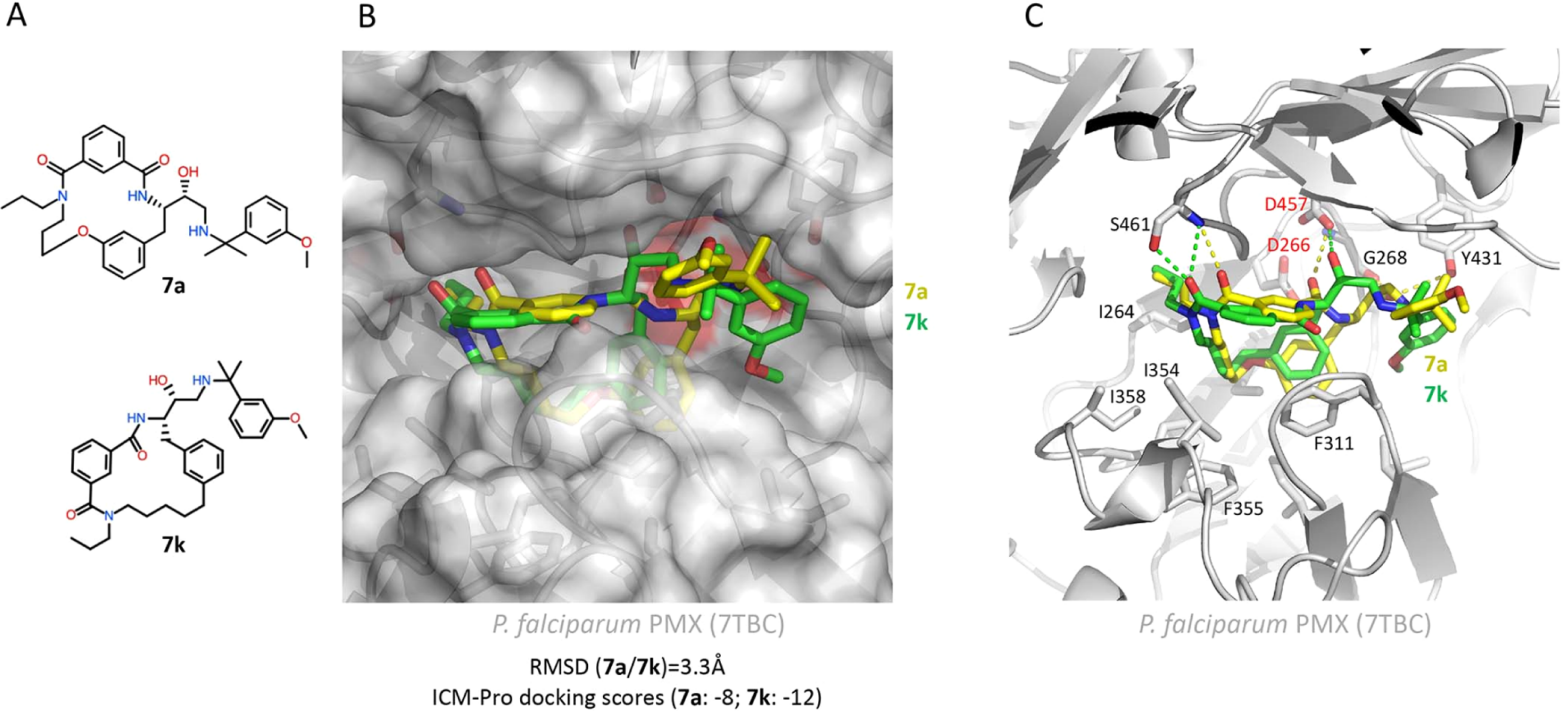
Macrocycles **7a** and **7k** docked in the PMX active site. (A) Chemical structures of **7a** and **7k**. (B) The molecular surface of PMX (PDB: 7TBC) shown as a semi-transparent envelope, illustrating the size and shape of the enzyme active site pocket containing both **7a** and **7k** docked inhibitors. The inhibitors are shown as sticks, yellow for **7a** (C: yellow, N: blue, O: red) and green for **7k** (C: green, N: blue, O: red). The ICM-pro docking scores (dimensionless) are −8 for **7a** and −12 for **7k**, with an RMSD value of 3.3 Å between the poses of the two inhibitors. (C) Vicinity of the PMX (PDB: 7TBC) active site in cartoon representation with the inhibitors and side chain residues shown as sticks, colored and labeled according to their properties. The catalytic aspartic acid dyad (Asp266 and Asp457) is labeled in red. Docked inhibitors are shown as sticks, **7a** in yellow (C: yellow, N: blue, O: red) and **7k** in green (C: green, N: blue, O: red). Hydrogen bonds are represented by dashed lines, yellow for **7a** and green for **7k**.

As a control for this operation, WM382 was first docked into PMX, providing an ICM-Pro score of −16 and a root-mean-square deviation (RMSD) value of 0.97 Å from the experimentally determined WM382 X-ray coordinates. Macrocycles **7a** and **7k** were found to dock in a similar manner in the active site of PMX (Figure 3) with a slightly better ICM-Pro score for **7k** (−12) as compared to **7a** (−8), consistent with the greater potency of **7k**. Predicted hydrogen bonds and hydrophobic interactions appeared to stabilize both inhibitors in the PMX active site, with each of the macrocycle hydroxyl extensions in the vicinity of Asp457, one of the two catalytic aspartyl residues.

### 2.3 Macrocyclic PM Inhibitors Display *P. falciparum* Growth-Inhibitory Activity *In Vitro*

The capacity of compounds **7a**–**k** to inhibit *P. falciparum* asexual blood-stage proliferation *in vitro* was assessed using two different *P. falciparum* strains: the chloroquine-sensitive strain 3D7 and the chloroquine-resistant strain Dd2. As shown in Table 3, all of the compounds **7a**–**k** displayed high growth-inhibitory potency against both *P. falciparum* strains. The correlation between *in vitro* parasite growth-inhibitory activity and PMX enzyme inhibitory activity was not perfect, suggesting some cell-permeability or stability issues. However, the most potent macrocyclic PMX inhibitor, **7k** (Table 2, entry 5), also displayed the most potent growth-inhibitory activity. Notably, there was a generally poor correlation between parasite growth-inhibitory activity and inhibition of the hemoglobin degradative enzyme PMIV. This is consistent with previous evidence that inhibition of PMIV cannot be exploited for antimalarial drug discovery due to significant redundancy in the hemoglobin degradation pathway.^13^ Based on these results, it was tentatively concluded that parasite growth inhibition was likely primarily through the inhibition of PMX.

**Table 3:** *P. falciparum* Growth Inhibition *In Vitro* and Association with PM Inhibition

#	cmpd	flow cytometry 3D7^a^ EC_50_ (nM)	flow cytometry Dd2^b^ EC_50_ (nM)	PMX, nM	PMIV, nM
1	**7a**	1.0 ± 0.3	4.7 ± 0.5	6.2 ± 0.3	58 ± 3^c^
2	**7b**	6.4 ± 0.9	13.1 ± 3.1	4.1 ± 0.3	60 ± 3
3	**7c**	2.6 ± 0.1	4.9 ± 0.9	11.2 ± 0.7	510 ± 30
4	**7d**	6.2 ± 1.0	9.5 ± 1.4	5.3 ± 3.0	170 ± 20
5	**7e**	2.2 ± 0.6	3.5 ± 0.4	8.6 ± 2.4	61 ± 4
6	**7f**	6.5 ± 1.2	9.6 ± 0.7	2.5 ± 0.2	40
7	**7g**	6.7 ± 2.3	9.6 ± 1.0	6.9 ± 0.49	190
8	**7h**	0.8 ± 0.4	6.7 ± 1.0	6.8 ± 0.59	200
9	**7i**	2.0 ± 0.3	2.0 ± 0.3	4.6 ± 0.07	81 ± 4
10	**7j**	2.7 ± 0.5	4.4 ± 0.5	3.8 ± 0.69	74 ± 4
11	**7k**	0.6 ± 0.1	0.9 ± 0.2	0.4 ± 0.03	17 ± 2

a 3D7: chloroquine-sensitive *P. falciparum* strain.

b Dd2: chloroquine-resistant *P. falciparum* strain.

c Data from ref (22).

### 2.4 *P. falciparum* Growth Inhibition by Macrocycles is through the Inhibition of PMX

To examine the mode of action of selected parasite growth-inhibitory macrocyclic inhibitors, highly synchronized *P. falciparum* cultures containing newly invaded ring-stage parasites were treated with **7a** and **7k** at fully growth-inhibitory concentrations (20 nM; at least 20 × EC_50_).

**Figure 4:**
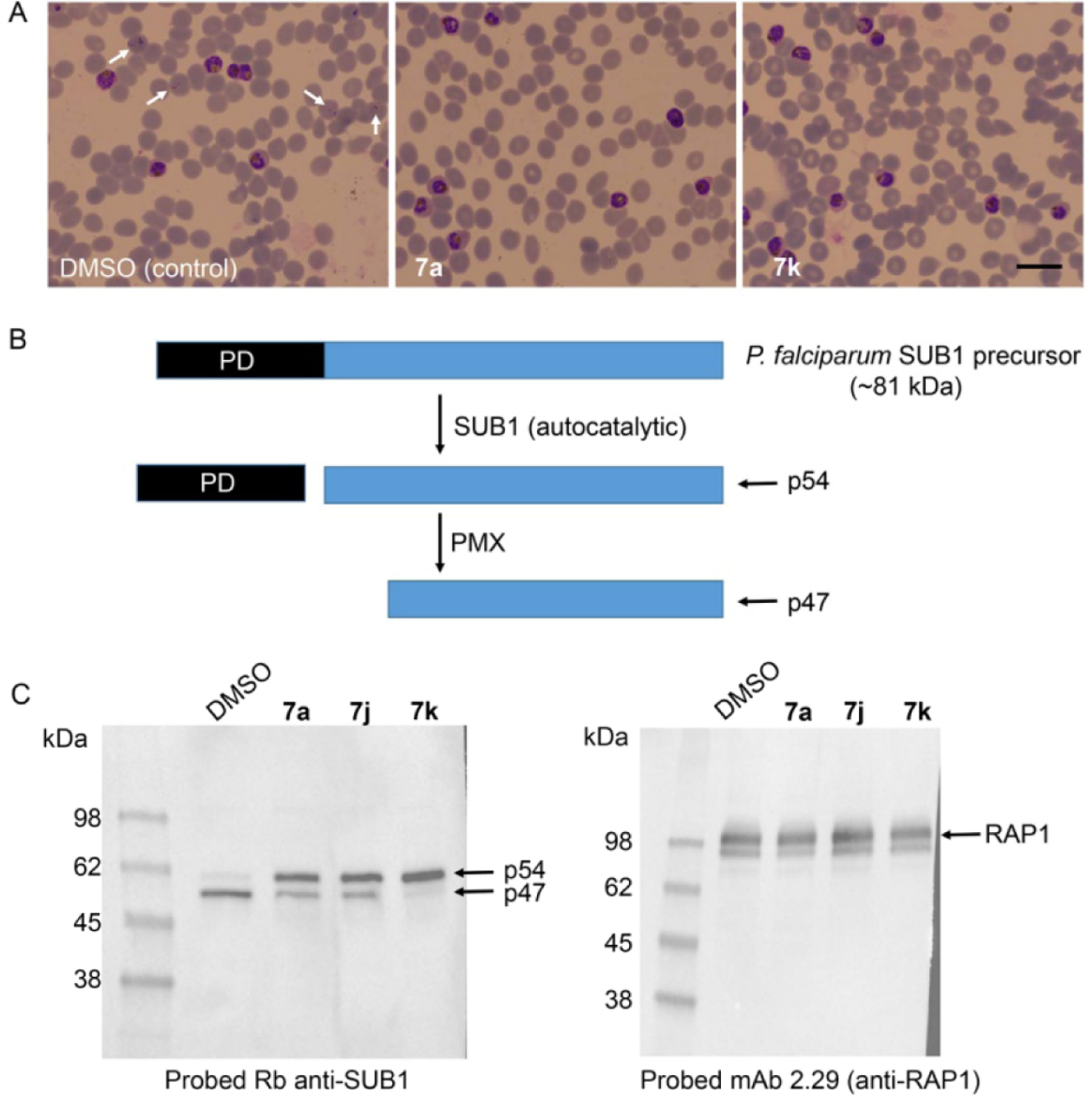
Macrocycle PMX inhibitors have no effect on intraerythrocytic parasite maturation but prevent egress and maturation of SUB1. (A) Light microscopic images of Giemsa-stained *P. falciparum* 3D7 parasites allowed to mature for 44 h in the presence of vehicle only (dimethyl sulfoxide (DMSO), control) or macrocycle compounds **7a** and **7k** (20 nM). Development of ring-stage parasites to the multinucleated schizont stage occurred similarly in all cultures. However, while new ring-stage parasites arising from successful egress and invasion were beginning to become evident by this time point in control cultures (DMSO panel, white arrows), schizont rupture and appearance of new rings did not occur in the cultures containing the PMX inhibitors, even following extended further incubation. Scale bar, 20 μm. (B) Simplified schematic of proteolytic maturation of the egress effector SUB1. Conversion of the precursor form to p54 is through autocatalytic removal^32^ of the prodomain region (PD), whereas conversion of p54 to p47 is mediated by PMX.^15,16,33^ (C) Left, Western blot analysis of *P. falciparum* 3D7 schizonts allowed to mature in the presence of the indicated macrocycle compounds (20 nM), showing defective SUB1 maturation in parasites treated with the indicated macrocycle compounds. Right, Western blot of the same extracts probed with a monoclonal antibody (mAb) specific to the rhoptry protein RAP1, which is a substrate for cleavage by the related *P. falciparum* protease PMIX. The compounds had no discernible effect on the maturation of RAP1. The results shown are typical of 3 independent experiments.

Microscopic monitoring of the treated cultures showed that even at these high compound concentrations, the parasites developed normally from rings to the multinucleated schizont stage in the presence of the compounds but then failed to rupture to allow merozoite egress and RBC invasion. This phenotype is characteristic of the inhibition of PMX. Furthermore, the lack of any effects on parasite development suggested the absence of significant off-target activity against other parasite pathways required for intraerythrocytic development. Consistent with this, Western blot analysis of schizonts isolated from cultures treated for 44 h as in Figure 4A with compounds **7a**, **7j**, and **7k** showed a clear defect in maturation of the PMX substrate SUB1, with an accumulation of the SUB1 p54 intermediate species rather than the mature SUB1 p47 form that predominates in control parasites as a result of PMX activity (Figure 4B and C). At the concentration used, inhibitor **7k** appeared to be more potent than compounds **7a** and **7j** in preventing SUB1 p54-to-p47 maturation, consistent with the lower IC_50_ and EC_50_ values for **7k** in the PMX enzyme inhibition and parasite growth assays, respectively.

We were unable to directly measure the effects of the compounds on PMIX activity due to recombinant enzymes not being available to us. However, none of the 3 macrocycles **7a**, **7j**, and **7k** had any impact on the profile of maturation of the parasite protein RAP, which is a substrate for the related plasmepsin PMIX, suggesting that the compounds do not affect PMIX activity at these concentrations.

**Figure 5:**
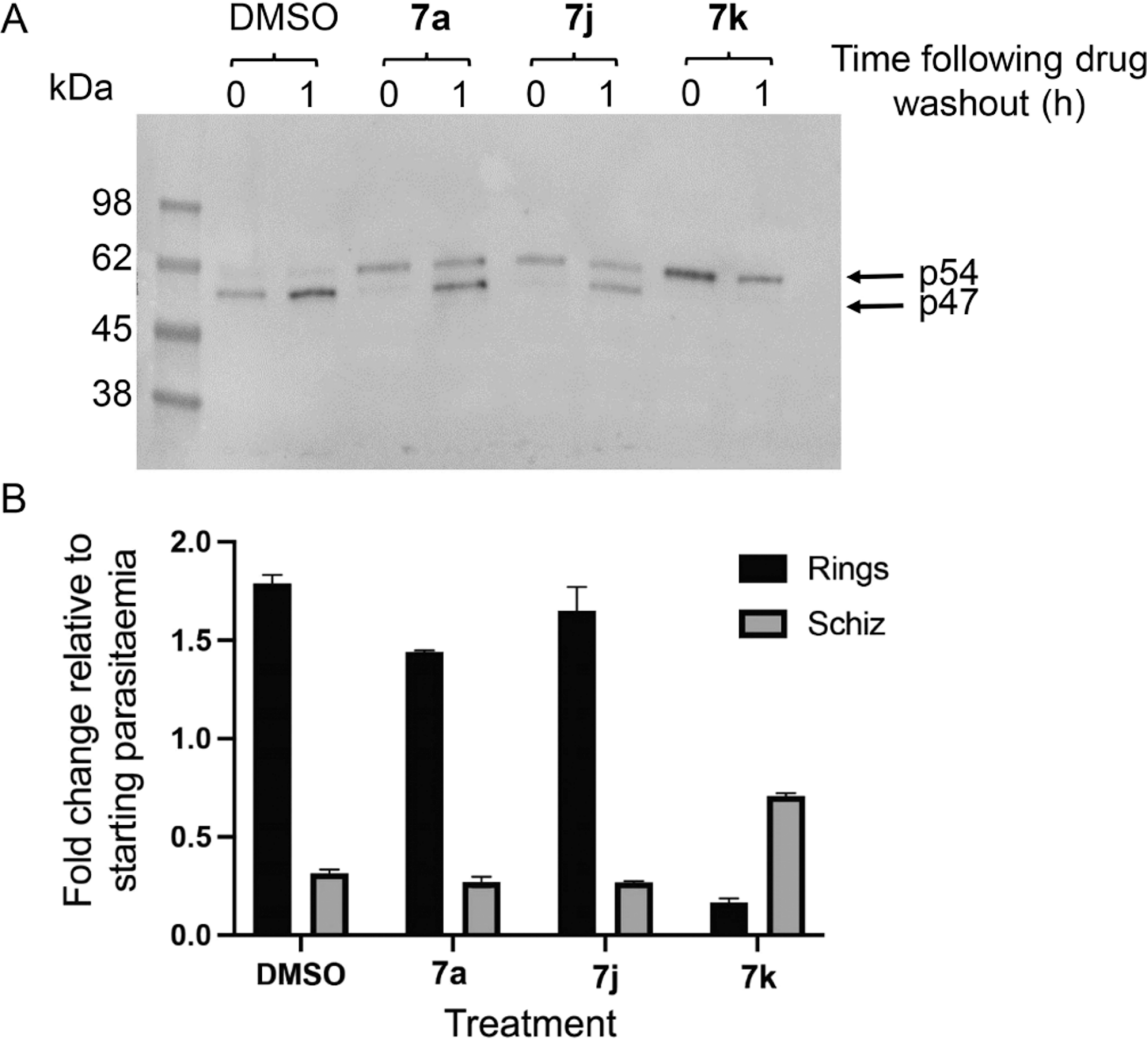
Washout experiments indicate low reversibility of PMX and egress inhibition mediated by macrocycle **7k**. (A) Washout of the macrocycle inhibitors allows restoration of SUB1 maturation in the case of compounds **7a** and **7j** but not in the case of **7k**. (B) Washout of the macrocycle inhibitors allows restoration of parasite egress (schizont rupture) and new ring formation in the case of compounds **7a** and **7j** but not in the case of **7k**. The plot shows both new ring formation and levels of residual unruptured schizonts after 3.5 h of further culture following washout.

To glean further insights into the mode and duration of action of the most potent macrocycle compounds, we used washout experiments to examine the reversibility of the egress and PMX inhibition mediated by the compounds. To do this, parasites treated with compounds **7a**, **7j**, and **7k** for the entire ∼44 h period of intraerythrocytic maturation, as in Figure 4A, were washed extensively to remove the drugs and then either immediately analyzed by Western blot or returned to culture, with or without the presence of added RBCs. The schizont cultures lacking added RBCs were supplemented with compound C2, which blocks the discharge of exonemes, the parasite secretory organelles in which PMX and SUB1 are stored before egress. The schizonts without added RBCs were sampled for Western blot analysis, while the schizont cultures containing RBCs were examined by microscopy and flow cytometry after a further 4 h in culture to quantify schizont rupture and new ring formation. As shown in Figure 5, the washout of compounds **7a** and **7j** in the presence of C2 resulted in subsequent efficient conversion of the accumulated SUB1 p54 intermediate to the p47 form, indicating reversal of the PMX inhibition mediated by these compounds. This presumably occurred in the exonemes. In contrast, no conversion of SUB1 p54 to p47 occurred following the washout of the most potent macrocycle **7k**. Remarkably, new ring formation from the washed schizonts was completely consistent with this pattern; while the washout of schizonts pretreated with **7a** and **7j** allowed an efficient generation of new rings, minimal rings were produced from schizonts following the washout of **7k**. These findings indicate effectively irreversible inhibition of SUB1 maturation and egress by compound **7k** over the time period examined, presumably due to continued blockade of PMX activity within the exonemes following washout. This could be due to a relatively long residence time of engagement of **7k** with its intracellular PMX target and/or inefficient washout of **7k** due to intracellular “trapping” of the compound.

### 2.5 Macrocyclization Leads to Improved Metabolic Stability

The primary motivation underlying our development of macrocyclic analogues of parent PMX inhibitor **6a** was to improve its drug-like properties. The metabolic stability was the most vulnerable point as inhibitor **6a** displayed a short half-life in the mice microsomal assay (Table 4, entry 1). Macrocyclic analogue **7a** showed slightly improved metabolic stability (Table 4, entry 2). *N*-Substitution at the amide group in compounds **7b**–**f** produced notable effects on metabolic stability *in vitro*. Removal of the *n*-propyl group improved stability (compound **7b**, Table 4, entry 3). Substitution with *N*-methyl or *N*-trifluoropropyl (compounds **7c,d**, Table 4, entries 4,5) increased the stability compared to compounds **6a** and **7a**, while the *N*-pivaloyl-substituted compound **7e** was only slightly better (Table 4, entry 6). A dimethylaminoethyl substituent (compound **7f**, Table 4, entry 7) dramatically improved the microsomal stability, which could be attributed to the charged nature of compound **7f** preventing its entry into microsomes. The length of the linker, shifting the oxygen in the linker by one position, or replacement of the oxygen with a methylene group had little effect on microsomal stability (compounds **7g**–**k**, Table 4, entries 8–12). Compared to parent compound **7a**, the macrocycle with a linker comprising only methylene groups (compound **7k**) showed increased stability (Table 4, entry 12).

**Table 4:** Microsomal Stability and Plasma Protein Binding

		microsomal stability^a^		plasma protein binding, %
#	cmpd.	half-life, min		CL(int), μL/min/mg
1	**6a**	6.40	216.40	99.1
2	**7a**	8.67	159.91	97.1
3	**7b**	31.27	44.32	97.26
4	**7c**	16.93	81.87	96.49
5	**7d**	20.41	67.91	98.75
6	**7e**	13.73	100.93	99.65
7	**7f**	>120	ND	93.05
8	**7g**	7.64	181.47	99.21
9	**7h**	13.89	99.78	96.16
10	**7i**	8.39	165.20	97.13
11	**7j**	14.37	96.43	99.33
12	**7k**	16.92	81.90	99.73

a Fluconazole (negative control) and propranolol (positive control).

Plasma protein binding by the parent compound **6a** was very high (Table 4, entry 1). Macrocyclic analogue **7a** showed reduced plasma protein binding (Table 4, entry 2), and modifications of the inhibitors leading to compounds **7c**, **7f**, and **7h** further reduced plasma protein binding (Table 4, entries 4,7,9). The most potent PMX and parasite growth-inhibitory inhibitor **7k** showed very high plasma protein binding, comparable to the parent compound **6a**.

### 2.6 *In Vivo* PK-Tox and *In Vivo* Antimalarial Potency of Inhibitor 7k

**Figure 6:**
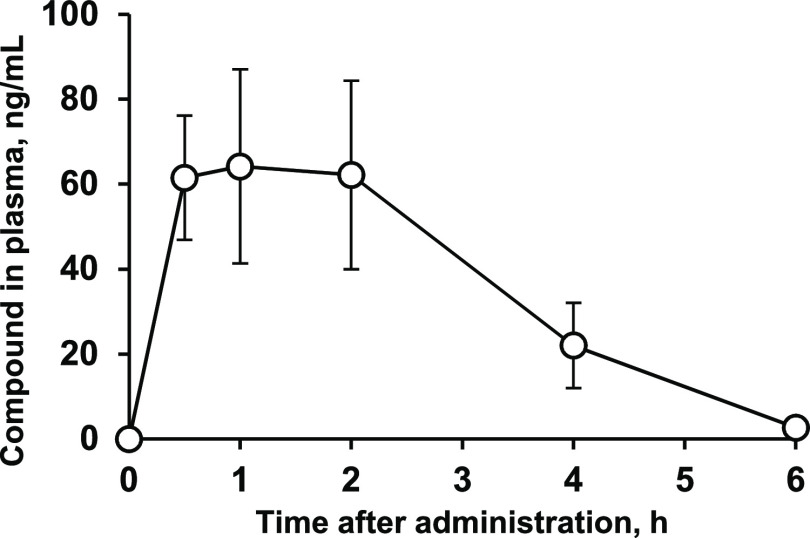
Plasma concentrations (±SEM) of inhibitor **7k** after p.o. administration in mice at a dose of 50 mg/kg.

Compound **7k** was well tolerated following oral administration of a dose of 50 mg/kg. No adverse effects were observed during a 24 h observation period (Table 5). The same dose was used in the pharmacokinetics (PK) experiment. Relative to the administered dose, low exposure was observed with a maximal concentration of compound **7k** (*C*_max_) of just 0.064 μg/mL (Figure 6). However, since this concentration exceeds concentrations that showed activity *in vitro*, antiparasitic activity *in vivo* was expected. Compound **7k** was rapidly cleared from the circulation (*t*_1/2_ = 53 min); thus, it was considered likely that multiple administrations per day might be required during efficacy testing *in vivo*.

**Table 5:** *In Vivo* PK-Tox Properties of Inhibitor **7k**

assay	result
tolerability p.os 50 mg/kg up to 24 h	no toxic effect (score 0)
PK p.os 50 mg/kg, data points 30, 60, 120, 240, 360 min^b^ NMRI female mice 10 weeks old ENVIGO, formulation: suspension, stabilized with HMC, 0.5%	*t*_1/2_ = 53 min
	*T*_max_ = 60 min
	*C*_max_ = 0.064 μg/mL
	AUC_0–*t*_ = 13.1 μg/mL*min
	AUC_0-inf_obs_ = 13.3 μg/mL*min

**Figure 7:**
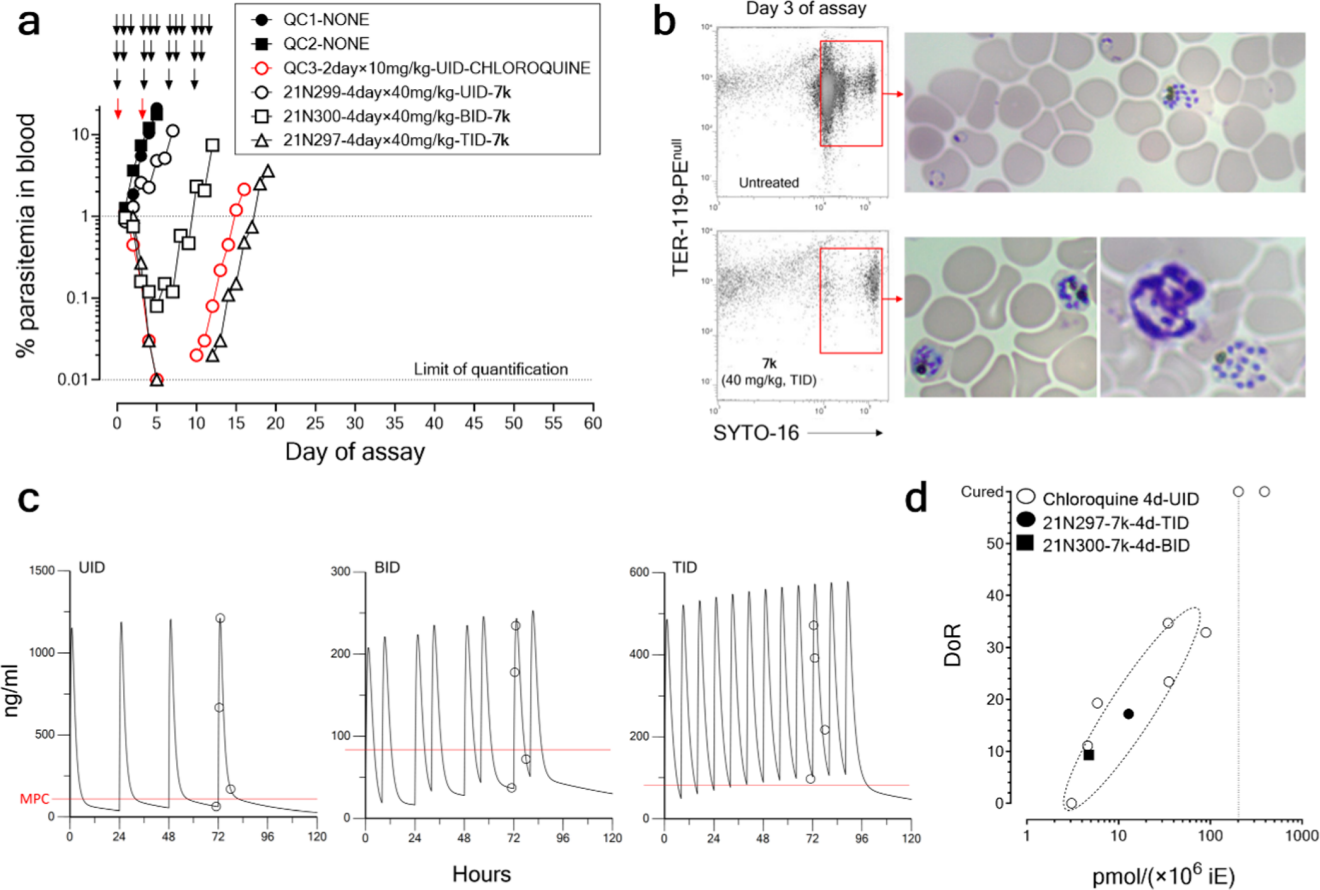
Therapeutic efficacy of inhibitor **7k** against *P. falciparumin vivo*. (a) Parasitemia in peripheral blood of PfalcHuMice: untreated (QC1 and QC2), treated with chloroquine, or treated with inhibitor **7k** at 40 mg/kg, p.o., UID, BID, or TID. (b) Distribution of stages of *P. falciparum* in peripheral blood of untreated and **7k**-treated PfalcHuMice after one parasite cycle of drug exposure (day 3 of the assay). The figure shows flow cytometry plots of human erythrocytes (not stained with the anti-mouse erythrocyte mAb TER-119 conjugated with phycoerythrine) stained with the nucleic acid dye SYTO-16. The red rectangles inside flow cytometry plots indicate the area of viable parasites. The photographs show Giemsa-stained blood smears of the very same cytometry plots representative of the parasite cells found in the corresponding regions of viability. (c) Pharmacokinetic modeling of the concentrations of inhibitor **7k** during the *in vivo* testing. Experimental data are represented by open symbols. The red lines indicate the preliminary estimate of minimal parasiticidal concentration (MPC) calculated from the experimental results. (d) PK/PD analysis of parasite killing *in vivo* induced by inhibitor **7k** in comparison with chloroquine as a standard reference antimalarial drug. The data shown in the plot are the day of recrudescence (DoR) *versus* the total exposure in the blood of **7k** of each individual mouse normalized by their respective individual parasite burdens as described.^34^ Data for Chloroquine are from historical data available at TAD for a set of PfalcHuMice treated p.o. UID, for 4 days, with different dose levels of the drug. In this plot, mapping to similar areas of the plot indicates equipotency for parasite killing in the PfalcHuMouse model.

Inhibitor **7k** was selected to execute a proof-of-concept (PoC) of the efficacy of the chemical family against asexual blood stages of *P. falciparumin vivo* in a standardized *P. falciparum* humanized mouse model (the PfalcHuMouse model). Given the low exposure expected for compound **7k** in PfalcHuMice, three dose regimens (QD, BID, and TID) were tested at a well-tolerated dose to maximize the time above a threshold efficacious concentration *in vivo*. As shown in Figure 7, inhibitor **7k** cleared parasitemia from the peripheral blood in a dose-dependent manner when administered orally. Importantly, the data showed that inhibitor **7k** mediated parasite clearance rates comparable to those produced by chloroquine at 10 mg/kg for 2 days in the same experimental system (Figure 7A). Flow cytometry and microscopy analysis showed a relative accumulation of circulating mature schizonts compared to untreated controls on day 3 of the assay (Figure 7B) after two cycles of drug exposure. This suggested that the schizont rupture and reinfection of RBCs were the most sensitive stages of the parasite to inhibitor **7k**. Pharmacokinetic modeling of the concentrations of inhibitor **7k** over the entire period of administration based on measurements of drug plasma concentration at 71.0, 72.5, 73.0, and 78.0 hours post-treatment suggested a rapid absorption with no accumulation (Figure 7C). Interestingly, inhibitor **7k** was shown to be as potent as chloroquine killing *P. falciparumin vivo* in the PfalchHuMouse model in comparable treatment regimens (ref Demarta-Gatsi et al. AAC. DOI: 10.1128/aac.01574-22) (Figure 7D).^34^ The total *C*_min_ in the individuals treated with **7k** were 51, 32, and 85 ng/mL for the individuals treated with UID, BID, and TID, respectively. A preliminary first estimate of the minimal parasiticidal concentration (MPC) of 85.3 ng/mL could be calculated since the individual treated BID showed the maximum parasite clearance rate for the first 48 h.

The PoC study allowed a preliminary PK/PD assessment of the antimalarial effect shown by **7k** in the PfalcHuMouse model (Figure 7D). We addressed this assessment by analyzing the day of recrudescence (DoR) as a function of the total exposure in the blood of the drugs tested, as recently described.^34^ DoR is defined as the the day at which parasitemia reaches again the parasitemia at treatment inception after a transient decline in parasitemia and is used as a measurement of parasite killing induced by the drugs. The amount of parasite killing (i.e., DoR) in one individual was correlated to the total exposure of the drug (**7k** and chloroquine as a comparator) in blood in the very same individual. In this study, the data of mice for which a DoR could be defined (mice treated with BID and TID **7k** at 40 mg/kg) were compared to historical data of Chloroquine, evaluated in the same experimental system and with the same methodology, available at The Art of Discovery.

Clearance of parasitemia *in vivo* by compound **7k** necessitated repeated administration of the compound due to its relatively short plasma lifetime (*t*_1/2_ = 53 min, Table 5), which could be attributed to low microsomal stability (Table 4, entry 12). For comparison, compound **7f**, which displays relatively high microsomal stability (Table 4, entry 7), was also investigated for antimalarial efficacy *in vivo* in the mouse model. However, treatment with compound **7f** three times a day for 4 days at 40 mg/kg (Supporting information, Figure S1) had only a weak effect on parasite burden.

## Conclusions

A series of potent, subnanomolar macrocyclic peptidic PMX inhibitors **7** has been developed that hold potential for development as a new class of antimalarial drugs that interrupt the malaria life cycle through a hitherto unexploited mode of action. Macrocyclic compounds were designed based on their acyclic analogues originating from a phenotypic screening hit by linking the S1 subpocket occupying the phenyl group with an amide residing in the S3 subpocket. Modification of the linker type and length led to compound **7k** with a 10-fold improved PMX potency. Compound **7k** showed low nanomolar potency also as an inhibitor of the hemoglobin degradative enzyme PMIV, but >100× selectivity against the human aspartic proteases CatD and BACE1. Compounds **7a,j,k** did not have an impact on the profile of maturation of the parasite protein RAP1, which is a substrate for the related plasmepsin PMIX, suggesting that the compounds do not affect PMIX activity. All of the compounds **7a**–**k** inhibited *in vitroP. falciparum* growth (3D7 and Dd2 strains), with compound **7k** being the most potent, consistent with the PMX inhibition data. Notably, there was a poor correlation between parasite growth-inhibitory activity and inhibition of the hemoglobin degradative enzyme PMIV. Macrocyclic inhibitor **7k** showed a 3-fold improved half-life in the microsomal stability test compared to the acyclic analogue **6a**. Another way to improve the microsomal stability was the installation of a dimethylaminoethyl group at the S3 amide substructure (compound **7f**). Both compounds **7f** and **7k** were investigated for their ability to eliminate *P. falciparum* from an infected humanized mice model. The less potent PMX inhibitor **7f** produced a measurable reduction in parasitemia when orally administered 3 times a day (40 mg/kg) for 4 days (see the Supporting Information). However, compound **7k**, when administered 3 times a day (40 mg/kg) for 4 days, eliminated *P. falciparum* from peripheral blood at a rate comparable to chloroquine single-dose administration for 2 days (10 mg/kg). The need for repeated administration was attributed to the relatively rapid clearance of compound **7k** (*t*_1/2_ = 53 min) as determined by PK assay in mice. In summary, we have developed an antimalarial lead compound **7k** acting as a PMX inhibitor, which demonstrates proof of principle for an orally administered drug in a *P. falciparum* infection model *in vivo*. Further development of analogues with a simplified route to synthesis and improved PK properties is now needed in order to achieve a next-generation lead suitable for a single-dose antimalarial treatment regimen.

## Experimental Section

### Antibodies and Reagents

The generation of polyclonal rabbit antibodies to *P. falciparum* SUB1 has been described previously.^35^ The RAP1-specific mouse mAb 2.29^36^ was a kind gift from the European Malaria Reagent Repository (www.malariaresearch.eu). The *P. falciparum* cGMP-dependent protein kinase (PKG) inhibitor C2^37,38^ was a kind gift from Simon Osborne, LifeArc.

### Maintenance of *P. falciparumIn Vitro* and Growth Assays

Asexual blood-stage forms of *P. falciparum* strains 3D7 and Dd2 were maintained at 37 °C in an atmosphere of 1% O_2_, 5% CO_2_, and 94% N_2_ in human RBCs in RPMI 1640 medium containing AlbuMAX II (Thermo Fisher Scientific) and supplemented with 2 mM l-glutamine. The synchronization of parasite cultures was as described previously.^39^ Briefly, this involved isolating mature schizont forms by centrifugation over cushions of 70% (v/v) isotonic Percoll (GE Healthcare, Life Sciences) and then allowing the parasites to undergo rupture in the presence of fresh RBCs for 2 h under gentle shaking conditions (100 rpm). Residual schizonts were then removed through a second Percoll separation, followed by treatment with 5% (w/v) sorbitol to finally obtain a highly synchronized preparation of newly invaded ring-stage parasites.

Growth assays were used to assess the capacity of the macrocycles to interfere with parasite proliferation *in vitro*. For this, synchronous cultures of ring-stage parasites (0.1% parasitemia, 2% hematocrit) were dispensed in duplicate into flat-bottomed 96-well plates (100 μL per well) containing 1 μL of test compounds serially diluted into DMSO such that final concentrations of the compounds ranged from 5 μM to 0.01 nM. Following culture for 96 h (a total of 2 erythrocytic cycles), samples from each well were fixed with an equal volume of 0.2% glutaraldehyde in phosphate-buffered saline (PBS) and stored at 4 °C until analysis by flow cytometry. Fixed parasites were stained with SYBR Green (Thermo Fisher Scientific, 1:10,000 dilution) for 20 min at 37 °C and then analyzed by flow cytometry on a BD FACSVerse using BD FACSuite software. Parasitemia was quantified by recording 10,000 events (cells) per well and filtering with appropriate forward and side scatter parameters and gating for SYBR Green stain-positive (infected RBCs) and negative RBCs using a 527/32 detector configuration. All data were analyzed using FlowJo software, and EC_50_ values were calculated in GraphPad Prism 8.0 using nonlinear regression, variable slope (four parameters). Growth stage progression and parasite morphology were also monitored by microscopic examination at selected time points using Giemsa-stained thin blood films.

### Washout Assays

Synchronous parasite cultures were cultured from ring to schizont stage (∼44 h) in the presence of DMSO alone (0.04% v/v; control) or macrocycle compounds (20 nM final concentration). Schizonts were isolated by Percoll enrichment (still in the presence of the test compound), then either immediately frozen or further incubated in the absence of the drug but in the presence of C2 (1 μM) for 1 h at 37°C in the absence of added RBCs or for 3.5 h in the presence of added RBCs. The schizonts without added RBCs were then frozen too, and all schizont samples were then thawed into CHAPS–urea extraction buffer, subjected to sodium dodecyl sulfate-polyacrylamide gel electrophoresis (SDS-PAGE), and analyzed by Western blot, probing with antibodies against SUB1 or RAP1. For schizonts cultured in the presence of added RBCs, ring formation was assessed in two ways: cultures were examined microscopically by Giemsa stain of thin films and were also fixed with glutaraldehyde and processed for quantification of ring formation by flow cytometry as described above.

### Enzyme Bioassays

A fluorescence resonance energy transfer (FRET)-based assay was performed to assess the ability of the compounds to inhibit PMIV, CatD, and BACE1. Serial dilutions of test compounds were dispensed in a white 96-well plate. Enzymes were then added: PMIV or CatD in 0.1 M NaOAc buffer, pH 4.5, 10% glycerol, 0.01% Tween 20, at final concentrations of 0.5 and 1.5 nM, respectively, or BACE1 was added to 0.1 M NaOAc buffer, pH 4.5, 0.01% Triton X-100 at a final concentration of 10 nM. The mixture was incubated for 20 min at 37 °C. PMIV and CatD substrate: DABCYL-ERNleFLSFP-EDANS (AnaSpec Inc.), a final concentration of 5 μM or BACE1 substrate: RE-EDANS-EVNLDAEFK-DABCYL-R (Calbiochem), a final concentration of 10 μM were then added. Substrate hydrolysis was detected as an increase in fluorescence at Em 490 nm, Ex 336 nm, 37 °C for 20 min and 60 min with BACE1. IC_50_ values were calculated with GraphPad Prism 8.0 using the nonlinear regression, variable slope (four parameters). Compounds were tested in triplicate.

### Recombinant *P. falciparum* PMX Inhibition Assays: Kinetics and IC_50_ Calculations

The proteolytic activity of PMX was quantified at room temperature by continuously monitoring the cleavage of the fluorogenic peptide substrate Rh2N (DABCYL-HSFIQEGKEE-EDANS) (Ex. 340, Em. 492). The fluorescence of this peptide is quenched by the spatial proximity of the DABCYL/EDANS groups in the uncleaved peptide and is increased upon cleavage at the internal F–I bond. The enzymatic reaction was performed in white Nunc 96-well microtiter plates by adding the following to each well: 50 μL of rPMX at a 0.72 nM intermediate dilution in 25 mM sodium acetate (pH 5.5), 0.005% Tween 20 digestion buffer (DB), and 1 μL of the inhibitor from a serial dilution ranging from 5 to 0.005 μM in 100% anhydrous DMSO. After an incubation time of 5 min to allow interaction of the inhibitors with PMX, 50 μL of the fluorogenic substrate Rh2N (3.2 μM intermediate dilution in DB) was added to the wells. Control wells contained no inhibitor, and the plate was blanked against the substrate only in DB. Subsequent fluorescence increase was monitored using a SpectraMax M5e plate reader and SoftMax Pro 7.1.0 software, with readings taken every 3 min for 60 min and using excitation and emission values of 340 and 492 nm, respectively. Initial rates were calculated over the first 28 min of the assay, during which period progress curves were linear, and IC_50_ values were calculated with GraphPad Prism 9.5.0 using the nonlinear regression, [inhibitor] vs response, variable slope (four parameters). All experiments were performed in duplicate.

### Docking of **7a** and **7k** into the PMX Crystallographic Structure

Flexible noncovalent docking of the macrocyclic compounds **7a** and **7k** was performed using ICM-Pro software (version 3.9-2e/MacOSX, Molsoft LLC). The inhibitors were drawn within the ICM molecule editor, converted to 3D, and appended to a chemical table for docking in batch. The WM382-inhibited X-ray structure (PDB ID: 7TBC) was converted to an ICM object for the docking procedure. In this process, hydrogen atoms were added to the PMX model, side chains of His, Pro, Asn, Gln, and Cys residues were optimized in an energetically favorable protonation state, and the WM382 inhibitor was used to define the PMX active site pocket. The WM382 inhibitor was then moved away from the receptor along with all water molecules. The potential energy maps of the PMX active site pocket and docking preferences were set up using the program default parameters. Energy terms were based on the all-atom vacuum force-field ECEPP/3, and conformational sampling was based on the biased probability Monte Carlo (BPMC) procedure.^40^ Two independent docking runs were performed per ligand, with a length of simulation (thoroughness) varying from 3 to 5 and the selection of 2 docking poses. Ligands were ranked according to their ICM energetics (ICM score, unitless), which weighs the internal force-field energy of the ligand combined with other ligand–receptor energy parameters.

### Liver Microsomal Metabolic Stability

Microsomal stability assays are performed as described elsewhere.^41^ Test compounds and pooled mouse liver microsomes are incubated in potassium phosphate buffer in the presence of the cofactor NADPH at 37 °C. Reaction mixtures (700 μL) contain a final concentration of 5 μM test compound, 0.05% DMSO, 0.5 mg/mL microsomes, and ∼0.7–0.9 mM NADPH in 100 mM potassium phosphate buffer at pH 7.4. Reactions are terminated by the addition of 100 μL of the reaction mixture to 900 μL of acetonitrile after 0, 5, 15, 30, 45, and 60 min of incubation. Samples were centrifuged, and the resultant supernatant was analyzed by ultra-performance liquid chromatography–tandem mass spectrometry (UPLC-MS/MS). The amount of remaining compound is calculated and compared to the initial amount of test compound (zero time point). Verapamil is used as a positive control, and samples prepared without cofactor NADPH are used as a negative control (0 and 60 min). The *in vitro* metabolic stability parameters (half-life value (*t*_1/2_) and intrinsic clearance value (Cl_int_)) are calculated from the slope of the Ln of concentration remaining vs the time curve.

### Plasma Protein Binding

Plasma protein binding was performed with rapid equilibrium dialysis (RED) device inserts containing a dialysis membrane with a molecular weight cut-off of 8000 Daltons. Inserts were placed in the single-use RED plate without prior preparation.

The test compound at a concentration of 5 μM was added to mouse plasma and dialyzed against phosphate-buffered saline (PBS, pH 7.4) for 2 h at 37 °C. Propranolol hydrochloride was used as a positive control (high PPB), and fluconazole was used as negative control (low PPB). After dialysis, the drug concentration in the buffer and plasma compartments was quantified by liquid chromatography and tandem mass spectrometry (LC-MS/MS) analysis.

### Tolerability and Pharmacokinetics Testing

NMRI female mice (10–12 weeks old, 29–32 g) were obtained from ENVIGO (The Netherlands) and housed prior to treatment under standard conditions (acclimatization period of 1 week, 21–23 °C, 12-h light/dark cycle, relative humidity 45–65%) with unlimited access to food (R70 diet from Lantmännen) and water. The experimental procedures were performed in accordance with the guidelines of the European Community and local laws and policies (Directive 2010/63/EU), and all of the procedures were approved by Food and Veterinary Service, Riga, Latvia. Animals were weighed on the day of the treatment (before treatment) to calculate the required amount of compound for the corresponding dose. For tolerability and PK studies, the mice were dosed via a peroral (PO) bolus administration (volume 10 mL/kg). Toxicity signs were scored 0–2, 5, 10, 15, 30, 60, 120, 240 min, and 24 h after administration using a widely used 0–6 score system.

For the pharmacokinetics experiment, blood samples were collected in tubes containing heparin. Blood was sampled from the tail vein 30, 60, 120, 240, and 360 min after the first and last administrations of the compounds. Tubes were centrifuged at +4 °C 10,000*g* for 3 min. Plasma samples were collected and stored at −20 °C until analysis.

### Quantitative Determination of VAD259 in Mouse Blood Plasma by UPLC/MS/MS

Concentrations of VAD259 in mouse plasma were measured by ultra-performance liquid chromatography–tandem mass spectrometry using Waters Acquity UPLC H-class chromatograph coupled to the Waters Xevo TQ-S micro mass spectrometer using the Waters Acquity UPLC BEH-C18 column (2.1 mm × 50 mm, 1.7 μm). Mobile phase A was 0.1% aqueous formic acid, and mobile phase B was acetonitrile. The solvent was pumped in gradient mode from 5% B to 98% B, the flow rate was 0.4 mL/min, and the method total run time was 5 min.

The mass spectrometer was operated in positive electrospray ionization mode (ESI+), and data acquisition was performed in multiple reaction monitoring (MRM) modes with ionization parameters as follows: capillary energy of 3.0 kV, cone voltage of 25 V, and collision energy of 20 eV. Specific MRM transition *m*/*z* 586.5 to *m*/*z* 438.3 was used for VAD259 quantification.

Blood plasma samples were processed using plasma protein precipitation. Then, 20 μL of plasma was mixed with 300 μL of acetonitrile/methanol (3:1/v:v) mixture, and the sample was centrifuged at 1000*g* for 10 min. The supernatant was further diluted 3 times with 0.1% aqueous formic acid and subjected to UPLC/MS/MS analysis.

### *In Vivo* Efficacy against *P. falciparum* in the TAD PfalcHuMouse Model

Therapeutic efficacy studies were performed at TAD using a standardized humanized mouse model of *P. falciparum* malaria^31^ with modifications.^34^ Briefly, 22–28 g female NOD-SCID IL-2Rγnull mice (NSG) (Charles River, France) were engrafted with human erythrocytes (hE) (Basque Center of Transfusion and Human Tissues, Galdakao, Spain, Centro de Transfusiones de la Comunidad de Castilla y León, Valladolid, Spain and Bank of Blood and Tissues, Barcelona, Spain) by intraperitoneal (i.p.) and/or intravenous (i.v., via tail lateral vein) daily inoculation of 50–75% hematocrit cell suspensions in RPMI 1640 medium, 25% (vol/vol) decomplemented human serum, and 3.1 mM hypoxanthine. The volume of injections was 1 and 0.7 mL for i.p. or i.v. inoculation, respectively.

When engrafted mice (i.e., HuMice) had more than 40% of circulating hE in peripheral blood, HuMice were infected with *P. falciparum* Pf3D7^0087/N9^ by inoculation of 0.3 mL of an erythrocyte suspension containing 1.17 × 10^8^ erythrocytes parasitized per mL via the lateral vein. The inoculum was obtained from peripheral blood of CO_2_-euthanized donor mice harboring 5–10% parasitemia.

Before drug administration, each infected mouse was randomly assigned to its corresponding treatment. Drug treatment started at ∼1.3% patent parasitemia (Day 1). The treatment was administered by oral gavage with 20G straight reusable feeding needles (Fine Science Tools GmbH) at 10 mL·kgbodyweight^–1^ unless otherwise stated.

Parasitemia was measured in serial 2 μL of samples of tail blood by flow cytometry and expressed as the % of parasitized erythrocytes with respect to the total erythrocytes in circulation. A qualitative analysis of the effect of treatment on *P. falciparum* Pf3D7^0087/N9^ was assessed by microscopy with Giemsa-stained blood smears and flow cytometry by staining with TER-119-Phycoerythrine (a marker of murine erythrocytes) and SYTO-16 (nucleic acid dye) and acquisition in an Attune NxT Acoustic Focusing Flow Cytometer (Invitrogen), as previously described.^42^

The concentrations of drugs were measured in samples of peripheral blood (25 μL) taken at different times after the first dosing, mixed with 25 μL of Milli-Q H_2_O, and immediately frozen on a thermal block at −80 °C. The frozen samples were stored at −80 °C until analysis. Blood from control humanized mice was used for the preparation of standard curves, calibration, and quality control purposes. The drugs were extracted from 10 μL of lysates obtained by protein precipitation of blood samples using standard liquid–liquid extraction methods. The samples were analyzed by LC-MS/MS for quantification in a Waters Micromass UPLC-TQD (Waters, Manchester, U.K.). Blood concentration vs time was analyzed by non-compartmental analysis (NCA) using Phoenix WinNonlin vers.9.2 (Certara) or R or Excel (Microsoft), from which exposure-related values (*t*_max_, *C*_max_, and AUC_0–*t*_) were estimated.

The clearance of parasitized erythrocytes from the peripheral blood of mice was assessed by measuring the parasite reduction ratio (PRR) calculated as the ratio between parasitemia at Day *n* + 2 divided by parasitemia at Day *n* for each individual of the study.

The parasiticidal effect of drugs *in vivo* was assessed by measuring the day of recrudescence (DoR) as described.^34^ In short, DoR was defined as the day at which parasitemia after drug treatment reached the % parasitemia at treatment inception. Drug-treated mice were deemed cured if there was no detectable parasitemia 60 days after treatment.

### Ethical approvals

The studies were approved by The Art of Discovery Institutional Animal Care and Use Committee (TAD-IACUC), certified by the Biscay County Government (Bizkaiko Foru Aldundia, Basque Country, Spain) to evaluate animal research projects from Spanish institutions according to point 43.3 from Royal Decree 53/2013, from the 1st of February (BOE-A-2013–1337). All experiments were carried out in accordance with European Directive 2010/63/EU. The results from the animal experiments were reported following ARRIVE guidelines (https://www.nc3rs.org.uk/arrive-guidelines), except for disclosure of business trade confidential information. The human biological samples were sourced ethically, and their research use was in accord with the terms of the informed consent.

### Chemistry

#### General

All chemicals were used as obtained from commercial sources, and all reactions were performed under an argon atmosphere in oven-dried (120 °C) glassware unless noted otherwise. Anhydrous toluene, Et_2_O, tetrahydrofuran (THF), and CH_2_Cl_2_ were obtained by passing commercially available anhydrous solvents through activated alumina columns.

NMR spectra were recorded on 300 and 400 MHz spectrometers with chemical shift values (δ) in ppm using the residual solvent signal as an internal standard. UPLC/MS analysis was performed on a Waters Acquity column: Acquity UPLC BEH-C18 (2.1 mm × 50 mm, 1.7 μm, (30.0 ± 5.0) °C); gradient, 0.01% TFA in water/CH_3_CN 90/10% to 10/90%; flow rate, 0.5 mL/min; run time, 8 min; detector, PDA (photodiode matrix), 220–320 nm, SQ detector with an electrospray ion source (APCI). Analytical thin-layer chromatography (TLC) was performed on precoated silica gel F-254 plates. High-resolution mass spectra (HRMS) were recorded on a Waters Synapt G2-Si TOF MS instrument using the ESI technique. Specific rotation was recorded on a Kruss P3000 instrument. Purification by preparative reverse phase chromatography was performed using Waters Atlantis Prep OBD T3 Column 30 mm × 100 mm, 5 μm. The purity of all target inhibitors was confirmed to be ≥95% by the reversed-phase high-performance liquid chromatography (HPLC) assay.

### General Procedure A for Boc-Deprotection of Amines

To a solution of Boc-protected amine (1 equiv) in anhydrous dichloromethane (DCM) (1.5 mL/1 mmol of the substrate), trifluoroacetic acid (20 equiv) was added, and the reaction mixture was stirred at room temperature for 1 h. All volatiles were removed under reduced pressure. Then, 1 M aqueous NaOH solution (20 mL) was added to the residue. The mixture was extracted with diethyl ether (3 × 20 mL). The combined organic phase was washed with brine, dried over anhydrous Na_2_SO_4_, and evaporated under reduced pressure.

### General Procedure B for Silylation of the Secondary Hydroxyl Group

To a stirred solution of alcohol (1 equiv) in *N*,*N*-dimethylformamide (DMF) (8.5 mL/1 mmol of alcohol), imidazole (5–10 equiv) and TBSCl (5–10 equiv) were added. The reaction mixture was stirred at 80 °C overnight. DMF was evaporated under reduced pressure. The residue was dissolved in EtOAc (20 mL) and water (20 mL). The aqueous phase was separated and washed with EtOAc (2 × 20 mL). The combined organic phase was washed with brine (20 mL), dried over anhydrous Na_2_SO_4_, and evaporated under reduced pressure.

### General Procedure C for the Intermolecular Mitsunobu Reaction and Denosylation of the Amino Group

To a solution of phenol (1 equiv) and aliphatic alcohol (3–5 equiv) in anhydrous DCM (3.7 mL/0.1 mmol of the phenol), PPh_3_ (3–5 equiv) was added, followed by either diethyl azodicarboxylate (DEAD) or di-*tert*-butyl azodicarboxylate (DTBAD) (3–4 equiv). The reaction mixture was stirred at room temperature for 2 h. The reaction mixture was evaporated, and the residue was purified by silica gel column chromatography using diethyl ether. Fractions with product were combined and evaporated under reduced pressure. The residue was used in the next step without further purification.

To a solution of protected amine from the previous step in MeCN (3 mL/0.1 mmol of the amine), K_2_CO_3_ (4–10 equiv) was added, followed by thiophenol (2–5 equiv). The reaction mixture was stirred at room temperature for 2 h. The reaction mixture was diluted with DCM (20 mL) and washed with water (20 mL) and 0.1 M NaOH solution (20 mL). The aqueous phase was extracted with DCM (2 × 25 mL). The combined organic phase was washed with brine, dried over anhydrous Na_2_SO_4_, and evaporated. A pure material was obtained by column chromatography using gradient elution from DCM to 5% MeOH in DCM.

### General Procedure D for Hydrolysis of Methyl Benzoates

One molar aqueous NaOH solution (2–6 equiv) was added to a solution of benzoate (1.0 equiv) in THF (2.5 mL/0.1 mmol of the benzoate). The resulting solution was stirred until the conversion of the starting material was complete. The solvent was evaporated under reduced pressure.

### General Procedure E for Macrolactamization

To a solution of deprotected amino acid (1 equiv) in anhydrous DMF (1 mL/0.01 mmol of the amino acid), HBTU (1.5 equiv) was added. The reaction mixture was stirred at room temperature for 1 h. DMF was evaporated under reduced pressure. The residue was suspended in DCM and filtered through a small silica column, using gradient elution from DCM to 10% MeOH in DCM.

### General Procedure F for the Desilylation of Secondary Alcohols

To a solution of silylated macrocycle (1 equiv) in MeOH (3 mL/0.01 mmol of the macrocycle), NH_4_F (20–30 equiv) was added. The reaction mixture was stirred until complete conversion of the starting material was observed in HPLC. The reaction mixture was evaporated under reduced pressure.

### General Procedure G for the Synthesis of Amides

A solution of carboxylic acid (1–2 equiv) in anhydrous DCM (1 mL/0.1 mmol of the acid) was cooled to 0 °C. *N*,*N*-diisopropylethylamine (DIPEA, 1–4 equiv) was added, followed by 2-(1H-benzotriazol-1-yl)-1,1,3,3-tetramethyluronium hexafluorophosphate (HBTU, 1–2 equiv). The reaction mixture was stirred at 0 °C for 10 min. A solution of amine (1–2 equiv) in DCM (1 mL/0.2 mmol) was added, and the reaction mixture was allowed to warm up to room temperature and stirred for 30 min. The solvent was evaporated under reduced pressure. The residue was dissolved in EtOAc (40 mL), and the solution was washed with 1 M HCl (40 mL), 1 M NaOH (40 mL), and brine (40 mL), dried over anhydrous Na_2_SO_4_, and evaporated under reduced pressure.

### General Procedure H for the Debenzylation of Alcohols

To a solution of benzyl ether (1 equiv) in MeOH (9 mL/0.1 mmol of the benzyl ether), 10% Pd on carbon (0.1–0.2 equiv) was added, followed by formic acid (2.2 mL/0.1 mmol of the benzyl ether). The reaction mixture was stirred under a hydrogen atmosphere (1–6 atm). After the reaction was complete, the reaction mixture was filtered through a syringe filter (Teflon; 0.45 μm, washed with EtOAc). The filtrate was washed with 12% aqueous ammonia solution (18 mL/0.1 mmol of the benzyl ether). EtOAc was added until two layers were visible. Phases were separated, and the aqueous phase was extracted with EtOAc (2 × 30 mL). The combined organic phase was washed with brine, dried over anhydrous Na_2_SO_4_, and evaporated. The full conversion was achieved after stirring under a hydrogen atmosphere (1 atm) for 3 h. A pure material was obtained by column chromatography on silica gel using gradient elution from DCM to 10% MeOH in DCM.

### General Procedure I for the Intramolecular Mitsunobu Reaction (Cyclization)

To a solution of diol (1 equiv) in anhydrous DCM (8 mL/0.1 mmol of the diol), PPh_3_ (2–3 equiv) was added, followed by DTBAD (2–3 equiv). The reaction mixture was stirred at room temperature for 15 min. The reaction mixture was evaporated under reduced pressure, and the residue was purified by silica gel column chromatography using diethyl ether. Fractions with product were combined and evaporated under reduced pressure. The residue was used in the next step without further purification.

### General Procedure J for the Ring-Closing Metathesis and the Hydrogenation of Double Bond

To a solution of bis-alkene (1 equiv) in anhydrous toluene (4.4 mL/0.01 mmol of the bis-alkene), Zhan Catalyst-1B (0.1 equiv) was added. The reaction mixture was stirred at 55 °C overnight. The reaction mixture was evaporated under reduced pressure. The residue was filtered through silica using 30% EtOAc in hexanes, evaporated under reduced pressure, and the residue was used in the next step without further purification.

The residue from the previous step was dissolved in MeOH (8.7 mL/0.1 mmol of the bis-alkene). Then, 10% Pd on carbon (0.1 equiv) was added, and the reaction mixture was stirred under a hydrogen atmosphere (1 atm). After the reaction was complete, the reaction mixture was filtered through a syringe filter (Teflon; 0.45 μm, washed with MeOH).

#### (*S*)-*N*-((*E*)-((*S*)-2,2-Dimethyl-1,3-dioxolan-4-yl)methylene)-2-methylpropane-2-sulfinamide (**10**)

To a stirred solution of tert-butylsulfinamide (**9)** (3.49 g, 28.8 mmol, 1.5 equiv) in anhydrous DCM (200 mL) was added glyceraldehyde **8** (5.00 g, 19.2 mmol, 1 equiv). Ti(OEt)_4_ (20.1 mL, 96.0 mmol, 5 equiv) was added, and the reaction mixture was stirred at room temperature for 1 h. The mixture was cooled in an ice bath for 5 min. Deionized water (30 mL) was added. After stirring for 5 min, the mixture was filtered through Celite, washing with EtOAc. The filtrate was concentrated under reduced pressure, washed with brine (50 mL), dried over anhydrous Na_2_SO_4_, and evaporated under reduced pressure. Purification by column chromatography on silica gel using 10% EtOAc in hexanes afforded product **10** as a yellow oil (3.37 g, 75% yield). ^1^H NMR (300 MHz, chloroform-*d*): δ 8.07 (d, *J* = 4.1 Hz, 1H), 4.84 (ddd, *J* = 6.8, 5.1, 4.1 Hz, 1H), 4.22 (dd, *J* = 8.5, 6.8 Hz, 1H), 4.04 (dd, *J* = 8.5, 5.1 Hz, 1H), 1.45 (s, 3H), 1.42 (s, 3H), 1.20 (s, 9H). Corresponds with the literature.^43^

#### (*S*)-*N*-((*S*)-2-(3-(Benzyloxy)phenyl)-1-((*S*)-2,2-dimethyl-1,3-dioxolan-4-yl)ethyl)-2-methylpropane-2-sulfinamide (**11**)

A solution of imine **10** (1.70 g, 7.30 mmol, 1 equiv) in THF (7.3 mL) was cooled to −78 °C in a dry ice/acetone bath. Freshly prepared 0.7 M 3-(benzyloxy)benzylmagnesium chloride solution in THF (10.4 mL, 7.30 mmol, 1 equiv) was added dropwise. The reaction mixture was allowed to slowly warm up to room temperature and stirred for 1 h. The reaction mixture was quenched with sat. aq. NH_4_Cl solution (25 mL) and extracted with EtOAc (3 × 50 mL). The combined organic phase was washed with brine (25 mL), dried over anhydrous Na_2_SO_4_, and evaporated under reduced pressure. Purification by column chromatography on silica gel using 90% DCM/9% EtOAc/1% MeOH afforded product **11** as a yellow oil (2.07 g, 66% yield). ^1^H NMR (400 MHz, chloroform-*d*): δ 7.45–7.35 (m, 4H), 7.35–7.29 (m, 1H), 7.20 (dd, *J* = 9.0, 7.5 Hz, 1H), 6.86–6.76 (m, 3H), 5.05 (s, 2H), 4.15 (td, *J* = 6.3, 5.1 Hz, 1H), 4.05 (dd, *J* = 8.5, 6.6 Hz, 1H), 3.95 (dd, *J* = 8.5, 6.1 Hz, 1H), 3.66 (dtd, *J* = 7.3, 6.2, 5.1 Hz, 1H), 3.55 (d, *J* = 6.1 Hz, 1H), 2.99 (dd, *J* = 13.9, 7.3 Hz, 1H), 2.72 (dd, *J* = 13.9, 6.3 Hz, 1H), 1.47 (s, 3H), 1.32 (s, 3H), 1.11 (s, 9H). ^13^C NMR (101 MHz, Chloroform-*d*): δ 159.0, 139.1, 137.1, 129.6, 128.7, 128.1, 127.5, 122.3, 116.3, 113.1, 109.5, 77.2, 70.0, 65.9, 58.5, 56.2, 37.8, 26.6, 24.93, 22.6. HRMS-ESI (*m*/*z*) calcd for C_24_H_34_NO_4_S [M + H]^+^ 432.2209. Found 432.2202. [α]_D_^20^ = 30.4 (*c* 1.0, CHCl_3_).

#### *tert*-Butyl ((2*S*,3*S*)-1-(3-(Benzyloxy)phenyl)-3,4-dihydroxybutan-2-yl)carbamate (**12**)

To a stirred solution of compound **11** (2.25 g, 5.21 mmol, 1 equiv) in diethyl ether (50 mL), 4 M HCl solution in dioxane (2.61 mL, 10.4 mmol, 2 equiv) was added, and the reaction mixture was stirred at room temperature for 20 min. A precipitate was filtered, and the filtered solid was dissolved in MeOH (50 mL). Then, 4 M HCl solution in dioxane (2.61 mL, 10.4 mmol, 2 equiv) was added, and the reaction mixture was stirred at room temperature overnight. All volatiles were removed under reduced pressure. The residue was dissolved in DCM (50 mL). DIPEA (3.61 mL, 20.9 mmol, 4 equiv) was added, followed by a solution of (Boc)_2_O (1.82 g, 8.34 mmol, 1.6 equiv) in DCM (10 mL). The reaction mixture was stirred at room temperature for 1 h. Imidazole (2.13 g, 31.3 mmol, 6 equiv) was added. The reaction mixture was stirred at room temperature for 20 min. The mixture was washed with 1% HCl solution (3 × 50 mL) and brine (30 mL) and dried over anhydrous Na_2_SO_4_. Evaporation under reduced pressure afforded product **12** as a beige solid (1.80 g, 89% yield). ^1^H NMR (400 MHz, chloroform-*d*): δ 7.48–7.29 (m, 5H), 7.25–7.21 (m, 1H), 6.85 (ddt, *J* = 12.5, 7.5, 1.3 Hz, 3H), 5.06 (s, 2H), 4.58 (d, *J* = 8.5 Hz, 1H), 3.83 (qd, *J* = 8.5, 4.4 Hz, 1H), 3.73–3.54 (m, 2H), 3.32 (d, *J* = 8.5 Hz, 1H), 3.05 (dd, *J* = 14.2, 4.4 Hz, 1H), 2.90 (dd, *J* = 14.3, 7.5 Hz, 1H), 2.68 (br s, 2H), 1.39 (s, 9H). ^13^C NMR (101 MHz, chloroform-*d*): δ 159.2, 157.2, 139.0, 137.1, 129.8, 128.7, 128.1, 127.7, 122.2, 116.2, 113.2, 80.6, 73.1, 70.1, 63.0, 52.2, 36.6, 28.4. HRMS-ESI (*m*/*z*) calcd for C_22_H_29_NO_5_Na [M+Na]^+^ 410.1943. Found 410.1942. [α]_D_^20^ = 10.5 (*c* 1.4, CHCl_3_).

#### *tert*-Butyl ((2*S*,3*R*)-1-(3-(Benzyloxy)phenyl)-3-hydroxy-4-((2-(3-methoxyphenyl)propan-2-yl)amino)butan-2-yl)carbamate (**15**)

The diol **12** (2.10 g, 5.42 mmol, 1 equiv) was dissolved in chloroform (50 mL) in a pressure tube. Triphenylphosphine (2.13 g, 8.13 mmol, 1.5 equiv) was added, followed by di-*tert*-butyl azodicarboxylate (1.87 g, 8.13 mmol, 1.5 equiv). The tube was sealed, and the reaction mixture was stirred at 80 °C overnight. Chloroform was evaporated under reduced pressure, and the residue was diluted with dry *i*-PrOH (5 mL). Amine **14** (2.69 g, 16.3 mmol, 3 equiv) was added, and the reaction mixture was stirred at 80 °C for 5 h. *i*-PrOH was evaporated under reduced pressure. Purification by column chromatography on silica gel using diethyl ether afforded product **15** as a yellow oil (1.80 g, 62% yield). Excess of amine **14** was recovered by the washing column with EtOAc. ^1^H NMR (400 MHz, chloroform-*d*): δ 7.45–7.41 (m, 2H), 7.40–7.35 (m, 2H), 7.34–7.28 (m, 1H), 7.23 (t, *J* = 8.1 Hz, 1H), 7.19 (t, *J* = 7.8 Hz, 1H), 7.03–6.95 (m, 2H), 6.86–6.72 (m, 4H), 5.04 (s, 2H), 4.57 (d, *J* = 9.2 Hz, 1H), 3.80 (s, 3H), 3.79–3.73 (m, 2H), 3.28 (dt, *J* = 7.6, 4.5 Hz, 1H), 2.92 (dd, *J* = 14.1, 4.7 Hz, 1H), 2.81 (dd, *J* = 14.2, 7.6 Hz, 1H), 2.45 (dd, *J* = 11.6, 4.3 Hz, 1H), 2.37 (dd, *J* = 12.5, 3.4 Hz, 1H), 1.45 (s, 6H), 1.37 (s, 9H). ^13^C NMR (101 MHz, chloroform-*d*): δ 159.7, 159.0, 156.2, 149.3, 139.6, 137.2, 129.5, 129.3, 128.7, 128.0, 127.7, 122.3, 118.5, 116.1, 112.9, 112.6, 111.2, 79.6, 71.2, 70.0, 55.7, 55.3, 53.6, 44.4, 36.8, 29.7, 29.6, 28.5. HRMS-ESI (*m*/*z*) calcd for C_32_H_43_N_2_O_5_ [M + H]^+^ 535.3172. Found 535.3179. [α]_D_^20^ = −10.5 (*c* 1.2, CHCl_3_).

#### *tert*-Butyl ((2*S*,3*R*)-1-(3-(Benzyloxy)phenyl)-3-((*tert*-butyldimethylsilyl)oxy)-4-((2-(3-methoxyphenyl)propan-2-yl)amino)butan-2-yl)carbamate (**16**)

A solution of **15** (4.63 g, 8.66 mmol, 1 equiv), imidazole (5.90 g, 86.8 mmol, 10 equiv), and TBSCl (13.1 g, 86.8 mmol, 10 equiv) in DMF (60 mL) was stirred at 50 °C overnight. DMF was evaporated under reduced pressure. The residue was dissolved in EtOAc (30 mL) and water (30 mL). The aqueous phase was separated and extracted with EtOAc (2 × 20 mL). The combined organic phase was washed with brine (20 mL), dried over anhydrous Na_2_SO_4_, and evaporated under reduced pressure. Purification by column chromatography on silica gel using gradient elution from 5 to 30% EtOAc in hexanes afforded product **16** as a colorless oil (4.63 g, 82%). ^1^H NMR (400 MHz, chloroform-*d*): δ 7.45–7.41 (m, 2H), 7.38 (ddd, *J* = 7.5, 6.6, 1.3 Hz, 2H), 7.34–7.29 (m, 1H), 7.29–7.23 (m, 1H), 7.20–7.13 (m, 1H), 7.06–6.96 (m, 2H), 6.78 (ddd, *J* = 11.1, 5.9, 2.1 Hz, 4H), 6.52–6.24 (m, 1H), 5.03 (s, 2H), 4.07–3.92 (m, 1H), 3.80 (s, 3H), 3.65 (d, *J* = 20.3 Hz, 1H), 2.83 (dd, *J* = 14.0, 7.7 Hz, 1H), 2.54 (dd, *J* = 15.0, 8.0 Hz, 1H), 2.49 (br s, 1H), 1.69–1.57 (m, 2H), 1.46 (s, 3H), 1.44 (s, 3H), 1.42 (s, 9H), 0.87 (s, 9H), −0.02 (s, 3H), −0.07 (s, 3H). ^13^C NMR (101 MHz, chloroform-*d*): δ 159.8, 159.0, 156.0, 149.3, 140.5, 137.3, 129.4, 129.3, 128.7, 128.0, 127.6, 121.9, 118.5, 115.6, 112.8, 112.5, 111.3, 78.6, 71.3, 70.0, 56.5, 55.6, 55.3, 44.8, 37.6, 29.7, 29.3, 28.6, 26.0, 18.2, −4.7, −4.8. HRMS-ESI (*m*/*z*) calcd for C_38_H_57_N_2_O_5_Si [M + H]^+^ 649.4037. Found 649.4049. [α]_D_^20^ = −26.3 (*c* 1.5, CHCl_3_).

#### (2*R*,3*S*)-3-Amino-4-(3-(benzyloxy)phenyl)-1-((2-(3-methoxyphenyl)propan-2-yl)amino)butan-2-ol (**17**)

The title compound was obtained as a yellow oil (420 mg, 94% yield) from carbamate **15** (550 mg, 1.03 mmol, 1 equiv) and trifluoroacetic acid (1.58 mL, 20.6 mmol, 20 equiv), following general procedure A. A pure material was obtained by column chromatography using gradient elution from DCM to 5% MeOH in DCM. ^1^H NMR (400 MHz, methanol-*d*_4_): δ 7.44–7.37 (m, 2H), 7.37–7.31 (m, 2H), 7.31–7.26 (m, 1H), 7.26–7.20 (m, 1H), 7.20–7.13 (m, 1H), 7.03–6.97 (m, 2H), 6.84–6.79 (m, 2H), 6.78–6.71 (m, 2H), 5.04 (s, 2H), 3.77 (s, 3H), 3.51 (ddd, *J* = 8.6, 5.2, 3.4 Hz, 1H), 2.95 (dt, *J* = 9.0, 4.9 Hz, 1H), 2.79 (dd, *J* = 13.5, 4.8 Hz, 1H), 2.49 (dd, *J* = 11.7, 3.4 Hz, 1H), 2.38 (ddd, *J* = 17.0, 12.6, 8.8 Hz, 2H), 1.45 (s, 3H), 1.45 (s, 3H). ^13^C NMR (101 MHz, methanol-*d*_4_): δ 161.2, 160.3, 150.1, 142.1, 138.7, 130.6, 130.4, 129.5, 128.9, 128.6, 123.0, 119.4, 116.9, 113.9, 113.2, 112.6, 74.5, 70.9, 57.3, 56.9, 55.8, 46.2, 40.1, 30.2, 29.0. HRMS-ESI (*m*/*z*) calcd for C_27_H_35_N_2_O_3_ [M + H]^+^ 435.2648. Found 435.2644. [α]_D_^20^ = 2.9 (*c* 0.7, CHCl_3_).

#### (2*R*,3*S*)-4-(3-(Benzyloxy)phenyl)-2-((*tert*-butyldimethylsilyl)oxy)-*N*^1^-(2-(3-methoxyphenyl)propan-2-yl)butane-1,3-diamine (**18**)

The title compound was obtained as a white solid foam (2.25 g, 92% yield) from carbamate **16** (2.90 g, 4.47 mmol, 1 equiv) and trifluoroacetic acid (6.9 mL, 89.4 mmol, 20 equiv) by following general procedure A. A pure material was obtained by column chromatography using gradient elution from DCM to 5% MeOH in DCM. ^1^H NMR (400 MHz, methanol-*d*_4_): δ 7.45–7.39 (m, 2H), 7.39–7.32 (m, 2H), 7.32–7.26 (m, 1H), 7.26–7.22 (m, 1H), 7.22–7.17 (m, 1H), 7.03–6.97 (m, 2H), 6.85 (ddd, *J* = 8.2, 2.6, 0.9 Hz, 1H), 6.81 (t, *J* = 2.0 Hz, 1H), 6.79–6.76 (m, 1H), 6.74 (dt, *J* = 7.6, 1.2 Hz, 1H), 5.07 (s, 2H), 3.76 (s, 3H), 3.64 (td, *J* = 5.7, 4.2 Hz, 1H), 3.14 (ddd, *J* = 8.4, 6.3, 4.2 Hz, 1H), 2.68 (dd, *J* = 13.6, 6.3 Hz, 1H), 2.49–2.41 (m, 3H), 1.45 (s, 3H), 1.42 (s, 3H), 0.92 (s, 9H), 0.05 (s, 3H), 0.03 (s, 3H). ^13^C NMR (101 MHz, methanol-*d*_4_): δ 161.3, 160.4, 150.1, 141.9, 138.8, 130.7, 130.3, 129.5, 128.9, 128.5, 122.7, 119.3, 116.8, 114.0, 113.4, 112.3, 75.8, 70.9, 57.6, 56.8, 55.7, 45.0, 40.1, 30.5, 28.6, 26.4, 19.0, −4.1, −4.4. HRMS-ESI (*m*/*z*) calcd for C_33_H_49_N_2_O_3_Si [M + H]^+^ 549.3512. Found 549.3539. [α]_D_^20^ = −3.4 (*c* 1.2, CHCl_3_).

#### Methyl 3-(((2*S*,3*R*)-1-(3-(Benzyloxy)phenyl)-3-hydroxy-4-((2-(3-methoxyphenyl)propan-2-yl)amino)butan-2-yl)carbamoyl)benzoate (**20**)

A solution of monomethyl isophthalate **19** (111 mg, 0.617 mmol, 1.2 equiv) in DCM (10 mL) was cooled to 0 °C. HOBT·H_2_O (118 mg, 0.617 mmol, 1.2 equiv) was added, followed by EDCl (109 μL, 0.617 mmol, 1.2 equiv) 20 min later. After stirring for 1 h, a solution of amine **17** (261 mg, 0.514 mmol, 1 equiv) and DIPEA (445 μL, 2.57 mmol, 5 equiv) in DCM (5 mL) was added. The reaction mixture was stirred at room temperature for 5 h. The solvent was evaporated under reduced pressure, and the residue was dissolved in EtOAc (30 mL) and water (30 mL). The aqueous phase was separated and extracted with EtOAc (2 × 15 mL). The combined organic phase was washed with brine (15 mL), dried over anhydrous Na_2_SO_4_, and evaporated under reduced pressure. Purification by column chromatography on silica gel using 50% EtOAc in hexanes afforded product **20** as a colorless oil (195 mg, 64% yield). ^1^H NMR (400 MHz, methanol-*d*_4_): δ 8.27 (td, *J* = 1.8, 0.6 Hz, 1H), 8.12 (dt, *J* = 7.8, 1.3 Hz, 1H), 7.81 (ddd, *J* = 7.8, 1.8, 1.2 Hz, 1H), 7.51 (td, *J* = 7.8, 0.6 Hz, 1H), 7.35–7.23 (m, 5H), 7.19–7.10 (m, 3H), 7.02–6.96 (m, 2H), 6.87 (dd, *J* = 2.6, 1.6 Hz, 1H), 6.81 (dt, *J* = 7.5, 1.2 Hz, 1H), 6.77 (ddd, *J* = 8.3, 2.6, 0.9 Hz, 1H), 6.71 (ddd, *J* = 8.2, 2.4, 1.1 Hz, 1H), 5.01–4.90 (m, 2H), 4.24 (ddd, *J* = 10.0, 7.4, 4.5 Hz, 1H), 3.89 (s, 3H), 3.71 (s, 3H), 3.69–3.63 (m, 1H), 3.10 (dd, *J* = 13.9, 4.5 Hz, 1H), 2.75 (dd, *J* = 13.9, 10.0 Hz, 1H), 2.53 (dd, *J* = 12.0, 3.4 Hz, 1H), 2.44 (dd, *J* = 12.1, 6.9 Hz, 1H), 1.47 (s, 6H). ^13^C NMR (101 MHz, methanol-*d*_4_): δ 169.2, 167.6, 161.2, 160.2, 149.5, 141.6, 138.8, 136.5, 133.2, 132.8, 131.7, 130.30, 130.27, 129.9, 129.4, 129.2, 128.8, 128.5, 123.0, 119.3, 116.8, 114.0, 113.2, 112.6, 73.4, 70.9, 57.0, 56.5, 55.6, 52.8, 46.9, 37.7, 30.0, 28.7. HRMS-ESI (*m*/*z*) calcd for C_36_H_41_N_2_O_6_ [M + H]^+^ 597.2965. Found 597.2980. [α]_D_^20^ = −41.3 (*c* 1.1, CHCl_3_).

#### Methyl 3-(((2*S*,3*R*)-1-(3-(Benzyloxy)phenyl)-3-((*tert*-butyldimethylsilyl)oxy)-4-((2-(3-methoxyphenyl)propan-2-yl)amino)butan-2-yl)carbamoyl)benzoate (**21**)

The title compound was obtained as a colorless oil (1.05 g, 84% yield) from compound **20** (1.05 g, 1.76 mmol, 1 equiv), TBSCl (1.33 g, 8.80 mmol, 5 equiv), and imidazole (599 mg, 8.80 mmol, 5 equiv) by following general procedure B. A pure material was obtained by column chromatography using gradient elution from hexanes to 30% EtOAc in hexanes. ^1^H NMR (400 MHz, chloroform-*d*): δ 9.65 (d, *J* = 6.9 Hz, 1H), 8.51 (td, *J* = 1.8, 0.6 Hz, 1H), 8.18 (dt, *J* = 7.7, 1.4 Hz, 1H), 8.13 (ddd, *J* = 7.8, 1.8, 1.2 Hz, 1H), 7.53 (t, *J* = 7.8 Hz, 1H), 7.43–7.39 (m, 2H), 7.38–7.33 (m, 2H), 7.32–7.23 (m, 2H), 7.21–7.13 (m, 1H), 7.01–6.94 (m, 2H), 6.84–6.75 (m, 4H), 5.02 (s, 2H), 4.52–4.42 (m, 1H), 3.89 (s, 3H), 3.74 (s, 3H), 3.64 (ddd, *J* = 4.6, 3.1, 1.3 Hz, 1H), 3.20 (dd, *J* = 13.7, 5.2 Hz, 1H), 2.69 (d, *J* = 12.1 Hz, 1H), 2.56 (ddd, *J* = 11.8, 4.7, 1.2 Hz, 1H), 2.40 (dd, *J* = 13.7, 10.3 Hz, 1H), 1.56 (s, 3H), 1.52 (s, 3H), 0.80 (s, 9H), −0.12 (s, 3H), −0.16 (s, 3H). ^13^C NMR (101 MHz, chloroform-*d*): δ 166.5, 166.3, 159.9, 159.0, 148.3, 139.9, 137.2, 135.9, 132.3, 132.0, 130.6, 129.7, 129.5, 128.9, 128.7, 128.0, 127.7, 127.6, 121.8, 118.3, 115.5, 113.2, 112.6, 111.5, 70.0, 68.7, 58.0, 55.7, 55.2, 52.3, 44.1, 38.6, 29.7, 29.3, 25.9, 18.1, −4.8, −5.1. HRMS-ESI (*m*/*z*) calcd for C_42_H_55_N_2_O_6_Si [M + H]^+^ 711.3829. Found 711.3833. [α]_D_^20^ = −39.3 (*c* 1.8, CHCl_3_).

#### Methyl 3-(((2*S*,3*R*)-3-((*tert*-Butyldimethylsilyl)oxy)-1-(3-hydroxyphenyl)-4-((2-(3-methoxyphenyl)propan-2-yl)amino)butan-2-yl)carbamoyl)benzoate (**22**)

To a solution of compound **21** (500 mg, 0.703 mmol, 1 equiv) in *i*-PrOH (20 mL), 10% Pd on carbon (150 mg, 0.141 mmol, 0.2 equiv) was added, and the reaction mixture was stirred under a hydrogen atmosphere (6 atm) for 16 h. The reaction mixture was filtered through a syringe filter (Teflon; 0.45 μm; washed with MeOH). The filtrate was evaporated under reduced pressure. Purification by column chromatography on silica gel using gradient elution from DCM to 2% MeOH in DCM afforded product **22** as a colorless oil (390 mg, 89% yield). ^1^H NMR (400 MHz, chloroform-*d*): δ 9.60 (d, *J* = 7.0 Hz, 1H), 8.50–8.47 (m, 1H), 8.18 (dt, *J* = 7.8, 1.4 Hz, 1H), 8.10 (ddd, *J* = 7.8, 1.8, 1.2 Hz, 1H), 7.53 (td, *J* = 7.8, 0.6 Hz, 1H), 7.29–7.22 (m, 1H), 7.09 (t, *J* = 7.8 Hz, 1H), 7.02–6.93 (m, 2H), 6.81–6.75 (m, 2H), 6.72–6.63 (m, 2H), 4.53–4.44 (m, 1H), 3.89 (s, 3H), 3.75 (s, 3H), 3.70 (ddd, *J* = 4.8, 3.2, 1.4 Hz, 1H), 3.12 (dd, *J* = 13.8, 5.7 Hz, 1H), 2.71 (dd, *J* = 11.9, 1.6 Hz, 1H), 2.60 (ddd, *J* = 11.9, 4.6, 1.1 Hz, 1H), 2.41 (dd, *J* = 13.8, 9.8 Hz, 1H), 1.56 (s, 3H), 1.51 (s, 3H), 0.80 (s, 9H), −0.09 (s, 3H), −0.13 (s, 3H). ^13^C NMR (101 MHz, chloroform-*d*): δ 166.7, 166.5, 159.8, 156.6, 148.2, 139.8, 135.7, 132.4, 132.1, 130.6, 129.7, 129.5, 128.9, 127.7, 121.2, 118.3, 116.0, 113.8, 112.6, 111.5, 68.9, 57.9, 55.8, 55.3, 52.3, 44.2, 38.3, 29.6, 29.2, 25.9, 18.1, −4.8, −5.1. HRMS-ESI (*m*/*z*) calcd for C_35_H_49_N_2_O_6_Si [M + H]^+^ 621.3360. Found 621.3367. [α]_D_^20^ = −40.4 (*c* 1.4, CHCl_3_).

#### Methyl 3-(((2*S*,3*R*)-3-((*tert*-Butyldimethylsilyl)oxy)-4-((2-(3-methoxyphenyl)propan-2-yl)amino)-1-(3-(4-((2-nitro-*N*-propylphenyl)sulfonamido)butoxy)phenyl)butan-2-yl)carbamoyl)benzoate (**25a**)

The title compound was obtained as a colorless oil (30 mg, 71% yield) from compound **22** (36 mg, 0.058 mmol, 1 equiv), alcohol **23a** (91 mg, 0.29 mmol, 5 equiv), DEAD (30 μL, 0.17 mmol, 3 equiv), PPh_3_ (46 mg, 0.17 mmol, 3 equiv), thiophenol (12 μL, 0.12 mmol, 2 equiv), and K_2_CO_3_ (32 mg, 0.23 mmol, 4 equiv), following general procedure C. ^1^H NMR (400 MHz, chloroform-*d*): δ 9.65 (d, *J* = 6.9 Hz, 1H), 8.50 (td, *J* = 1.8, 0.5 Hz, 1H), 8.18 (dt, *J* = 7.8, 1.4 Hz, 1H), 8.12 (ddd, *J* = 7.8, 1.9, 1.2 Hz, 1H), 7.53 (td, *J* = 7.7, 0.5 Hz, 1H), 7.30–7.21 (m, 1H), 7.14 (t, *J* = 8.0 Hz, 1H), 6.98 (ddd, *J* = 7.8, 1.8, 0.9 Hz, 1H), 6.95 (dd, *J* = 2.5, 1.7 Hz, 1H), 6.77 (ddd, *J* = 8.2, 2.5, 0.9 Hz, 1H), 6.75–6.69 (m, 3H), 4.47 (dtt, *J* = 9.3, 5.7, 3.1 Hz, 1H), 3.93 (t, *J* = 6.3 Hz, 2H), 3.89 (s, 3H), 3.74 (s, 3H), 3.68–3.64 (m, 1H), 3.18 (dd, *J* = 13.7, 5.2 Hz, 1H), 2.77–2.65 (m, 3H), 2.63–2.55 (m, 3H), 2.40 (dd, *J* = 13.7, 10.3 Hz, 1H), 1.87–1.73 (m, 2H), 1.67 (dtd, *J* = 9.4, 7.4, 5.3 Hz, 2H), 1.59–1.46 (m, 8H), 0.92 (t, *J* = 7.4 Hz, 3H), 0.79 (s, 9H), −0.12 (s, 3H), −0.16 (s, 3H). ^13^C NMR (101 MHz, CDCl_3_): δ 166.5, 166.3, 159.9, 159.3, 148.3, 139.8, 135.9, 132.3, 132.0, 130.5, 129.6, 129.5, 128.9, 127.7, 121.5, 118.3, 115.1, 112.9, 112.5, 111.5, 68.7, 67.8, 58.0, 55.7, 55.2, 52.3, 51.9, 49.6, 44.1, 38.6, 29.7, 29.3, 27.3, 26.6, 25.8, 23.1, 18.1, 11.9, −4.9, −5.18. HRMS-ESI (*m*/*z*) calcd for C_42_H_64_N_3_O_6_Si [M + H]^+^ 734.4564. Found 734.4561. [α]_D_^20^ = −36.0 (*c* 1.3, CHCl_3_).

#### Methyl 3-(((2*S*,3*R*)-3-((*tert*-Butyldimethylsilyl)oxy)-4-((2-(3-methoxyphenyl)propan-2-yl)amino)-1-(3-(4-((*N*-neopentyl-2-nitrophenyl)sulfonamido)butoxy)phenyl)butan-2-yl)carbamoyl)benzoate (**25b**)

The title compound was obtained as a yellowish oil (50 mg, 82% yield) from compound **22** (50 mg, 0.081 mmol, 1 equiv), alcohol **23b** (111 mg, 0.322 mmol, 4 equiv), DTBAD (56 mg, 0.24 mmol, 3 equiv), PPh_3_ (84 mg, 0.322 mmol, 4 equiv), thiophenol (33 μL, 0.321 mmol, 4 equiv), and K_2_CO_3_ (67 mg, 0.48 mmol, 6 equiv), following general procedure C. ^1^H NMR (400 MHz, chloroform-*d*): δ 9.57 (d, *J* = 6.9 Hz, 1H), 8.47 (t, *J* = 1.7 Hz, 1H), 8.16 (dt, *J* = 7.8, 1.4 Hz, 1H), 8.09 (dt, *J* = 7.9, 1.5 Hz, 1H), 7.51 (t, *J* = 7.8 Hz, 1H), 7.24 (t, *J* = 7.9 Hz, 1H), 7.12 (t, *J* = 8.0 Hz, 1H), 6.99–6.94 (m, 2H), 6.78–6.71 (m, 3H), 6.70–6.66 (m, 1H), 4.44 (dtd, *J* = 9.6, 6.0, 3.1 Hz, 1H), 3.92 (t, *J* = 6.1 Hz, 2H), 3.88 (s, 3H), 3.73 (s, 3H), 3.70 (d, *J* = 4.2 Hz, 1H), 3.14 (dd, *J* = 13.7, 5.6 Hz, 1H), 3.04–2.95 (m, 2H), 2.71 (dd, *J* = 11.9, 1.5 Hz, 1H), 2.66 (s, 2H), 2.59 (dd, *J* = 11.8, 4.4 Hz, 1H), 2.42 (dd, *J* = 13.7, 9.7 Hz, 1H), 2.03–1.91 (m, 2H), 1.79 (p, *J* = 6.4 Hz, 2H), 1.56 (s, 3H), 1.52 (s, 3H), 1.07 (s, 9H), 0.78 (s, 9H), −0.12 (s, 3H), −0.16 (s, 3H). ^13^C NMR (101 MHz, chloroform-*d*): δ 166.5, 166.3, 159.8, 159.0, 148.0, 139.8, 135.7, 132.3, 131.9, 130.5, 129.54, 129.46, 128.8, 127.7, 121.7, 118.3, 115.3, 112.7, 112.5, 111.5, 68.7, 67.3, 59.9, 57.8, 55.9, 55.2, 52.3, 49.5, 44.2, 38.4, 31.1, 29.5, 29.1, 27.9, 26.9, 25.8, 23.3, 18.0, −4.9, −5.1. HRMS-ESI (*m*/*z*) calcd for C_44_H_68_N_3_O_6_Si [M + H]^+^ 762.4877. Found 762.4885. [α]_D_^20^ = −35.9 (*c* 1.1, CHCl_3_).

#### Methyl 3-(((2*S*,3*R*)-3-((*tert*-Butyldimethylsilyl)oxy)-4-((2-(3-methoxyphenyl)propan-2-yl)amino)-1-(3-((5-(propylamino)pentyl)oxy)phenyl)butan-2-yl)carbamoyl)benzoate (**25c**)

The title compound was obtained as a yellowish oil (50 mg, 83% yield) from compound **22** (50 mg, 0.081 mmol, 1 equiv), alcohol **23c** (80 mg, 0.24 mmol, 3 equiv), DEAD (51 μL, 0.32 mmol, 4 equiv), PPh_3_ (106 mg, 0.403 mmol, 5 equiv), thiophenol (17 μL, 0.16 mmol, 2 equiv), and K_2_CO_3_ (44 mg, 0.32 mmol, 4 equiv), following general procedure C. ^1^H NMR (400 MHz, chloroform-*d*): δ 9.65 (d, *J* = 6.9 Hz, 1H), 8.50 (td, *J* = 1.8, 0.6 Hz, 1H), 8.17 (dt, *J* = 7.8, 1.4 Hz, 1H), 8.12 (ddd, *J* = 7.8, 1.9, 1.2 Hz, 1H), 7.53 (td, *J* = 7.8, 0.5 Hz, 1H), 7.25 (t, *J* = 8.0 Hz, 1H), 7.14 (t, *J* = 8.0 Hz, 1H), 6.97 (ddd, *J* = 7.7, 1.9, 0.9 Hz, 1H), 6.95 (dd, *J* = 2.5, 1.6 Hz, 1H), 6.77 (ddd, *J* = 8.1, 2.5, 0.9 Hz, 1H), 6.75–6.67 (m, 3H), 4.51–4.42 (m, 1H), 3.91 (t, *J* = 6.3 Hz, 2H), 3.89 (s, 3H), 3.74 (s, 3H), 3.66 (ddd, *J* = 4.6, 3.1, 1.4 Hz, 1H), 3.18 (dd, *J* = 13.7, 5.2 Hz, 1H), 2.77–2.67 (m, 1H), 2.63 (dd, *J* = 7.7, 6.6 Hz, 2H), 2.61–2.55 (m, 3H), 2.39 (dd, *J* = 13.7, 10.3 Hz, 1H), 1.83–1.71 (m, 2H), 1.61–1.42 (m, 12H), 0.91 (t, *J* = 7.4 Hz, 3H), 0.79 (s, 9H), −0.12 (s, 3H), −0.16 (s, 3H). ^13^C NMR (101 MHz, chloroform-*d*): δ 166.5, 166.3, 159.8, 159.4, 148.37, 139.8, 135.8, 132.3, 132.0, 130.5, 129.53, 129.45, 128.8, 127.7, 121.4, 118.3, 115.1, 112.8, 112.5, 111.5, 68.7, 67.9, 58.0, 55.7, 55.2, 52.3, 52.1, 50.0, 44.1, 38.6, 30.0, 29.6, 29.4, 29.3, 25.8, 24.0, 23.3, 18.1, 11.9, −4.9, −5.1. HRMS-ESI (*m*/*z*) calcd for C_43_H_66_N_3_O_6_Si [M + H]^+^ 748.4721. Found 748.4718. [α]_D_^20^ = −30.2 (*c* 1.0, CHCl_3_).

#### Methyl 3-(((2*S*,3*R*)-3-((*tert*-Butyldimethylsilyl)oxy)-4-((2-(3-methoxyphenyl)propan-2-yl)amino)-1-(3-(2-((2-nitro-*N*-propylphenyl)sulfonamido)ethoxy)phenyl)butan-2-yl)carbamoyl)benzoate (**25d**)

The title compound was obtained as a yellowish oil (31 mg, 50% yield) from compound **22** (55 mg, 0.089 mmol, 1 equiv), alcohol **23d** (77 mg, 0.27 mmol, 3 equiv), DEAD (28 μL, 0.18 mmol, 2 equiv), PPh_3_ (70 mg, 0.27 mmol, 3 equiv), thiophenol (18 μL, 0.18 mmol, 2 equiv), and K_2_CO_3_ (49 mg, 0.36 mmol, 4 equiv), following general procedure C. ^1^H NMR (400 MHz, chloroform-*d*): δ 9.64 (d, *J* = 6.9 Hz, 1H), 8.50 (dt, *J* = 1.8, 0.9 Hz, 1H), 8.18 (dt, *J* = 7.8, 1.4 Hz, 1H), 8.12 (ddd, *J* = 7.8, 1.8, 1.2 Hz, 1H), 7.53 (t, *J* = 7.8 Hz, 1H), 7.26 (t, *J* = 7.9 Hz, 1H), 7.15 (t, *J* = 7.7 Hz, 1H), 6.98 (ddd, *J* = 7.8, 1.8, 0.9 Hz, 1H), 6.96 (t, *J* = 2.1 Hz, 1H), 6.81–6.70 (m, 4H), 4.51–4.42 (m, 1H), 4.04 (t, *J* = 5.2 Hz, 2H), 3.89 (s, 3H), 3.74 (s, 3H), 3.69–3.63 (m, 1H), 3.18 (dd, *J* = 13.7, 5.3 Hz, 1H), 2.99 (dd, *J* = 5.8, 4.7 Hz, 2H), 2.72 (dd, *J* = 11.7, 1.4 Hz, 1H), 2.64 (dd, *J* = 7.8, 6.8 Hz, 2H), 2.59 (ddd, *J* = 11.8, 4.7, 1.2 Hz, 1H), 2.40 (dd, *J* = 13.7, 10.2 Hz, 1H), 1.58–1.51 (m, 8H), 0.93 (t, *J* = 7.4 Hz, 3H), 0.79 (s, 9H), −0.12 (s, 3H), −0.16 (s, 3H). ^13^C NMR (101 MHz, chloroform-*d*): δ 166.5, 166.3, 159.9, 159.2, 148.3, 139.9, 135.9, 132.3, 132.0, 130.6, 129.6, 129.5, 128.9, 127.7, 121.7, 118.3, 115.3, 112.7, 112.5, 111.5, 68.7, 67.3, 58.0, 55.7, 55.2, 52.3, 51.8, 48.9, 44.2, 38.6, 29.6, 29.3, 25.8, 23.3, 18.1, 11.9, −4.8, −5.1. HRMS-ESI (*m*/*z*) calcd for C_40_H_60_N_3_O_6_Si [M + H]^+^ 706.4251. Found 706.4254. [α]_D_^20^ = −40.8 (*c* 1.2, CHCl_3_).

#### (*S*)-4-((*R*)-1-Hydroxy-2-((2-(3-methoxyphenyl)propan-2-yl)amino)ethyl)-12-propyl-7-oxa-3,12-diaza-1,6(1,3)-dibenzenacyclotridecaphane-2,13-dione (**7a**)

The hydrolysis of methyl benzoate **25a** (32 mg 0.044 mmol, 1 equiv) was performed using 1 M aqueous NaOH solution (89 μL, 0.89 mmol, 2 equiv), following procedure D. The crude product was used in the next step without purification. The macrolactamization was performed using HBTU (25 mg, 0.65 mmol, 1.5 equiv), following procedure E. The crude product was used in the next step without purification. The desilylation was performed using NH_4_F (47 mg, 1.3 mmol, 30 equiv), following procedure F. Full conversion was achieved after stirring at 40 °C overnight. Purification by column chromatography on silica gel using gradient elution from DCM to 10% MeOH in DCM afforded product **7a** as an amorphous solid (17 mg, 68% yield). ^1^H NMR (400 MHz, methanol-*d*_4_) for a mixture of rotamers (0.72:0.20:0.08): δ 7.75–7.35 (m, 4H), 7.26–6.92 (m, 4H), 6.91–3.63 (m, 4H), 4.50–4.07 (m, 2H), 3.91–3.64 (m, 5H), 3.63–3.36 (m, 2H), 3.27–2.96 (m, 3H), 2.91–2.37 (m, 3H), 2.21–1.39 (m, 12H), 1.01 (t, *J* = 7.4 Hz, 2.15H), 0.73 (t, *J* = 7.3 Hz, 0.25H), 0.60 (t, *J* = 7.4 Hz, 0.6H). ^13^C NMR (101 MHz, methanol-*d*_4_) for a major rotamer: δ 173.0, 168.8, 161.3, 159.1, 149.2, 141.8, 138.4, 135.5, 130.8, 130.5, 130.34, 130.31, 129.6, 124.7, 122.7, 119.3, 118.1, 113.7, 113.2, 112.7, 74.0, 67.8, 57.3, 56.3, 55.6, 50.1, 47.9, 47.3, 37.4, 29.8, 28.6, 26.7, 26.6, 21.8, 11.7. HRMS-ESI (*m*/*z*) calcd for C_35_H_46_N_3_O_5_ [M + H]^+^ 588.3437. Found 588.3453. [α]_D_^20^ = 15.4 (*c* 1.7, CHCl_3_).

#### (*S*)-4-((*R*)-1-Hydroxy-2-((2-(3-methoxyphenyl)propan-2-yl)amino)ethyl)-12-propyl-7-oxa-3,12-diaza-1,6(1,3)-dibenzenacyclotridecaphane-2,13-dione (**7e**)

The hydrolysis of methyl benzoate **25b** (50 mg 0.066 mmol, 1 equiv) was performed using 1 M aqueous NaOH solution (131 μL, 0.131 mmol, 2 equiv), following procedure D. The crude product was used in the next step without purification. The macrolactamization was performed using HBTU (37 mg, 0.98 mmol, 1.5 equiv), following procedure E. The crude product was used in the next step without purification. The desilylation was performed using NH_4_F (73 mg, 2.0 mmol, 30 equiv), following procedure F. Full conversion was achieved after stirring at room temperature for 64 h. The crude product was purified by column chromatography on silica gel using gradient elution from DCM to 10% MeOH in DCM. Additional purification by preparative reverse phase HPLC using gradient elution from 10 to 95% MeCN in 0.1% aqueous HCOOH solution afforded product **7e** as an amorphous solid (15 mg, 37% yield). ^1^H NMR (400 MHz, methanol-*d*_4_) for a major rotamer: δ 7.68 (dt, *J* = 7.5, 1.7 Hz, 1H), 7.60 (t, *J* = 1.6 Hz, 1H), 7.55–7.50 (m, 1H), 7.50–7.46 (m, 1H), 7.17 (t, *J* = 8.2 Hz, 1H), 7.11 (t, *J* = 7.8 Hz, 1H), 7.01–6.96 (m, 2H), 6.82 (d, *J* = 7.7 Hz, 1H), 6.74–6.70 (m, 1H), 6.64–6.60 (m, 1H), 6.60–6.57 (m, 1H), 4.10 (ddd, *J* = 11.5, 8.1, 3.6 Hz, 1H), 3.77–3.69 (m, 6H), 3.54–3.45 (m, 1H), 3.40–3.32 (m, 3H), 3.19 (dd, *J* = 13.5, 3.6 Hz, 1H), 2.63 (dd, *J* = 13.4, 11.2 Hz, 1H), 2.55 (dd, *J* = 11.9, 3.3 Hz, 1H), 2.41 (dd, *J* = 11.9, 7.5 Hz, 1H), 1.64–1.48 (m, 4H), 1.46 (s, 3H), 1.45 (s, 3H), 1.06 (s, 9H). ^13^C NMR (101 MHz, methanol-*d*_4_) for a major rotamer: δ 174.4, 168.8, 161.2, 159.2, 149.9, 141.8, 138.7, 136.0, 130.7, 130.5, 130.3, 130.2, 129.4, 125.4, 122.7, 119.4, 117.9, 113.3, 113.2, 112.5, 74.1, 67.8, 56.9, 56.8, 56.1, 55.7, 52.2, 47.5, 37.7, 35.4, 30.2, 29.0, 28.9, 26.8, 26.7. HRMS-ESI (*m*/*z*) calcd for C_37_H_50_N_3_O_5_ [M + H]^+^ 616.3750. Found 616.3769. [α]_D_^20^ = −2.1 (*c* 0.9, CHCl_3_).

#### (*S*)-4-((*R*)-1-Hydroxy-2-((2-(3-methoxyphenyl)propan-2-yl)amino)ethyl)-13-propyl-7-oxa-3,13-diaza-1,6(1,3)-dibenzenacyclotetradecaphane-2,14-dione (**7g**)

The hydrolysis of methyl benzoate **25c** (50 mg 0.067 mmol, 1 equiv) was performed using 1 M aqueous NaOH solution (134 μL, 0.134 mmol, 2 equiv), following procedure D. The crude product was used in the next step without purification. The macrolactamization was performed using HBTU (38 mg, 0.10 mmol, 1.5 equiv), following procedure E. The crude product was used in the next step without purification. The desilylation was performed using NH_4_F (50 mg, 1.3 mmol, 20 equiv), following procedure F. Full conversion was achieved after stirring at room temperature for 120 h. Purification by column chromatography on silica gel using gradient elution from DCM to 10% MeOH in DCM afforded product **7g** as an amorphous solid (9 mg, 22% yield). ^1^H NMR (400 MHz, methanol-*d*_4_) for a mixture of rotamers (0.8:0.2): δ 7.60 (d, *J* = 6.2 Hz, 1H), 7.49–7.37 (m, 3H), 7.19 (t, *J* = 7.9 Hz, 1H), 7.07 (t, *J* = 7.8 Hz, 1H), 7.03–6.96 (m, 3H), 6.80 (d, *J* = 7.6 Hz, 1H), 6.77–6.69 (m, 1H), 6.65 (d, *J* = 8.0 Hz, 1H), 4.53–4.37 (m, 2H), 4.17–4.08 (m, 1H), 3.75 (s, 3H), 3.68–3.52 (m, 3H), 3.52–3.40 (m, 1H), 3.29–3.22 (m, 1H), 2.58 (t, *J* = 12.5 Hz, 1H), 2.51 (dd, *J* = 11.9, 3.3 Hz, 1H), 2.42 (dd, *J* = 12.0, 7.3 Hz, 1H), 1.80 (ddd, *J* = 29.3, 14.1, 6.6 Hz, 2H), 1.47 (s, 6H), 1.04 (t, *J* = 7.4 Hz, 2.4H), 0.80 (br s, 0.6H). ^13^C NMR (101 MHz, methanol-*d*_4_) for a major rotamer: δ 173.6, 169.5, 161.2, 158.4, 149.8, 142.3, 137.3, 135.5, 131.2, 130.9, 130.4, 130.3, 125.4, 122.8, 120.5, 119.3, 113.3, 112.4, 74.7, 63.9, 56.9, 56.2, 55.6, 49.0, 47.6, 47.4, 38.5, 30.3, 28.5, 21.7, 11.7. HRMS-ESI (*m*/*z*) calcd for C_33_H_42_N_3_O_5_ [M + H]^+^ 560.3124. Found 560.3136. [α]_D_^20^ = −10.1 (*c* 1.0, CHCl_3_).

#### (*S*)-4-((*R*)-1-Hydroxy-2-((2-(3-methoxyphenyl)propan-2-yl)amino)ethyl)-13-propyl-7-oxa-3,13-diaza-1,6(1,3)-dibenzenacyclotetradecaphane-2,14-dione (**7h**)

The hydrolysis of methyl benzoate **25d** (57 mg 0.081 mmol, 1 equiv) was performed using 1 M aqueous NaOH solution (162 μL, 0.162 mmol, 2 equiv), following procedure D. The crude product was used in the next step without purification. The macrolactamization was performed using HBTU (46 mg, 0.121 mmol, 1.5 equiv), following procedure E. The crude product was used in the next step without purification. The desilylation was performed using NH_4_F (76 mg, 2.0 mmol, 25 equiv), by following procedure F. Full conversion was achieved after stirring at room temperature for 100 h. The crude product was purified by column chromatography on silica gel using gradient elution from DCM to 10% MeOH in DCM. Additional purification by preparative reverse phase HPLC using gradient elution from 10 to 95% MeCN in 0.1% aqueous HCOOH solution afforded product **7h** as an amorphous solid (9 mg, 19% yield). ^1^H NMR (400 MHz, methanol-*d*_4_) for a mixture of rotamers (0.87:0.13): δ 7.78–7.25 (m, 5H), 7.25–6.95 (m, 4H), 6.93–6.85 (m, 1H), 6.84–6.76 (m, 1H), 6.76–6.52 (m, 1H), 4.64–4.10 (m, 2H), 4.10–3.89 (m, 1H), 3.88–3.69 (m, 4H), 3.69–3.53 (m, 2H), 3.53–3.36 (m, 2H), 3.28–3.20 (m, 1H), 2.96–2.66 (m, 2H), 2.58 (t, *J* = 12.5 Hz, 1H), 1.90–1.51 (m, 8H), 1.04 (t, *J* = 7.4 Hz, 2.6H), 0.80 (br s, 0.4H). ^13^C NMR (101 MHz, methanol-*d*_4_) for a major rotamer: δ 173.6, 169.5, 161.2, 158.4, 149.8, 142.3, 137.3, 135.5, 131.2, 130.9, 130.4, 130.3, 125.4, 122.8, 120.5, 119.3, 113.3, 112.4, 74.7, 63.9, 56.9, 56.3, 55.6, 49.0, 47.6, 47.4, 38.5, 30.3, 28.5, 21.7, 11.7. HRMS-ESI (*m*/*z*) calcd for C_33_H_42_N_3_O_5_ [M + H]^+^ 560.3124. Found 560.3136. [α]_D_^20^ = 3.4 (*c* 0.8, CHCl_3_).

#### Methyl 3-((4-(Benzyloxy)butyl)carbamoyl)benzoate (**27a**)

The title compound was obtained as a colorless oil (117 mg, 68% yield) from acid **19** (90 mg, 0.50 mmol, 1 equiv), HBTU (190 mg, 0.50 mmol, 1 equiv), DIPEA (87 μL, 0.50 mmol, 1 equiv), and amine **26a** (90 mg, 0.50 mmol, 1 equiv), following general procedure G. A pure material was obtained by column chromatography on silica gel using gradient elution from 10% EtOAc to 30% EtOAc in hexanes. ^1^H NMR (400 MHz, chloroform-*d*): δ 8.36 (td, *J* = 1.8, 0.6 Hz, 1H), 8.13 (dt, *J* = 7.8, 1.4 Hz, 1H), 7.97 (ddd, *J* = 7.8, 1.9, 1.2 Hz, 1H), 7.47 (td, *J* = 7.8, 0.6 Hz, 1H), 7.35–7.22 (m, 5H), 6.71–6.62 (m, 1H), 4.53 (s, 2H), 3.92 (s, 3H), 3.57–3.46 (m, 4H), 1.80–1.70 (m, 4H). ^13^C NMR (101 MHz, chloroform-*d*) δ 166.6, 166.5, 138.3, 135.2, 132.3, 131.9, 130.5, 128.9, 128.5, 127.8, 127.6, 73.2, 70.1, 52.5, 40.1, 27.3, 26.6. HRMS-ESI (*m*/*z*) calcd for C_20_H_24_NO_4_ [M + H]^+^ 342.1705. Found 342.1721.

#### Methyl 3-((4-(Benzyloxy)butyl)(methyl)carbamoyl)benzoate (**27b**)

The title compound was obtained as a colorless oil (120 mg, 45% yield) from acid **19** (149 mg, 0.825 mmol, 1.1 equiv), HBTU (313 mg, 0.825 mmol, 1.1 equiv), DIPEA (143 μL, 0.825 mmol, 1.1 equiv), and crude amine **26b** (145 mg, 0.750 mmol, 1 equiv), following general procedure G. A pure material was obtained by column chromatography on silica gel using gradient elution from 10% EtOAc to 30% EtOAc in hexanes. ^1^H NMR (400 MHz, chloroform-*d*) for 2 rotamers: δ 8.11–8.02 (m, 2H), 7.58 (t, *J* = 6.6 Hz, 1H), 7.47 (q, *J* = 8.1 Hz, 1H), 7.39–7.23 (m, 5H), 4.53 (s, 1H), 4.40 (s, 1H), 3.92 (s, 3H), 3.56 (q, *J* = 6.0, 5.6 Hz, 2H), 3.35 (t, *J* = 6.1 Hz, 1H), 3.25 (t, *J* = 7.5 Hz, 1H), 3.08 (s, 1.5H), 2.93 (s, 1.5H), 1.83–1.64 (m, 3H), 1.49–1.40 (m, 1H). ^13^C NMR (101 MHz, chloroform-*d*) for 2 rotamers: δ 170.9, 170.4, 166.5, 138.7, 138.4, 137.2, 131.5, 131.3, 130.5, 130.4, 128.8, 128.5, 128.1, 128.0, 127.8, 127.7, 73.1, 70.1, 69.6, 52.4, 51.3, 47.5, 37.6, 32.9, 27.6, 27.2, 26.8, 25.4, 24.0. HRMS-ESI (*m*/*z*) calcd for C_21_H_26_NO_4_ [M + H]^+^ 356.1862. Found 356.1871.

#### Methyl 3-((4-(Benzyloxy)butyl)(3,3,3-trifluoropropyl)carbamoyl)benzoate (**27c**)

The title compound was obtained as a yellow oil (253 mg, 95% yield) from acid **19** (110 mg, 0.611 mmol, 1 equiv), HBTU (232 mg, 0.611 mmol, 1 equiv), DIPEA (317 μL, 1.83 mmol, 3 equiv), and amine **26c** (168 mg, 0.611 mmol, 1 equiv), following general procedure G. A pure material was obtained by column chromatography on silica gel using 2% MeOH in DCM. ^1^H NMR (400 MHz, chloroform-*d*) for 2 rotamers: δ 8.09 (dt, *J* = 7.7, 1.5 Hz, 1H), 8.03 (t, *J* = 1.7 Hz, 1H), 7.55 (dt, *J* = 7.6, 1.5 Hz, 1H), 7.52–7.42 (m, 1H), 7.37–7.31 (m, 2H), 7.30–7.21 (m, 3H), 4.60–4.33 (m, 2H), 3.92 (s, 3H), 3.78–3.42 (m, 3H), 3.39–3.18 (m, 3H), 2.66–2.16 (m, 2H), 1.75–1.34 (m, 4H). ^13^C NMR (101 MHz, chloroform-*d*) for a major rotamer: δ 171.0, 166.3, 138.4, 136.7, 131.1, 130.8, 130.6, 128.9, 128.5, 127.82, 127.77, 127.71, 73.1, 69.5, 52.5, 50.2, 39.7, 31.9 (q, *J* = 29.2 Hz), 26.78, 26.02. HRMS-ESI (*m*/*z*) calcd for C_23_H_27_NO_4_F_3_ [M + H]^+^ 438.1906. Found 438.1901.

#### Methyl 3-((4-(Benzyloxy)butyl)(2-(dimethylamino)ethyl)carbamoyl)benzoate (**27d**)

The title compound was obtained as a yellow oil (2.1 g, 83% yield) from acid **19** (1.10 g, 6.11 mmol, 1 equiv), HBTU (2.32 mg, 6.11 mmol, 1 equiv), DIPEA (1.06 mL, 6.11 mmol, 1 equiv), and crude amine **26d** (1.8 g, 7.3 mmol, 1.2 equiv), following general procedure G. A pure material was obtained by column chromatography on silica gel using 2% MeOH in DCM. ^1^H NMR (400 MHz, methanol-*d*_4_) for 2 rotamers: δ 8.14–7.95 (m, 2H), 7.68–7.39 (m, 2H), 7.38–7.16 (m, 5H), 4.57–4.30 (m, 2H), 3.70–3.51 (m, 3H), 3.41–3.21 (m, 6H), 2.68–2.38 (m, 2H), 2.36–1.96 (m, 6H), 1.87–1.32 (m, 4H). ^13^C NMR (101 MHz, methanol-*d*_4_) for 2 rotamers: δ 173.0, 167.7, 139.81, 139.70, 139.59, 138.3, 132.2, 131.8, 131.5, 131.4, 130.2, 129.38, 129.26, 128.89, 128.71, 128.65, 128.58, 73.96, 73.85, 71.0, 58.3, 57.2, 53.9, 50.7, 48.1, 46.6, 45.74, 45.58, 44.0, 28.1, 27.6, 26.7, 25.5. HRMS-ESI (*m*/*z*) calcd for C_24_H_33_N_2_O_4_ [M + H]^+^ 413.2440. Found 413.2445.

#### *N*^1^-(4-(Benzyloxy)butyl)-*N*^3^-((2*S*,3*R*)-1-(3-(benzyloxy)phenyl)-3-((*tert*-butyldimethylsilyl)oxy)-4-((2-(3-methoxyphenyl)propan-2-yl)amino)butan-2-yl)isophthalamide (**28a**)

The hydrolysis of methyl benzoate was performed using compound **27a** (205 mg, 0.601 mmol, 1 equiv) and 1 M NaOH solution (1.80 mL, 1.80 mmol, 3 equiv), following general procedure D. Full conversion was achieved after stirring at 50 °C for 4 h. Crude acid was used in the next step without further purification. The title compound was obtained as a colorless oil (190 mg, 37% yield over 2 steps) from amine **18** (330 mg, 0.601 mmol, 1 equiv), HBTU (342 mg, 0.902 mmol, 1.5 equiv), DIPEA (208 μL, 1.20 mmol, 2 equiv), and crude acid from the previous step, following general procedure G. A pure material was obtained by column chromatography on silica gel using gradient elution from 10% EtOAc in hexanes to 100% EtOAc. ^1^H NMR (400 MHz, methanol-*d*_4_): δ 8.22 (td, *J* = 1.9, 0.6 Hz, 1H), 7.93 (ddd, *J* = 7.8, 1.8, 1.1 Hz, 1H), 7.87 (ddd, *J* = 7.8, 1.9, 1.1 Hz, 1H), 7.53 (td, *J* = 7.7, 0.5 Hz, 1H), 7.39–7.33 (m, 2H), 7.33–7.29 (m, 5H), 7.29–7.27 (m, 1H), 7.27–7.24 (m, 1H), 7.24–7.18 (m, 2H), 7.16 (t, *J* = 7.8 Hz, 1H), 7.01–6.95 (m, 2H), 6.84–6.82 (m, 1H), 6.82–6.80 (m, 1H), 6.80–6.75 (m, 2H), 5.01 (s, 2H), 4.48 (s, 2H), 4.43 (td, *J* = 7.6, 4.4 Hz, 1H), 3.72 (td, *J* = 4.4, 2.9 Hz, 1H), 3.70 (s, 3H), 3.55–3.50 (m, 2H), 3.43–3.37 (m, 2H), 2.86 (dd, *J* = 13.7, 7.7 Hz, 1H), 2.68 (dd, *J* = 13.7, 7.6 Hz, 1H), 2.62 (dd, *J* = 12.1, 2.9 Hz, 1H), 2.53 (dd, *J* = 12.0, 4.4 Hz, 1H), 1.74–1.62 (m, 4H), 1.48 (s, 3H), 1.47 (s, 3H), 0.86 (s, 9H), −0.02 (s, 3H), −0.05 (s, 3H). ^13^C NMR (101 MHz, methanol-*d*_4_): δ 169.3, 169.0, 161.3, 160.3, 149.6, 141.2, 139.8, 138.8, 136.63, 136.58, 131.0, 130.9, 130.5, 130.4, 129.9, 129.5, 129.4, 128.9, 128.8, 128.6, 128.4, 127.2, 122.7, 119.4, 116.5, 114.3, 113.5, 112.5, 73.9, 72.1, 71.0, 70.8, 58.2, 56.7, 55.6, 45.8, 40.9, 38.7, 29.9, 29.2, 28.2, 27.3, 26.4, 18.9, −4.5, −4.6. HRMS-ESI (*m*/*z*) calcd for C_52_H_68_N_3_O_6_Si [M + H]^+^ 858.4877. Found 858.4862. [α]_D_^20^ = −40.7 (*c* 1.1, CHCl_3_).

#### *N*^1^-(4-(Benzyloxy)butyl)-*N*^3^-((2*S*,3*R*)-1-(3-(benzyloxy)phenyl)-3-((*tert*-butyldimethylsilyl)oxy)-4-((2-(3-methoxyphenyl)propan-2-yl)amino)butan-2-yl)-*N*^1^-methylisophthalamide (**28b**)

The hydrolysis of methyl benzoate was performed using compound **27b** (45 mg, 0.127 mmol, 1 equiv) and 1 M NaOH solution (254 μL, 0.254 mmol, 2 equiv), following general procedure D. Full conversion was achieved after stirring at 50 °C for 4 h. Crude acid was used in the next step without further purification. The title compound was obtained as a colorless oil (68 mg, 61% yield over 2 steps) from amine **18** (105 mg, 0.191 mmol, 1.5 equiv), HBTU (73 mg, 0.19 mmol, 1.5 equiv), DIPEA (66 μL, 0.38 mmol, 3 equiv), and crude acid from the previous step, following general procedure G. A pure material was obtained by column chromatography on silica gel using gradient elution from 10% EtOAc in hexanes to 100% EtOAc. ^1^H NMR (400 MHz, chloroform-*d*): δ 9.60 (d, *J* = 6.9 Hz, 1H), 7.96–7.86 (m, 2H), 7.57–7.48 (m, 1H), 7.48–7.43 (m, 1H), 7.43–7.38 (m, 2H), 7.38–7.27 (m, 8H), 7.26–7.23 (m, 1H), 7.21–7.11 (m, 1H), 7.01–6.91 (m, 2H), 6.84–6.74 (m, 4H), 5.02 (s, 2H), 4.56–4.31 (m, 3H), 3.75 (s, 3H), 3.66–3.60 (m, 1H), 3.59–3.47 (m, 2H), 3.35 (t, *J* = 6.1 Hz, 1H), 3.31–3.15 (m, 2H), 3.05 (s, 1.5H), 2.92 (s, 1.5H), 2.68 (d, *J* = 11.8 Hz, 1H), 2.54 (dd, *J* = 11.8, 4.6 Hz, 1H), 2.39 (dd, *J* = 13.7, 10.3 Hz, 1H), 1.80–1.57 (m, 3H), 1.52 (s, 3H), 1.50 (s, 3H), 1.46–1.38 (m, 1H), 0.79 (s, 9H), −0.13 (s, 3H), −0.17 (s, 3H). ^13^C NMR (101 MHz, chloroform-*d*): δ 171.3, 170.6, 166.4, 166.3, 159.9, 159.0, 148.2, 139.9, 138.7, 138.5, 137.34, 137.25, 137.17, 135.5, 129.7, 129.5, 128.8, 128.7, 128.5, 128.0, 127.8, 127.7, 127.6, 125.7, 125.5, 121.8, 118.3, 115.5, 113.2, 112.6, 111.4, 73.1, 70.0, 69.6, 68.6, 58.0, 55.7, 55.3, 51.3, 47.4, 44.1, 38.6, 37.6, 32.8, 29.8, 29.4, 27.3, 26.8, 25.9, 25.4, 23.9, −4.8, −5.1. HRMS-ESI (*m*/*z*) calcd for C_53_H_70_N_3_O_6_Si [M + H]^+^ 872.5034. Found 872.5039. [α]_D_^20^ = −36.2 (*c* 1.2, CHCl_3_).

#### *N*^1^-(4-(Benzyloxy)butyl)-*N*^3^-((2*S*,3*R*)-1-(3-(benzyloxy)phenyl)-3-((*tert*-butyldimethylsilyl)oxy)-4-((2-(3-methoxyphenyl)propan-2-yl)amino)butan-2-yl)-*N*^1^-(3,3,3-trifluoropropyl)isophthalamide (**28c**)

The hydrolysis of methyl benzoate was performed using compound **27c** (190 mg, 0.434 mmol, 1 equiv) and 1 M NaOH solution (2.61 mL, 2.61 mmol, 6 equiv), following general procedure D. Full conversion was achieved after stirring at room temperature for 4 h. Crude acid was used in the next step without further purification. The title compound was obtained as a colorless oil (240 mg, 58% yield over 2 steps) from amine **18** (239 mg, 0.435 mmol, 1 equiv), HBTU (165 mg, 0.435 mmol, 1 equiv), DIPEA (150 μL, 0.869 mmol, 2 equiv), and crude acid from the previous step, following general procedure G. A pure material was obtained by column chromatography on silica gel using gradient elution from 10% EtOAc in hexanes to 100% EtOAc. ^1^H NMR (400 MHz, chloroform-*d*): δ 9.63 (d, *J* = 6.8 Hz, 1H), 7.94–7.87 (m, 2H), 7.51–7.43 (m, 2H), 7.43–7.39 (m, 2H), 7.39–7.35 (m, 2H), 7.35–7.30 (m, 3H), 7.30–7.24 (m, 4H), 7.20–7.14 (m, 1H), 7.00–6.91 (m, 2H), 6.84–6.74 (m, 4H), 5.02 (s, 2H), 4.55–4.33 (m, 3H), 3.75 (s, 3H), 3.70–3.42 (m, 4H), 3.40–3.24 (m, 3H), 3.21 (dd, *J* = 13.7, 5.2 Hz, 1H), 2.68 (d, *J* = 12.1 Hz, 1H), 2.62–2.16 (m, 4H), 1.66–1.58 (m, 2H), 1.52 (s, 3H), 1.50 (s, 3H), 1.47–1.36 (m, 2H), 0.79 (s, 9H), −0.13 (s, 3H), −0.16 (s, 3H). ^13^C NMR (101 MHz, chloroform-*d*): δ 171.3, 166.2, 159.9, 159.0, 148.2, 139.8, 138.4, 137.2, 136.8, 135.8, 129.7, 129.5, 129.2, 128.9, 128.7, 128.5, 128.0, 127.7, 127.6, 126.7 (q, *J* = 255.1 Hz), 121.8, 118.2, 115.5, 113.1, 112.6, 111.4, 73.1, 70.0, 69.5, 68.6, 58.0, 55.7, 55.2, 50.2, 44.1, 39.5, 38.5, 31.9 (q, *J* = 27.3 Hz), 29.8, 29.4, 26.8, 26.0, 25.8, 18.1, −4.9, −5.1. HRMS-ESI (*m*/*z*) calcd for C_55_H_71_N_3_O_6_F_3_Si [M + H]^+^ 954.5064. Found 954.5062. [α]_D_^20^ = −29.8 (*c* 1.1, CHCl_3_).

#### *N*^1^-(4-(Benzyloxy)butyl)-*N*^3^-((2*S*,3*R*)-1-(3-(benzyloxy)phenyl)-3-((*tert*-butyldimethylsilyl)oxy)-4-((2-(3-methoxyphenyl)propan-2-yl)amino)butan-2-yl)-*N*^1^-(2-(dimethylamino)ethyl)isophthalamide (**28d**)

The hydrolysis of methyl benzoate was performed using compound **27d** (250 mg, 0.606 mmol, 1 equiv) and 1 M NaOH solution (1.21 mL, 1.21 mmol, 2 equiv), following general procedure D. Full conversion was achieved after stirring at 60 °C for 2 h. Crude acid was used in the next step without further purification. The title compound was obtained as a colorless oil (305 mg, 55% yield over 2 steps) from amine **18** (331 mg, 0.603 mmol, 1 equiv), HBTU (228 mg, 0.602 mmol, 1 equiv), DIPEA (208 μL, 1.20 mmol, 2 equiv), and crude acid from previous step, by following general procedure G. A pure material was obtained by column chromatography on silica gel using gradient elution from 10% EtOAc in hexanes to 100% EtOAc. ^1^H NMR (400 MHz, methanol-*d*_4_) for 2 rotamers: δ 7.87–7.81 (m, 1H), 7.77 (d, *J* = 8.1 Hz, 1H), 7.60–7.45 (m, 2H), 7.39–7.33 (m, 2H), 7.33–7.26 (m, 5H), 7.26–7.18 (m, 4H), 7.18–7.11 (m, 1H), 7.02–6.94 (m, 2H), 6.84–6.74 (m, 4H), 5.06–4.93 (m, 2H), 4.58–4.46 (m, 1H), 4.42 (td, *J* = 7.7, 4.3 Hz, 1H), 4.33 (s, 1H), 3.76–3.66 (m, 4H), 3.62 (t, *J* = 7.4 Hz, 1H), 3.53 (q, *J* = 7.8, 6.8 Hz, 2H), 3.35–3.32 (m, 1H), 3.29–3.22 (m, 2H), 2.86 (dd, *J* = 13.8, 7.6 Hz, 1H), 2.81 (s, 3H), 2.66 (dd, *J* = 13.7, 7.7 Hz, 1H), 2.62–2.55 (m, 2H), 2.55–2.48 (m, 1H), 2.29 (s, 3H), 1.96 (s, 3H), 1.80–1.72 (m, 1H), 1.72–1.64 (m, 1H), 1.64–1.54 (m, 1H), 1.52–1.42 (m, 6H), 1.41–1.32 (m, 1H), 0.84 (s, 9H), −0.05 (s, 3H), −0.07 (s, 3H). ^13^C NMR (101 MHz, methanol-*d*_4_) for 2 rotamers: δ 173.1, 168.5, 161.3, 160.2, 155.6, 149.5, 141.2, 139.6, 138.74, 138.71, 138.35, 138.28, 136.3, 130.63, 130.57, 130.47, 130.2, 129.5, 129.4, 129.2, 128.9, 128.8, 128.7, 128.4, 126.2, 122.7, 119.4, 116.6, 114.4, 113.6, 112.5, 74.0, 73.9, 72.0, 71.0, 70.8, 70.6, 58.3, 58.2, 57.3, 56.7, 55.7, 50.2, 46.7, 45.8, 45.6, 44.1, 39.0, 38.7, 30.4, 30.3, 29.3, 29.2, 28.1, 27.6, 26.7, 26.5, 26.4, 25.6, 18.9, −4.5, −4.6. HRMS-ESI (*m*/*z*) calcd for C_56_H_77_N_4_O_6_Si [M + H]^+^ 929.5612. Found 929.5617. [α]_D_^20^ = −44.9 (*c* 1.0, CHCl_3_).

#### *N*^1^-((2*S*,3*R*)-3-((*tert*-Butyldimethylsilyl)oxy)-1-(3-hydroxyphenyl)-4-((2-(3-methoxyphenyl)propan-2-yl)amino)butan-2-yl)-*N*^3^-(4-hydroxybutyl)isophthalamide (**29a**)

The title compound was obtained as a colorless oil (52 mg, 73% yield) from compound **28a** (90 mg, 0.10 mmol, 1 equiv) and 10% palladium on carbon (22 mg, 0.021 mmol, 0.2 equiv), following general procedure H. ^1^H NMR (400 MHz, methanol-*d*_4_): δ 8.22 (td, *J* = 1.8, 0.5 Hz, 1H), 7.95 (ddd, *J* = 7.8, 1.8, 1.1 Hz, 1H), 7.87 (ddd, *J* = 7.7, 1.9, 1.1 Hz, 1H), 7.54 (td, *J* = 7.8, 0.6 Hz, 1H), 7.21 (t, *J* = 8.0 Hz, 1H), 7.09–7.03 (m, 1H), 6.99 (ddd, *J* = 7.7, 1.8, 0.9 Hz, 1H), 6.97 (t, *J* = 2.1 Hz, 1H), 6.77 (ddd, *J* = 8.2, 2.5, 0.8 Hz, 1H), 6.69–6.64 (m, 2H), 6.61 (ddd, *J* = 8.1, 2.4, 1.1 Hz, 1H), 4.42 (td, *J* = 7.6, 4.4 Hz, 1H), 3.78 (td, *J* = 4.3, 2.9 Hz, 1H), 3.71 (s, 3H), 3.60 (t, *J* = 6.3 Hz, 2H), 3.41 (t, *J* = 6.9 Hz, 2H), 2.85 (dd, *J* = 13.6, 7.5 Hz, 1H), 2.71–2.61 (m, 2H), 2.58 (dd, *J* = 12.0, 4.4 Hz, 1H), 1.75–1.65 (m, 2H), 1.65–1.56 (m, 2H), 1.50 (s, 3H), 1.49 (s, 3H), 0.86 (s, 9H), −0.01 (s, 3H), −0.04 (s, 3H). ^13^C NMR (101 MHz, methanol-*d*_4_): δ 169.3, 169.0, 161.3, 158.6, 149.5, 141.1, 136.6, 131.0, 130.8, 130.5, 130.4, 129.8, 127.2, 121.3, 119.3, 116.9, 114.4, 113.4, 112.6, 62.6, 58.2, 56.8, 55.6, 45.8, 40.9, 38.7, 31.0, 29.9, 29.1, 27.0, 26.4, 18.9, −4.5, −4.7. HRMS-ESI (*m*/*z*) calcd for C_39_H_58_N_3_O_6_Si [M + H]^+^ 692.4095. Found 692.4100. [α]_D_^20^ = −52.8 (*c* 1.0, CHCl_3_).

#### *N*^1^-((2*S*,3*R*)-3-((*tert*-Butyldimethylsilyl)oxy)-1-(3-hydroxyphenyl)-4-((2-(3-methoxyphenyl)propan-2-yl)amino)butan-2-yl)-*N*^3^-(4-hydroxybutyl)-*N*^3^-methylisophthalamide (**29b**)

The title compound was obtained as a colorless oil (75 mg, 64% yield) from compound **28b** (147 mg, 0.169 mmol, 1 equiv) and 10% palladium on carbon (36 mg, 0.034 mmol, 0.2 equiv), following general procedure H. ^1^H NMR (400 MHz, methanol-*d*_4_): δ 7.91–7.83 (m, 1H), 7.79 (br s, 1H), 7.64–7.55 (m, 2H), 7.26 (t, *J* = 8.0 Hz, 1H), 7.09 (t, *J* = 8.1 Hz, 1H), 7.06–7.00 (m, 2H), 6.83 (dd, *J* = 8.1, 2.4 Hz, 1H), 6.71–6.66 (m, 2H), 6.64 (ddd, *J* = 8.1, 2.3, 1.2 Hz, 1H), 4.45 (td, *J* = 7.6, 4.6 Hz, 1H), 3.82 (td, *J* = 4.4, 2.9 Hz, 1H), 3.76 (s, 3H), 3.66 (t, *J* = 6.4 Hz, 1H), 3.61 (t, *J* = 7.3 Hz, 1H), 3.46 (t, *J* = 6.3 Hz, 1H), 3.33–3.30 (m, 1H), 3.11 (s, 1.5H), 2.99 (s, 1.5H), 2.87 (dd, *J* = 13.7, 7.7 Hz, 1H), 2.75–2.66 (m, 2H), 2.62 (dd, *J* = 12.0, 4.4 Hz, 1H), 1.84–1.73 (m, 1H), 1.73–1.59 (m, 2H), 1.54 (s, 3H), 1.53 (s, 3H), 1.37 (dt, *J* = 9.0, 6.5 Hz, 1H), 0.90 (s, 9H), 0.03 (s, 3H), 0.00 (s, 3H). ^13^C NMR (101 MHz, methanol-*d*_4_): δ 173.1, 172.58, 168.8, 161.3, 158.6, 149.3, 141.1, 138.2, 138.1, 136.4, 130.8, 130.5, 130.4, 130.2, 130.1, 129.4, 129.3, 126.32, 126.28, 121.3, 119.3, 116.9, 114.4, 113.5, 112.6, 72.1, 62.5, 62.1, 58.1, 56.9, 55.6, 52.4, 45.8, 38.6, 38.0, 33.2, 30.8, 30.3, 30.0, 29.0, 26.5, 26.4, 25.7, 24.4, 18.9, −4.5, −4.7. HRMS-ESI (*m*/*z*) calcd for C_38_H_56_N_3_O_6_Si [M + H]^+^ 678.3938. Found 678.3958. [α]_D_^20^ = −37.0 (*c* 2.0, CHCl_3_).

#### *N*^1^-((2*S*,3*R*)-3-((*tert*-Butyldimethylsilyl)oxy)-1-(3-hydroxyphenyl)-4-((2-(3-methoxyphenyl)propan-2-yl)amino)butan-2-yl)-*N*^3^-(4-hydroxybutyl)-*N*^3^-(3,3,3-trifluoropropyl)isophthalamide (**29c**)

The title compound was obtained as a colorless oil (123 mg, 69% yield) from compound **28c** (220 mg, 0.231 mmol, 1 equiv) and 10% palladium on carbon (49 mg, 0.046 mmol, 0.2 equiv), following general procedure H. ^1^H NMR (400 MHz, methanol-*d*_4_): δ 7.90–7.83 (m, 1H), 7.82–7.78 (m, 1H), 7.63–7.50 (m, 2H), 7.23 (t, *J* = 7.9 Hz, 1H), 7.01–6.94 (m, 2H), 6.89 (t, *J* = 7.6 Hz, 1H), 6.79 (ddd, *J* = 8.2, 2.5, 0.9 Hz, 1H), 6.48–6.42 (m, 2H), 6.31 (dt, *J* = 7.4, 1.4 Hz, 1H), 4.42 (ddd, *J* = 8.9, 6.7, 3.9 Hz, 1H), 3.82 (td, *J* = 4.4, 2.6 Hz, 1H), 3.79–3.74 (m, 1H), 3.72 (s, 3H), 3.67–3.36 (m, 3H), 3.31–3.26 (m, 1H), 2.88 (dd, *J* = 13.6, 6.8 Hz, 1H), 2.72 (dd, *J* = 12.0, 2.6 Hz, 1H), 2.68–2.40 (m, 4H), 1.83–1.55 (m, 3H), 1.50 (s, 3H), 1.49 (s, 3H), 1.36–1.30 (m, 1H), 0.84 (s, 9H), −0.04 (s, 3H), −0.08 (s, 3H). ^13^C NMR (101 MHz, methanol-*d*_4_): δ 173.3, 168.44, 168.40, 161.3, 149.5, 139.7, 137.9, 136.8, 130.6, 130.4, 130.2, 130.1, 129.3, 127.9 (q, *J* = 272.5 Hz), 126.2, 120.9, 119.3, 118.2, 115.5, 113.3, 112.7, 71.4, 62.0, 58.6, 56.7, 55.7, 51.1, 45.6, 40.3, 39.1, 32.3 (q, *J* = 28.0 Hz), 30.9, 30.4, 30.22, 29.3, 26.6, 26.4, 26.2, 24.9, 18.9, −4.6. HRMS-ESI (*m*/*z*) calcd for C_41_H_59_N_3_O_6_Si [M + H]^+^ 774.4125. Found 774.4122. [α]_D_^20^ = −24.4 (*c* 1.0, CHCl_3_).

#### *N*^1^-((2*S*,3*R*)-3-((*tert*-butyldimethylsilyl)oxy)-1-(3-hydroxyphenyl)-4-((2-(3-methoxyphenyl)propan-2-yl)amino)butan-2-yl)-*N*^3^-(2-(dimethylamino)ethyl)-*N*^3^-(4-hydroxybutyl)isophthalamide (**29d**)

The title compound was obtained as a colorless oil (292 mg, 68% yield) from compound **28d** (534 mg, 0.575 mmol, 1 equiv) and 10% palladium on carbon (245 mg, 0.115 mmol, 0.2 equiv), following general procedure H. ^1^H NMR (400 MHz, methanol-*d*_4_): δ 7.89–7.85 (m, 1H), 7.84–7.77 (m, 1H), 7.62–7.53 (m, 2H), 7.23 (t, *J* = 8.0 Hz, 1H), 7.02–6.94 (m, 2H), 6.89 (t, *J* = 7.7 Hz, 1H), 6.79 (ddd, *J* = 8.2, 2.6, 0.8 Hz, 1H), 6.48–6.41 (m, 2H), 6.31 (dt, *J* = 7.5, 1.3 Hz, 1H), 4.42 (ddd, *J* = 10.2, 6.6, 3.8 Hz, 1H), 3.84–3.80 (m, 1H), 3.73 (s, 3H), 3.70–3.59 (m, 2H), 3.59–3.49 (m, 1H), 3.45–3.39 (m, 1H), 3.39–3.34 (m, 1H), 3.29–3.26 (m, 1H), 2.94–2.84 (m, 1H), 2.77–2.68 (m, 1H), 2.66–2.59 (m, 1H), 2.59–2.52 (m, 1H), 2.51–2.39 (m, 2H), 2.39–1.99 (m, 6H), 1.82–1.69 (m, 1H), 1.69–1.56 (m, 2H), 1.50 (s, 6H), 1.36–1.31 (m, 1H), 0.83 (s, 9H), −0.05 (s, 3H), −0.08 (s, 3H). ^13^C NMR (101 MHz, methanol-*d*_4_): δ 173.2, 168.4, 168.30, 161.3, 149.5, 139.7, 138.3, 136.6, 130.5, 130.4, 130.2, 129.2, 129.1, 126.22, 126.17, 120.9, 119.3, 118.2, 115.4, 113.3, 112.6, 71.3, 62.5, 62.1, 58.6, 58.3, 57.3, 56.7, 55.7, 50.9, 45.8, 45.6, 44.1, 39.2, 31.0, 30.8, 30.5, 30.3, 30.2, 29.4, 26.4, 26.3, 25.2, 18.9, −4.58, −4.60. HRMS-ESI (*m*/*z*) calcd for C_42_H_65_N_4_O_6_Si [M + H]^+^ 794.4673. Found 794.4667. [α]_D_^20^ = −44.7 (*c* 0.9, CHCl_3_).

#### (*S*)-4-((*R*)-1-Hydroxy-2-((2-(3-methoxyphenyl)propan-2-yl)amino)ethyl)-7-oxa-3,12-diaza-1,6(1,3)-dibenzenacyclotridecaphane-2,13-dione (**7b**)

The intramolecular Mitsunobu reaction (cyclization) was performed using compound **29a** (61 mg, 0.090 mmol, 1 equiv), DTBAD (41 mg, 0.18 mmol, 2 equiv), and PPh_3_ (47 mg, 0.18 mmol, 2 equiv), following general procedure I. The desilylation was performed using NH_4_F (73 mg, 2.7 mmol, 30 equiv), following procedure F. Full conversion was achieved after stirring at 40 °C overnight. The crude product was purified by column chromatography on silica gel using gradient elution from DCM to 10% MeOH in DCM. Additional purification by preparative reverse phase HPLC using gradient elution from 10 to 95% MeCN in 0.1% aqueous HCOOH solution afforded product **7b** as an amorphous solid (13 mg, 26% yield). ^1^H NMR (400 MHz, methanol-*d*_4_): δ 7.84 (dt, *J* = 7.7, 1.5 Hz, 1H), 7.64 (dt, *J* = 7.7, 1.4 Hz, 1H), 7.49 (t, *J* = 7.7 Hz, 1H), 7.36–7.34 (m, 1H), 7.21 (t, *J* = 7.9 Hz, 1H), 7.15 (t, *J* = 7.9 Hz, 1H), 7.05–6.99 (m, 3H), 6.86 (dt, *J* = 7.9, 1.2 Hz, 1H), 6.80–6.71 (m, 2H), 4.22 (dddd, *J* = 18.9, 11.6, 6.7, 4.3 Hz, 2H), 4.15–4.06 (m, 1H), 3.76 (s, 3H), 3.64–3.56 (m, 2H), 3.46 (ddd, *J* = 13.7, 6.9, 3.8 Hz, 1H), 3.24 (dd, *J* = 13.7, 4.1 Hz, 1H), 2.72–2.59 (m, 1H), 2.54–2.41 (m, 2H), 2.03–1.92 (m, 2H), 1.92–1.84 (m, 2H), 1.49 (s, 3H), 1.48 (s, 3H). ^13^C NMR (101 MHz, methanol-*d*_4_): δ 170.6, 169.8, 161.2, 159.6, 149.8, 141.8, 136.9, 136.2, 131.7, 131.6, 131.0, 130.3, 130.2, 125.5, 122.2, 119.3, 118.0, 113.7, 113.3, 112.4, 74.6, 68.3, 56.9, 55.7, 55.6, 47.3, 41.3, 37.7, 30.1, 29.1, 28.8, 26.5. HRMS-ESI (*m*/*z*) calcd for C_32_H_40_N_3_O_5_ [M + H]^+^ 546.2968. Found 546.2956. [α]_D_^20^ = −30.3 (*c* 1.0, CHCl_3_).

#### (*S*)-4-((*R*)-1-Hydroxy-2-((2-(3-methoxyphenyl)propan-2-yl)amino)ethyl)-12-methyl-7-oxa-3,12-diaza-1,6(1,3)-dibenzenacyclotridecaphane-2,13-dione (**7c**)

The intramolecular Mitsunobu reaction (cyclization) was performed using compound **29b** (33 mg, 0.048 mmol, 1 equiv), DTBAD (22 mg, 0.10 mmol, 2 equiv), and PPh_3_ (25 mg, 0.10 mmol, 2 equiv), following general procedure I. The desilylation was performed using NH_4_F (53 mg, 1.43 mmol, 30 equiv), following procedure F. Full conversion was achieved after stirring at 40 °C overnight. Purification by column chromatography on silica gel using gradient elution from DCM to 10% MeOH in DCM afforded product **7c** as an amorphous solid (15 mg, 56% yield). ^1^H NMR (400 MHz, methanol-*d*_4_) for the mixture of rotamers: δ 7.73–7.24 (m, 4H), 7.24–6.98 (m, 4H), 6.97–6.64 (m, 4H), 4.68–4.03 (m, 2H), 3.94–3.59 (m, 5H), 3.31–2.86 (m, 5H), 2.72–2.19 (m, 4H), 2.03–1.83 (m, 1H), 1.82–1.71 (m, 1H), 1.70–1.39 (m, 8H). ^13^C NMR (101 MHz, methanol-*d*_4_) for the mixture of rotamers: δ 173.12, 173.05, 171.2, 169.0, 161.41, 161.35, 159.6, 159.1, 147.7, 141.8, 141.6, 138.1, 138.0, 136.6, 135.3, 131.0, 130.9, 130.69, 130.65, 130.57, 130.51, 130.3, 130.1, 129.8, 125.5, 124.9, 122.6, 121.6, 119.29, 119.24, 118.1, 116.0, 115.6, 113.9, 113.6, 113.4, 113.34, 113.25, 113.16, 73.8, 73.4, 67.8, 67.1, 58.9, 58.4, 56.2, 56.1, 55.72, 55.67, 51.9, 47.9, 47.4, 38.2, 38.1, 37.3, 37.2, 33.0, 28.9, 28.4, 28.31, 28.25, 26.4, 26.0, 22.4. HRMS-ESI (*m*/*z*) calcd for C_33_H_42_N_3_O_5_ [M + H]^+^ 560.3124. Found 560.3129. [α]_D_^20^ = 24.5 (*c* 0.7, CHCl_3_).

#### (*S*)-4-((*R*)-1-Hydroxy-2-((2-(3-methoxyphenyl)propan-2-yl)amino)ethyl)-12-(3,3,3-trifluoropropyl)-7-oxa-3,12-diaza-1,6(1,3)-dibenzenacyclotridecaphane-2,13-dione (**7d**)

The intramolecular Mitsunobu reaction (cyclization) was performed using compound **29c** (100 mg, 0.129 mmol, 1 equiv), DTBAD (89 mg, 0.39 mmol, 3 equiv), and PPh_3_ (102 mg, 0.388 mmol, 3 equiv), following general procedure I. The desilylation was performed using NH_4_F (104 mg, 3.87 mmol, 30 equiv), following procedure F. Full conversion was achieved after stirring at 40 °C overnight. Purification by column chromatography on silica gel using gradient elution from DCM to 10% MeOH in DCM afforded product **7d** as an amorphous solid (31 mg, 47% yield). ^1^H NMR (400 MHz, methanol-*d*_4_) for the mixture of rotamers: δ 7.67 (dt, *J* = 7.7, 1.6 Hz, 1H), 7.61–7.55 (m, 1H), 7.51 (t, *J* = 7.6 Hz, 1H), 7.48–7.42 (m, 1H), 7.16 (t, *J* = 8.0 Hz, 1H), 7.14–7.08 (m, 1H), 7.06–6.94 (m, 2H), 6.85–6.79 (m, 1H), 6.74–6.69 (m, 1H), 6.68–6.61 (m, 2H), 4.16 (ddd, *J* = 11.3, 7.8, 3.4 Hz, 1H), 3.84–3.74 (m, 4H), 3.72 (s, 3H), 3.71–3.66 (m, 1H), 3.29–3.10 (m, 3H), 2.72–2.57 (m, 3H), 2.54 (dd, *J* = 11.9, 3.3 Hz, 1H), 2.42 (dd, *J* = 11.9, 7.5 Hz, 1H), 1.84–1.68 (m, 1H), 1.68–1.53 (m, 2H), 1.53–1.39 (m, 7H). ^13^C NMR (101 MHz, methanol-*d*_4_) for a major rotamer: δ 173.3, 168.9, 161.2, 159.1, 149.9, 141.9, 137.8, 135.9, 130.7, 130.43, 130.37, 130.29, 129.7, 127.9 (q, *J* = 276.1 Hz), 124.9, 122.8, 119.4, 117.7, 113.7, 113.2, 112.5, 74.2, 67.6, 56.9, 56.4, 55.7, 50.4, 47.4, 40.1 (q, *J* = 4.4 Hz), 37.5, 32.3 (q, *J* = 28.0 Hz), 30.1, 28.9, 26.5, 26.4. HRMS-ESI (*m*/*z*) calcd for C_35_H_43_N_3_O_5_F_3_ [M + H]^+^ 642.3155. Found 642.3151. [α]_D_^20^ = −19.7 (*c* 1.0, CHCl_3_).

#### (*S*)-12-(2-(Dimethylamino)ethyl)-4-((*R*)-1-hydroxy-2-((2-(3-methoxyphenyl)propan-2-yl)amino)ethyl)-7-oxa-3,12-diaza-1,6(1,3)-dibenzenacyclotridecaphane-2,13-dione (**7f**)

The intramolecular Mitsunobu reaction (cyclization) was performed using compound **29d** (114 mg, 0.152 mmol, 1 equiv), DTBAD (88 mg, 0.38 mmol, 2.5 equiv), and PPh_3_ (100 mg, 0.381 mmol, 2 equiv), following general procedure I. The desilylation was performed using NH_4_F (149 mg, 4.56 mmol, 30 equiv), following procedure F. Full conversion was achieved after stirring at 40 °C overnight. The crude product was purified by column chromatography on silica gel using gradient elution from DCM to 50% MeOH in DCM. Additional purification by column chromatography on amino-functionalized silica gel using DCM afforded product **7f** as an amorphous solid (33 mg, 40% yield). ^1^H NMR (400 MHz, methanol-*d*_4_) for the mixture of rotamers: δ 7.75–7.64 (m, 1H), 7.64–7.54 (m, 1H), 7.54–7.46 (m, 2H), 7.27–7.08 (m, 2H), 7.08–6.94 (m, 2H), 6.91–6.80 (m, 1H), 6.80–6.72 (m, 1H), 6.72–6.60 (m, 2H), 4.54–4.07 (m, 2H), 3.94–3.50 (m, 7H), 3.29–2.93 (m, 3H), 2.72–2.19 (m, 10H), 2.08–1.85 (m, 2H), 1.81–1.70 (m, 1H), 1.69–1.38 (m, 8H). ^13^C NMR (101 MHz, methanol-*d*_4_) for a major rotamer: δ 171.8, 167.4, 159.8, 157.7, 148.0, 140.4, 136.7, 134.1, 129.6, 129.0, 128.9, 128.3, 123.4, 121.3, 117.9, 116.7, 114.4, 112.2, 111.8, 111.2, 72.6, 66.4, 55.8, 55.7, 54.9, 54.2, 49.0, 45.9, 44.3, 44.0, 42.4, 36.0, 28.5, 27.3, 25.3, 25.1. HRMS-ESI (*m*/*z*) calcd for C_36_H_49_N_4_O_5_ [M + H]^+^ 617.3703. Found 617.3723. [α]_D_^20^ = 18.7 (*c* 1.0, CHCl_3_).

#### *tert*-Butyl ((2*S*,3*R*)-3-((*tert*-Butyldimethylsilyl)oxy)-1-(3-hydroxyphenyl)-4-((2-(3-methoxyphenyl)propan-2-yl)amino)butan-2-yl)carbamate (**30**)

To a solution of compound **16** (440 mg, 0.678 mmol, 1 equiv) in MeOH (12 mL), 10% palladium on carbon (72 mg, 0.068 mmol, 0.1 equiv) was added. The reaction mixture was stirred under a H_2_ atmosphere (1 atm) for 3 h. Carbon was filtered using a syringe filter (Teflon; 0.45 μm, washed with MeOH). The filtrate was evaporated under reduced pressure. Purification by column chromatography on silica gel using gradient elution from 10 to 50% EtOAc in hexanes afforded product **30** as a colorless oil (304 mg, 80% yield). ^1^H NMR (400 MHz, chloroform-*d*): δ 7.26 (t, *J* = 8.0 Hz, 1H), 7.09 (t, *J* = 7.7 Hz, 1H), 7.06–6.96 (m, 2H), 6.78 (ddd, *J* = 8.2, 2.5, 0.9 Hz, 1H), 6.75–6.65 (m, 2H), 6.62 (t, *J* = 1.9 Hz, 1H), 6.44–6.02 (m, 1H), 4.02–3.90 (m, 1H), 3.82 (s, 3H), 3.73–3.57 (m, 1H), 2.83–2.65 (m, 1H), 2.56–2.39 (m, 3H), 1.46 (s, 3H), 1.43 (s, 3H), 1.42–1.22 (m, 9H), 0.87 (s, 9H), −0.03 (s, 3H), −0.07 (s, 3H). ^13^C NMR (101 MHz, chloroform-*d*): δ 159.6, 156.3, 156.2, 149.3, 140.4, 129.6, 129.3, 121.2, 118.6, 116.1, 113.4, 112.7, 111.4, 78.9, 71.4, 56.6, 55.6, 55.4, 44.7, 37.4, 29.7, 29.4, 28.6, 26.0, 18.2, −4.7, −4.8. HRMS-ESI (*m*/*z*) calcd for C_31_H_51_N_2_O_5_Si [M + H]^+^ 559.3567. Found 559.3563. [α]_D_^20^ = −22.3 (*c* 1.3, CHCl_3_).

#### 3-((2*S*,3*R*)-2-((*tert*-Butoxycarbonyl)amino)-3-((*tert*-Butyldimethylsilyl)oxy)-4-((2-(3-methoxyphenyl)propan-2-yl)amino)butyl)phenyl trifluoromethanesulfonate (**31**)

To a solution of compound **30** (1.12 g, 2.00 mmol, 1 equiv) in DCM (25 mL), triethylamine (1.12 mL, 8.02 mmol, 4 equiv) was added, followed by *N*-phenyl-bis(trifluoromethanesulfonimide) (1.43 g, 4.01 mmol, 2 equiv). The reaction mixture was stirred at room temperature for 1 h. The reaction mixture was concentrated under reduced pressure. The residue was dissolved in EtOAc (40 mL), washed sequentially with 1 M aqueous HCl (20 mL), 1 M aqueous NaOH (20 mL), and brine (20 mL), dried over anhydrous Na_2_SO_4_, and evaporated under reduced pressure. Purification by column chromatography on silica gel using gradient elution from 1 to 5% MeOH in DCM afforded product **31** as a colorless oil (1.16 g, 84% yield). ^1^H NMR (400 MHz, chloroform-*d*): δ 7.33 (t, *J* = 8.0 Hz, 1H), 7.26 (d, *J* = 7.8 Hz, 1H), 7.22 (d, *J* = 8.0 Hz, 1H), 7.13–7.07 (m, 1H), 7.06–7.04 (m, 1H), 7.03–6.99 (m, 2H), 6.77 (ddd, *J* = 8.1, 2.5, 0.9 Hz, 1H), 4.03–3.89 (m, 1H), 3.80 (s, 3H), 3.72–3.58 (m, 1H), 2.77 (dd, *J* = 14.0, 8.5 Hz, 1H), 2.64 (dd, *J* = 14.1, 6.5 Hz, 1H), 2.59–2.44 (m, 2H), 1.45 (s, 3H), 1.44 (s, 3H), 1.41–1.25 (m, 9H), 0.87 (s, 9H), −0.02 (s, 3H), −0.05 (s, 3H). ^13^C NMR (101 MHz, chloroform-*d*): δ 159.8, 155.8, 149.6, 149.2, 142.3, 130.1, 129.4, 129.3, 122.2, 119.0, 118.5, 112.7, 111.3, 78.9, 71.8, 56.2, 55.6, 55.3, 45.0, 37.0, 29.8, 29.2, 28.5, 25.9, 18.2. HRMS-ESI (*m*/*z*) calcd for C_32_H_50_N_2_O_7_SiSF_3_ [M + H]^+^ 691.3060. Found 691.3070. [α]_D_^20^ = −28.8 (*c* 1.2, CHCl_3_).

#### *tert*-Butyl ((2*S*,3*R*)-3-((*tert*-Butyldimethylsilyl)oxy)-4-((2-(3-methoxyphenyl)propan-2-yl)amino)-1-(3-vinylphenyl)butan-2-yl)carbamate (**32**)

To a solution of compound **31** (600 mg, 0.869 mmol, 1 equiv) in DMF (20 mL, sparged with argon), Pd(PPh_3_)_4_ (251 mg, 0.217 mmol, 0.25 equiv) was added, followed by 0.5 M LiCl solution in THF (5.21 mL, 2.61 mmol, 3 equiv). Tri-*n*-butyl(vinyl)tin (508 μL, 1.74 mmol, 2 equiv) was added, and the reaction mixture was stirred at 60 °C for 20 h. The solvent was evaporated under reduced pressure, and the residue was dissolved in diethyl ether (30 mL) and water (30 mL). The aqueous phase was separated and washed with diethyl ether (30 mL). The combined organic phase was washed with brine (20 mL), dried over anhydrous Na_2_SO_4_, and evaporated under reduced pressure. Purification by column chromatography on silica gel/powdered K_2_CO_3_ mixture (9:1 w/w) using 10% EtOAc in hexanes afforded product **32** as a colorless oil (390 mg, 79% yield). ^1^H NMR (400 MHz, chloroform-*d*): δ 7.25–7.12 (m, 4H), 7.07–6.96 (m, 3H), 6.77 (ddd, *J* = 8.2, 2.4, 1.0 Hz, 1H), 6.67 (dd, *J* = 17.6, 10.9 Hz, 1H), 6.48–6.20 (m, 1H), 5.72 (dd, *J* = 17.6, 1.0 Hz, 1H), 5.21 (dd, *J* = 10.9, 1.0 Hz, 1H), 4.05–3.93 (m, 1H), 3.80 (s, 3H), 3.72–3.59 (m, 1H), 2.83 (dd, *J* = 14.0, 7.7 Hz, 1H), 2.57 (dd, *J* = 13.7, 7.7 Hz, 1H), 2.50 (d, *J* = 4.2 Hz, 2H), 1.45 (s, 3H), 1.43 (s, 3H), 1.41–1.26 (m, 9H), 0.87 (s, 9H), −0.03 (s, 3H), −0.08 (s, 3H). ^13^C NMR (101 MHz, chloroform-*d*): δ 159.8, 155.9, 149.4, 139.1, 137.7, 137.0, 129.3, 128.7, 128.6, 127.2, 124.1, 118.5, 113.8, 112.5, 111.4, 78.6, 71.4, 56.5, 55.6, 55.3, 44.8, 37.4, 29.8, 29.3, 28.6, 26.0, 18.2, −4.7, −4.8. HRMS-ESI (*m*/*z*) calcd for C_33_H_53_N_2_O_4_Si [M + H]^+^ 569.3775. Found 569.3773. [α]_D_^20^ = −16.9 (*c* 1.2, CHCl_3_).

#### *tert*-Butyl ((2*S*,3*R*)-3-((*tert*-Butyldimethylsilyl)oxy)-1-(3-(2-hydroxyethyl)phenyl)-4-((2-(3-methoxyphenyl)propan-2-yl)amino)butan-2-yl)carbamate (**33**)

A solution of compound **32** (160 mg, 0.281 mmol, 1 equiv) in THF (10 mL) was cooled to 0 °C. Borane-dimethyl sulfide complex (160 μL, 1.69 mmol, 6 equiv) was added dropwise. The ice bath was removed, and the reaction mixture was left stirring at room temperature for 2 h. Then, 1 M aqueous NaOH solution (2.8 mL, 2.8 mmol, 10 equiv) and water (2.5 mL) were added, followed by 35% aqueous hydrogen peroxide solution (745 μL, 8.44 mmol, 30 equiv). The reaction mixture was stirred at room temperature for 10 min and then at 50 °C for 1 h. The reaction mixture was extracted with DCM (3 × 20 mL). The combined organic phase was washed with brine (30 mL), dried over anhydrous Na_2_SO_4_, and evaporated under reduced pressure. Purification by column chromatography on silica gel using gradient elution from DCM to 5% MeOH in DCM afforded product **33** as a colorless oil (130 mg, 79% yield). ^1^H NMR (400 MHz, chloroform-*d*): δ 7.26 (t, *J* = 7.9 Hz, 1H), 7.22–7.13 (m, 1H), 7.13–7.05 (m, 1H), 7.05–6.95 (m, 4H), 6.77 (ddd, *J* = 8.2, 2.5, 0.9 Hz, 1H), 6.12 (d, *J* = 8.5 Hz, 1H), 4.07–3.93 (m, 1H), 3.92–3.73 (m, 5H), 3.72–3.57 (m, 1H), 2.89–2.73 (m, 2H), 2.73–2.56 (m, 2H), 2.51 (d, *J* = 4.3 Hz, 2H), 1.46 (s, 3H), 1.44 (s, 3H), 1.33 (s, 9H), 0.87 (s, 9H), −0.01 (s, 3H), −0.05 (s, 3H). ^13^C NMR (101 MHz, chloroform-*d*): δ 159.7, 155.9, 149.4, 139.0, 138.8, 130.1, 129.3, 128.6, 127.7, 127.0, 118.5, 112.5, 111.3, 71.9, 63.8, 56.5, 55.6, 55.3, 45.0, 39.4, 37.5, 29.7, 29.3, 28.5, 26.0, 18.2, −4.6, −4.7. HRMS-ESI (*m*/*z*) calcd for C_33_H_55_N_2_O_5_Si [M + H]^+^ 587.3880. Found 587.3882. [α]_D_^20^ = −34.7 (*c* 1.0, CHCl_3_).

#### *tert*-Butyl ((2*S*,3*R*)-3-((*tert*-Butyldimethylsilyl)oxy)-4-((2-(3-methoxyphenyl)propan-2-yl)amino)-1-(3-(2-(2-oxo-2-(propylamino)ethoxy)ethyl)phenyl)butan-2-yl)carbamate (**35**)

A solution of compound **33** (1.4 g, 2.4 mmol, 1 equiv) in THF (50 mL) was cooled to −78 °C. Then, 1 M KHMDS solution in THF (3.58 mL, 3.58 mmol, 1.5 equiv) was added dropwise. The reaction mixture was stirred for 5 min, and then a solution of bromide **34** (644 mg, 3.58 mmol, 1.5 equiv) in THF (10 mL) was added. The reaction mixture was stirred for 1 h at −78 °C. The reaction mixture was quenched with water (10 mL) and extracted with EtOAc (2 × 30 mL). The combined organic phase was washed with brine (30 mL), dried over anhydrous Na_2_SO_4_, and evaporated under reduced pressure. Purification by column chromatography on silica gel using gradient elution from 10% EtOAc to 50% EtOAc in hexanes afforded product **35** as a colorless oil (1.20 g, 73% yield). ^1^H NMR (400 MHz, chloroform-*d*): δ 7.24 (t, *J* = 7.9 Hz, 1H), 7.18 (t, *J* = 7.5 Hz, 1H), 7.07–6.94 (m, 5H), 6.76 (ddd, *J* = 8.2, 2.5, 1.0 Hz, 1H), 6.45–6.24 (m, 2H), 4.00–3.93 (m, 1H), 3.98 (s, 1H), 3.91 (s, 2H), 3.80 (s, 3H), 3.72–3.59 (m, 3H), 3.19–3.07 (m, 2H), 2.85 (t, *J* = 6.7 Hz, 2H), 2.82–2.75 (m, 1H), 2.59–2.52 (m, 1H), 2.52–2.45 (m, 2H), 1.51–1.23 (m, 17H), 0.86 (t, *J* = 7.3 Hz, 3H), 0.86 (s, 9H), −0.05 (s, 3H), −0.10 (s, 3H). ^13^C NMR (101 MHz, chloroform-*d*): δ 169.7, 159.8, 156.0, 149.3, 139.3, 138.6, 129.7, 129.3, 128.6, 127.5, 126.8, 118.5, 112.5, 111.3, 78.6, 72.7, 71.3, 70.3, 56.7, 55.5, 55.3, 44.7, 40.6, 37.5, 36.3, 29.7, 29.4, 28.6, 26.0, 22.9, 18.2, 11.5, −4.7, −4.8. HRMS-ESI (*m*/*z*) calcd for C_38_H_64_N_3_O_6_Si [M + H]^+^ 686.4564. Found 686.4579. [α]_D_^20^ = −20.6 (*c* 1.0, CHCl_3_).

#### Methyl 3-((2-(3-((2*S*,3*R*)-2-((*tert*-Butoxycarbonyl)amino)-3-((*tert*-butyldimethylsilyl)oxy)-4-((2-(3-methoxyphenyl)propan-2-yl)amino)butyl)phenethoxy)ethyl)(propyl)carbamoyl)benzoate (**36**)

To a solution of compound **35** (1.20 g, 1.75 mmol, 1 equiv) in THF (100 mL), borane-dimethyl sulfide complex was added. The reaction mixture was stirred at 75 °C for 4 h. The reaction mixture was cooled to 0 °C and was quenched by dropwise addition of MeOH (50 mL). Volatiles were evaporated under reduced pressure. The residue was dissolved in MeOH (30 mL) and evaporated again. The residue was dissolved in MeOH (50 mL), and 10% aqueous Rochelle′s salt solution (80 mL) was added. The mixture was stirred at 70 °C for 120 h. The reaction mixture was concentrated under reduced pressure, and the aqueous residue was extracted with EtOAc (3 × 40 mL). The combined organic phase was washed with brine (30 mL), dried over anhydrous Na_2_SO_4_, and evaporated under reduced pressure. Purification by column chromatography on silica gel using gradient elution from DCM to 10% MeOH in DCM afforded crude amine (620 mg), which was used in the next step without further purification. Amide coupling was performed using crude amine from the previous step (620 mg, 0.92 mmol, 1 equiv), acid **19** (199 mg, 1.11 mmol, 1.2 equiv), DIPEA (319 μL, 1.84 mmol, 2 equiv), and HBTU (420 mg, 1.11 mmol, 1.2 equiv), following procedure G. Purification by column chromatography on silica gel using gradient elution from 10% EtOAc to 50% EtOAc in hexanes afforded product **36** as a colorless oil (480 mg, 33% yield over 2 steps). ^1^H NMR (400 MHz, methanol-*d*_4_) for 2 rotamers: δ 8.13–7.93 (m, 2H), 7.62–7.42 (m, 2H), 7.23 (t, *J* = 8.2 Hz, 1H), 7.15 (t, *J* = 7.5 Hz, 1H), 7.11–6.93 (m, 5H), 6.83–6.71 (m, 1H), 3.94–3.83 (m, 4H), 3.77 (s, 3H), 3.75–3.63 (m, 4H), 3.57 (t, *J* = 7.0 Hz, 1H), 3.52–3.44 (m, 2H), 3.44–3.37 (m, 1H), 3.18–3.11 (m, 1H), 2.91–2.78 (m, 2H), 2.75–2.64 (m, 1H), 2.60–2.33 (m, 3H), 1.73–1.59 (m, 1H), 1.54–1.40 (m, 7H), 1.39–1.20 (m, 9H), 0.96 (t, *J* = 7.4 Hz, 1.6H), 0.90 (s, 9H), 0.68 (t, *J* = 7.4 Hz, 1.4H), 0.03 (s, 3H), −0.02 (s, 3H). ^13^C NMR (101 MHz, methanol-*d*_4_) for 2 rotamers: δ 173.0, 167.6, 161.3, 157.8, 150.0, 140.5, 140.3, 140.2, 138.5, 138.4, 134.8, 132.7, 132.1, 131.6, 131.4, 131.3, 130.9, 130.8, 130.3, 130.1, 129.9, 129.31, 129.25, 128.5, 128.1, 127.8, 119.4, 113.4, 112.5, 79.8, 74.1, 73.2, 69.5, 68.9, 66.4, 59.6, 57.3, 56.8, 56.6, 55.7, 53.2, 52.9, 49.5, 46.3, 46.2, 38.0, 37.3, 37.2, 29.9, 29.1, 28.9, 28.5, 26.5, 22.7, 21.6, 19.0, 11.7, 11.3, −4.2, −4.5. HRMS-ESI (*m*/*z*) calcd for C_47_H_72_N_3_O_8_Si [M + H]^+^ 834.5089. Found 834.5102. [α]_D_^20^ = −11.6 (*c* 1.1, CHCl_3_).

#### (*S*)-4-((*R*)-1-Hydroxy-2-((2-(3-methoxyphenyl)propan-2-yl)amino)ethyl)-12-propyl-9-oxa-3,12-diaza-1,6(1,3)-dibenzenacyclotridecaphane-2,13-dione (**7i**)

To a solution of compound **36** (440 mg, 0.528 mmol, 1 equiv) in methanol (15 mL), 1 M aqueous NaOH solution (1.05 mL, 1.05 mmol, 2 equiv) was added, and the reaction mixture was stirred at 55 °C overnight. The solvent was evaporated under reduced pressure, and the residue was dissolved in 4 M HCl solution in dioxane (2.64 mL, 10.5 mmol, 20 mmol) and 6 M aqueous HCl solution (8.80 mL, 52.7 mmol, 100 equiv). The heterogeneous reaction mixture was stirred at 60 °C overnight. The resulting homogenous mixture was evaporated under reduced pressure. The residue was dissolved in DCM (40 mL). DIPEA (365 μL, 2.11 mmol, 4 equiv) was added, followed by HBTU (300 mg, 0.80 mmol, 1.5 equiv). The reaction mixture was washed with water (30 mL) and brine (30 mL), dried over anhydrous Na_2_SO_4_, and evaporated under reduced pressure. Purification by column chromatography on silica gel using gradient elution from DCM to 10% MeOH in DCM afforded product **7i** as a colorless oil (156 mg, 49% yield). ^1^H NMR (400 MHz, methanol-*d*_4_) for a mixture of rotamers (0.55:0.35:0.1): δ 7.67–7.35 (m, 3.6H), 7.28–6.99 (m, 7H), 6.82–6.71 (m, 1H), 6.61–6.43 (m, 0.4H)4.55–3.84 (m, 2H), 3.83–3.56 (m, 8H), 3.44–3.35 (m, 2H), 3.29–2.99 (m, 2H), 2.96–2.64 (m, 3H), 2.58–2.41 (m, 2H), 1.73 (dddt, *J* = 27.1, 13.3, 8.5, 6.9 Hz, 1H), 1.50 (dd, *J* = 8.0, 3.9 Hz, 6H), 1.43–1.20 (m, 1H), 1.03 (t, *J* = 7.4 Hz, 1.65H), 0.74 (t, *J* = 7.4 Hz, 0.25H), 0.62 (t, *J* = 7.4 Hz, 1.1H). ^13^C NMR (101 MHz, methanol-*d*_4_) for two major rotamers: δ 173.8, 173.0, 171.1, 170.3, 161.2, 150.0, 149.9, 141.3, 140.4, 140.2, 140.0, 138.3, 137.7, 136.83, 136.80, 131.6, 131.5, 130.4, 130.29, 130.25, 130.20, 130.0, 129.7, 129.53, 129.49, 129.0, 128.8, 127.8, 127.0, 125.88, 125.86, 125.79, 119.3, 113.3, 113.2, 112.5, 112.4, 75.0, 74.6, 73.3, 72.7, 69.8, 67.7, 56.86, 56.84, 56.1, 55.7, 54.1, 53.5, 47.3, 46.7, 46.2, 37.6, 37.5, 37.4, 37.1, 30.2, 30.1, 29.0, 28.8, 22.5, 21.5, 11.7, 11.1. HRMS-ESI (*m*/*z*) calcd for C_35_H_46_N_3_O_5_ [M + H]^+^ 588.3437. Found 588.3450. [α]_D_^20^ = 34.7 (*c* 0.9, CHCl_3_).

#### (2*R*,3*S*)-2-((*tert*-Butyldimethylsilyl)oxy)-*N*^1^-(2-(3-methoxyphenyl)propan-2-yl)-4-(3-vinylphenyl)butane-1,3-diamine (**37**)

To a solution of compound **32** (400 mg, 0.703 mmol, 1 equiv) in DCM (1 mL), trifluoroacetic acid (1.08 mL, 14.1 mmol, 20 equiv) was added. The reaction mixture was stirred for 1 h. Volatiles were evaporated under reduced pressure. Diethyl ether (25 mL) and 1 M aqueous NaOH (20 mL) were added. The aqueous phase was extracted with diethyl ether (25 mL). The combined organic phase was washed with brine (20 mL), dried over anhydrous Na_2_SO_4_, and evaporated under reduced pressure. Crude product **37** (314 mg, 95% yield) was used in the next step without further purification. ^1^H NMR (400 MHz, methanol-*d*_4_): δ 7.32–7.21 (m, 4H), 7.05 (dt, *J* = 7.0, 1.8 Hz, 1H), 7.03–6.97 (m, 2H), 6.78 (ddd, *J* = 8.2, 2.5, 1.0 Hz, 1H), 6.71 (dd, *J* = 17.6, 10.9 Hz, 1H), 5.76 (dd, *J* = 17.6, 1.1 Hz, 1H), 5.22 (dd, *J* = 10.9, 1.1 Hz, 1H), 3.77 (s, 3H), 3.65 (td, *J* = 5.7, 4.1 Hz, 1H), 3.17 (ddd, *J* = 8.3, 6.3, 4.2 Hz, 1H), 2.72 (dd, *J* = 13.5, 6.3 Hz, 1H), 2.53–2.45 (m, 3H), 1.45 (s, 3H), 1.42 (s, 3H), 0.92 (s, 9H), 0.05 (s, 3H), 0.03 (s, 3H). ^13^C NMR (101 MHz, methanol-*d*_4_): δ 161.3, 150.2, 140.6, 139.3, 138.1, 130.3, 130.0, 129.7, 128.0, 125.3, 119.3, 114.2, 113.4, 112.4, 75.9, 57.6, 56.8, 55.7, 45.0, 40.0, 30.5, 28.7, 26.5, 19.0, −4.1, −4.4. HRMS-ESI (*m*/*z*) calcd for C_28_H_45_N_2_O_2_Si [M + H]^+^ 469.3251. Found 469.3256. [α]_D_^20^ = 4.3 (*c* 1.0, CHCl_3_).

#### *N*^1^-(But-3-en-1-yl)-*N*^3^-((2*S*,3*R*)-3-((*tert*-butyldimethylsilyl)oxy)-4-((2-(3-methoxyphenyl)propan-2-yl)amino)-1-(3-vinylphenyl)butan-2-yl)-*N*^1^-propylisophthalamide (**39a**)

The title compound was obtained as a colorless oil (280 mg, 60% yield) from amine **37** (305 mg, 0.651 mmol, 1 equiv), HBTU (247 mg, 0.651, 1 equiv), DIPEA (225 μL, 1.30 mmol, 2 equiv), and acid **38a** (170 mg, 0.651 mmol, 1 equiv), following general procedure G. A pure material was obtained by column chromatography on silica gel using 2% MeOH in DCM. ^1^H NMR (400 MHz, chloroform-*d*) for 2 rotamers: δ 9.56 (d, *J* = 6.7 Hz, 1H), 7.92–7.85 (m, 2H), 7.51–7.42 (m, 2H), 7.29–7.24 (m, 1H), 7.23–7.18 (m, 3H), 7.07 (dt, *J* = 7.0, 1.8 Hz, 1H), 7.00–6.93 (m, 2H), 6.78 (ddd, *J* = 8.2, 2.5, 0.9 Hz, 1H), 6.66 (dd, *J* = 17.6, 10.9 Hz, 1H), 5.93–5.78 (m, 0.5H), 5.71 (dd, *J* = 17.7, 1.0 Hz, 1H), 5.61–5.46 (m, 0.5H), 5.21 (dd, *J* = 10.8, 0.9 Hz, 1H), 5.18–4.91 (m, 2H), 4.51–4.41 (m, 1H), 3.75 (s, 3H), 3.65 (ddd, *J* = 4.7, 3.1, 1.3 Hz, 1H), 3.61–3.50 (m, 1H), 3.50–3.41 (m, 1H), 3.33–3.10 (m, 3H), 2.70 (dd, *J* = 11.8, 1.5 Hz, 1H), 2.57 (ddd, *J* = 11.8, 4.8, 1.2 Hz, 1H), 2.48–2.36 (m, 2H), 2.29–2.17 (m, 1H), 1.75–1.61 (m, 1H), 1.53 (s, 3H), 1.51 (s, 3H), 1.02–0.92 (m, 1H), 0.82–0.68 (m, 11H), −0.14 (s, 3H), −0.18 (s, 3H). ^13^C NMR (101 MHz, chloroform-*d*) for 2 rotamers: δ 171.1, 166.4, 159.9, 148.2, 138.6, 137.9, 137.8, 135.6, 134.2, 129.5, 129.4, 128.8, 128.7, 128.6, 127.6, 127.1, 125.3, 124.4, 118.3, 117.7, 116.9, 114.1, 112.6, 111.4, 68.7, 57.9, 55.7, 55.3, 51.1, 48.7, 46.7, 44.3, 44.2, 38.3, 33.3, 32.1, 29.8, 29.5, 25.9, 22.1, 20.8, 18.1, 11.6, 11.2, −4.9, −5.1. HRMS-ESI (*m*/*z*) calcd for C_43_H_62_N_3_O_4_Si [M + H]^+^ 712.4510. Found 712.4526. [α]_D_^20^ = −46.8 (*c* 1.4, CHCl_3_).

#### *N*^1^-((2*S*,3*R*)-3-((*tert*-Butyldimethylsilyl)oxy)-4-((2-(3-methoxyphenyl)propan-2-yl)amino)-1-(3-vinylphenyl)butan-2-yl)-*N*^3^-(pent-4-en-1-yl)-*N*^3^-propylisophthalamide (**39b**)

The title compound was obtained as a colorless oil (550 mg, 83% yield) from amine **37** (426 mg, 0.908 mmol, 1 equiv), HBTU (344 mg, 0.908 mmol, 1 equiv), DIPEA (314 μL, 1.82 mmol, 2 equiv), and acid **38b** (250 mg, 0.908 mmol, 1 equiv), following general procedure G. A pure material was obtained by column chromatography on silica gel using gradient elution from 10 to 50% EtOAc in hexanes. ^1^H NMR (400 MHz, chloroform-*d*) for 2 rotamers: δ 9.57 (d, *J* = 6.9 Hz, 1H), 7.93–7.83 (m, 2H), 7.51–7.42 (m, 2H), 7.30–7.18 (m, 4H), 7.06 (dt, *J* = 6.8, 1.8 Hz, 1H), 7.00–6.92 (m, 2H), 6.81–6.75 (m, 1H), 6.66 (dd, *J* = 17.6, 10.9 Hz, 1H), 5.95–5.76 (m, 1H), 5.84 (d, *J* = 7.2 Hz, 0.5H), 5.71 (dd, *J* = 17.6, 1.0 Hz, 1H), 5.64–5.54 (m, 0.5H), 5.21 (dd, *J* = 10.9, 1.0 Hz, 1H), 5.03–4.95 (m, 1H), 4.95–4.78 (m, 1H), 4.46 (tt, *J* = 8.8, 5.4 Hz, 1H), 3.75 (s, 3H), 3.68–3.62 (m, 1H), 3.56–3.35 (m, 2H), 3.26–3.09 (m, 3H), 2.70 (d, *J* = 11.7 Hz, 1H), 2.62–2.54 (m, 1H), 2.43 (dd, *J* = 13.8, 10.2 Hz, 1H), 2.16–2.08 (m, 1H), 1.91–1.82 (m, 1H), 1.80–1.70 (m, 1H), 1.70–1.62 (m, 1H), 1.62–1.58 (m, 1H), 1.58–1.55 (m, 1H), 1.53 (s, 3H), 1.51 (s, 3H), 1.00–0.94 (m, 1H), 0.82–0.69 (m, 11H), −0.14 (s, 3H), −0.18 (s, 3H). ^13^C NMR (101 MHz, chloroform-*d*) for 2 rotamers: δ 171.0, 166.4, 159.9, 148.2, 138.6, 137.96, 137.92, 137.8, 137.1, 136.8, 135.5, 129.5, 129.3, 128.8, 128.7, 127.6, 127.1, 125.3, 124.4, 118.3, 115.6, 115.2, 114.0, 112.6, 111.4, 68.7, 57.9, 55.7, 55.2, 51.0, 48.7, 46.7, 44.6, 44.1, 38.3, 31.4, 30.7, 29.8, 29.4, 27.9, 26.8, 25.8, 22.1, 20.9, 18.1, 11.6, 11.2, −4.9, −5.1. HRMS-ESI (*m*/*z*) calcd for C_44_H_64_N_3_O_4_Si [M + H]^+^ 726.4666. Found 726.4677. [α]_D_^20^ = −35.3 (*c* 2.5, CHCl_3_).

#### (*S*)-4-((*R*)-1-Hydroxy-2-((2-(3-methoxyphenyl)propan-2-yl)amino)ethyl)-11-propyl-3,11-diaza-1,6(1,3)-dibenzenacyclododecaphane-2,12-dione (**7j**)

Ring-closing metathesis and hydrogenation of the double bond were performed using compound **39a** (250 mg, 0.351 mmol, 1 equiv), Zhan Catalyst-1B (26 mg, 0.035 mmol, 0.1 equiv), and 10% palladium on carbon (37 mg, 0.035 mmol, 0.1 equiv), following general procedure J. Crude macrocycle **40a** (210 mg, 87% yield) was used in the next step without purification.

The desilylation was performed using crude macrocycle **40a** from the previous step (210 mg, 0.306 mmol, 1 equiv), NH_4_F (340 mg, 9.18 mmol, 30 equiv), following general procedure F. Full conversion was achieved after stirring at 40 °C overnight. Purification by column chromatography on silica gel using gradient elution from DCM to 10% MeOH in DCM afforded product **7j** as an amorphous solid (114 mg, 65% yield). ^1^H NMR (400 MHz, methanol-*d*_4_) δ 7.70 (dt, *J* = 7.8, 1.5 Hz, 1H), 7.50 (t, *J* = 7.7 Hz, 1H), 7.46–7.39 (m, 2H), 7.23–7.09 (m, 3H), 7.04–6.97 (m, 2H), 6.96–6.89 (m, 2H), 6.75 (ddd, *J* = 8.2, 2.5, 0.9 Hz, 1H), 4.15 (ddd, *J* = 10.8, 8.5, 3.5 Hz, 1H), 3.78–3.71 (m, 4H), 3.58–3.45 (m, 1H), 3.42–3.32 (m, 1H), 3.24 (dd, *J* = 13.6, 3.5 Hz, 1H), 3.05–2.92 (m, 2H), 2.68 (dd, *J* = 13.6, 11.0 Hz, 1H), 2.60–2.42 (m, 4H), 1.71 (h, *J* = 6.8, 6.2 Hz, 2H), 1.67–1.57 (m, 1H), 1.51 (s, 6H), 1.48–1.38 (m, 1H), 1.38–1.19 (m, 2H), 0.99 (t, *J* = 7.4 Hz, 3H). ^13^C NMR (101 MHz, methanol-*d*_4_): δ 173.2, 168.9, 161.3, 148.3, 142.8, 139.9, 138.2, 135.3, 131.0, 130.5, 130.3, 130.2, 129.6, 129.5, 128.0, 127.7, 124.9, 119.2, 113.2, 112.9, 73.3, 57.9, 56.6, 55.6, 50.9, 48.6, 47.4, 37.5, 35.9, 29.7, 29.30, 29.0, 28.4, 21.8, 11.7. HRMS-ESI (*m*/*z*) calcd for C_36_H_48_N_3_O_4_ [M + H]^+^ 572.3488. Found 572.3496. [α]_D_^20^ = −17.2 (*c* 0.5, CHCl_3_).

#### (*S*)-4-((*R*)-1-Hydroxy-2-((2-(3-methoxyphenyl)propan-2-yl)amino)ethyl)-12-propyl-3,12-diaza-1,6(1,3)-dibenzenacyclotridecaphane-2,13-dione (**7k**)

Ring-closing metathesis and hydrogenation of the double bond were performed using compound **39b** (430 mg, 0.592 mmol, 1 equiv), Zhan Catalyst-1B (43 mg, 0.059 mmol, 0.1 equiv), and 10% palladium on carbon (63 mg, 0.059 mmol, 0.1 equiv), following general procedure J. Crude macrocycle **40b** (250 mg, 60% yield) was used in the next step without purification.

The desilylation was performed using crude macrocycle **40b** from the previous step (250 mg, 0.357 mmol, 1 equiv), NH_4_F (397 mg, 10.7 mmol, 30 equiv), following general procedure F. Full conversion was achieved after stirring at 50 °C for 16 h. Purification by column chromatography on silica gel using gradient elution from DCM to 10% MeOH in DCM afforded product **7k** as an amorphous solid (140 mg, 67% yield). ^1^H NMR (400 MHz, methanol-*d*_4_) for a mixture of rotamers (0.80:0.13:0.07): δ 7.71–7.51 (m, 2H), 7.48 (t, *J* = 7.7 Hz, 1H), 7.39 (dt, *J* = 7.6, 1.4 Hz, 1H), 7.25–7.09 (m, 2H), 7.09–6.96 (m, 4H), 6.92 (d, *J* = 7.7 Hz, 1H), 6.82–6.56 (m, 1H), 4.45–4.17 (m, 1H), 3.80–3.71 (m, 3H), 3.71–3.58 (m, 2H), 3.34–3.28 (m, 1H), 3.24 (dd, *J* = 14.3, 3.7 Hz, 1H), 3.19–3.08 (m, 1H), 3.04 (dd, *J* = 14.5, 7.8 Hz, 1H), 2.73–2.61 (m, 2H), 2.54–2.36 (m, 3H), 1.85–1.61 (m, 3H), 1.53–1.35 (m, 9H), 1.15–1.05 (m, 1H), 0.99 (t, *J* = 7.4 Hz, 2.4H), 0.96–0.84 (m, 1H), 0.71 (t, *J* = 7.3 Hz, 0.2H), 0.63 (t, *J* = 7.4 Hz, 0.4H). ^13^C NMR (101 MHz, methanol-*d*_4_) for a major rotamer: δ 173.3, 169.0, 161.2, 149.9, 142.9, 140.1, 138.3, 135.8, 131.1, 130.5, 130.3, 130.2, 129.6, 129.5, 127. 4, 126.9, 125.3, 119.3, 113.2, 112.5, 74.6, 56.8, 55.9, 55.6, 47.2, 46.8, 37.3, 36.5, 31.8, 30.1, 29.1, 28.9, 26.1, 21.5, 11.7. HRMS-ESI (*m*/*z*) calcd for C_36_H_48_N_3_O_4_ [M + H]^+^ 586.3645 Found 586.3651. [α]_D_^20^ = −9.5 (*c* 0.8, CHCl_3_)

## Supporting Information Available

Synthesis of building blocks **23a**–**d**, **26a**–**d**, and **38a,b;**^1^H NMR, ^13^C NMR spectra of products **7a–k**; and HPLC analysis of final products **7a**–**k** (PDF)
The PMX receptor (7TBC) and the docked ligands (**6a**) (PDB)
The PMX receptor (7TBC) and the docked ligands (**7k**) (PDB)
Molecular formula strings and the associated biochemical data (CSV)

## Supplementary Material

jm3c00812_si_001.pdf

jm3c00812_si_002.pdb

jm3c00812_si_003.pdb

## Abbreviations

CL_int_: intrinsic clearance
DoR: Day of Recrudescence
GSK: GlaxoSmithKline
ND: not determined
PPB: plasma protein binding
RBC: red blood cell
