# Supplementary material for: Macrocyclic Peptidomimetic Plasmepsin X Inhibitors with Potent In Vitro and In Vivo Antimalarial Activity
Source: J Med Chem. 2023 Jul 28;66(15):10658–80. doi: 10.1021/acs.jmedchem.3c00812 (PMC10424242; doi:10.1021/acs.jmedchem.3c00812)
Supplement: Supplementary file 1 — jm3c00812_si_001.pdf [file jm3c00812_si_001.pdf]

Supporting Information

Macrocyclic peptidomimetic plasmepsin X inhibitors with potent *in vitro* and *in vivo* antimalarial activity

Vadims Kovada,[a] Chrislaine Withers-Martinez,[b] Raitis Bobrovs,[a] Helēna Cīrule,[a] Edgars Liepins,[a] Solveiga Grinberga,[a] Fiona Hackett,[b] Christine R. Collins,[b] Agrita Kreicberga,[a] María Belén Jiménez-Díaz, [c] Iñigo Angulo-Barturen,[c] Dace Rasina,[a] Edgars Suna,[a] Kristaps Jaudzems, [a] Michael J. Blackman,[b,d]* and Aigars Jirgensons[a]*

[a]Latvian Institute of Organic Synthesis, Riga LV-1006, Latvia;

[b]Malaria Biochemistry Laboratory, The Francis Crick Institute, London NW1 1AT, United Kingdom

[c]The Art of Discovery SL, Biscay Science and Technology Park, Derio 48160, Bizkaia, Basque Country, Spain

[d]Faculty of Infectious and Tropical Diseases, London School of Hygiene & Tropical Medicine, London WC1E 7HT, United Kingdom

e-mail: aigars@osi.lv; mike.blackman@crick.ac.uk

Table of contents

Synthesis of building blocks **23a-d** S2
Synthesis of building blocks **26a-d** S3
Synthesis of building blocks **38a,b** S5
^{1}H NMR and ^{13}C NMR spectra of products **7a–k** S7
HPLC analysis of final products **7a-k** S27
Therapeutic efficacy of inhibitor **7f** against *P. falciparum in vivo* S38

Synthesis of building blocks 23a-d

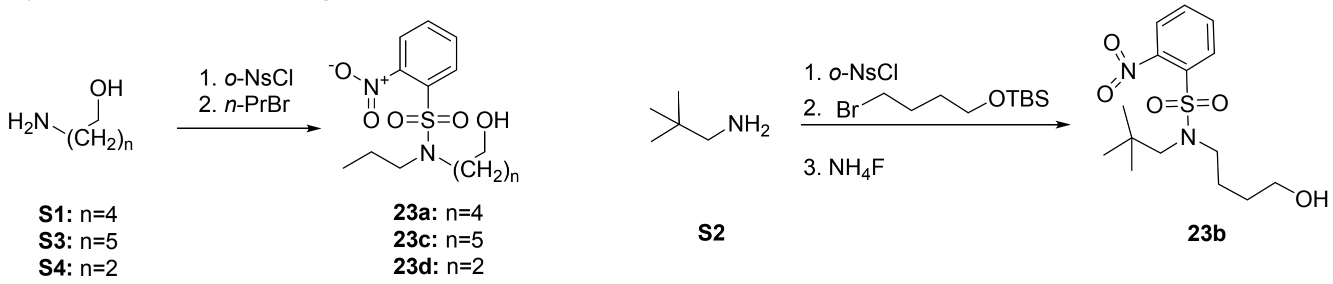

General procedure for the synthesis of nosylated hydroxyamines

To an ice bath cooled solution of *o*-nitrobenzenesulfonyl chloride (1 equiv) in DCM (1.1 mL/1 mmol of the sulfonyl chloride) was added a solution of aminoalcohol (2 equiv) and triethylamine (3 equiv) in DCM (1.1 mL/1 mmol of the sulfonyl chloride). Ice bath was removed and the reaction mixture was allowed to warm to room temperature. After stirring at room temperature for 2 h, the reaction mixture was quenched with an aqueous 0.1M solution of HCl (20 mL). Phases were separated and the aqueous phase was extracted with DCM (3 x 15mL). The combined organic layers were washed with brine and dried over Na_2SO_4. All volatiles were removed under reduced pressure.

The crude protected aminoalcohol from previous step was dissolved in anhydrous DMF (4.4 mL/ 1 mmol of the sulfonyl chloride). 1-Bromopropane (3 equiv) was added, followed by K_2CO_3 (6 equiv). The reaction mixture was stirred at room temperature overnight, quenched with water (30 mL) and extracted with DCM (3x30 mL). The combined organic phase was washed with brine, dried over anhydrous Na_2SO_4, and evaporated under reduced pressure.

*N-(4-Hydroxybutyl)-2-nitro-N-propylbenzenesulfonamide (**23a**).* The title compound was obtained as a light yellow oil (1.32 g, 93% yield) from 4-aminobutan-1-ol (**S1**) (803 mg, 9.00 mmol, 2 equiv), triethylamine (1.88 mL, 13.5 mmol, 3 equiv), *o*-nitrobenzenesulfonyl chloride (1.00 g, 4.51 mmol, 1 equiv), *n*-propyl bromide (1.23 mL, 13.5 mmol, 3 equiv) and K_2CO_3 (3.7 g, 27.0 mmol, 6 equiv), following general procedure. Pure material was obtained by column chromatography on silica gel using 50% EtOAc in hexanes. ^{1}H NMR (400 MHz, Chloroform-*d*): δ 8.02 – 7.95 (m, 1H), 7.70 – 7.63 (m, 2H), 7.63 – 7.56 (m, 1H), 3.62 (t, *J* = 6.2 Hz, 2H), 3.35 – 3.28 (m, 2H), 3.27 – 3.20 (m, 2H), 1.69 (br s, 1H), 1.68 – 1.58 (m, 2H), 1.58 – 1.47 (m, 4H), 0.83 (t, *J* = 7.4 Hz, 3H). ^{13}C NMR (101 MHz, Chloroform-*d*): δ 148.1, 133.8, 133.5, 131.7, 130.7, 124.2, 62.3, 49.0, 47.2, 29.6, 24.8, 21.5, 11.1. HRMS-ESI (m/z) calcd. for $C_{13}H_{21}N_2O_5S$ $[M+H]^+$ 317.1171. Found 317.1172.

*N-(4-Hydroxybutyl)-N-neopentyl-2-nitrobenzenesulfonamide (**23b**).* To an ice bath cooled solution of *o*-nitrobenzenesulfonyl chloride (500 mg, 2.26 mmol, 1 equiv) in DCM (5 mL) was added a solution of *neo*-pentyl amine (**S2**) (0.53 mL, 4.51 mmol, 2 equiv) and triethylamine (0.94 mL, 6.77 mmol, 3 equiv) in DCM (5 mL). Ice bath was removed and the reaction mixture was allowed to warm to room temperature. After stirring at room temperature for 2 h, the reaction mixture was quenched with a 0.1 M HCl solution (20 mL). Phases were separated and the aqueous phase was extracted with DCM (3 x 15mL). The combined organic phase was washed with brine and dried over Na_2SO_4. All volatiles were removed under reduced pressure. The crude amine from previeous step was dissolved in anhydrous DMF (10 mL). (4-Bromobutoxy)(tert-butyl)dimethylsilane (1.12 g, 4.19 mmol, 2 equiv) was added, followed by NaOH (167 mg, 4.19 mmol, 2 equiv). The reaction mixture was stirred overnight at room temperature then quenched with water (30 mL) and extracted with diethylether (3x20 mL). The combined organic phase was washed with brine and dried over Na_2SO_4. All volatiles were

removed under reduced pressure. The residue was dissolved in MeOH (40 mL). Ammonium fluoride (1.16 g, 31.4 mmol, 15 equiv) was added and the reaction mixture was stirred at room temperature for 100 hours. Silica gel (5 g) was added and methanol was evaporated under reduced pressure. The residue was added to silica gel column and eluted with 30% EtOAc in hexanes. Collected product was repurified by column chromatography on silica gel using 2% methanol in DCM. Product **23b** was collected as a yellowish oil (320 mg, 44% yield). ^{1}H NMR (400 MHz, Chloroform-*d*): δ 8.04 – 8.01 (m, 1H), 7.71 – 7.64 (m, 2H), 7.62 – 7.59 (m, 1H), 3.55 (t, *J* = 6.3 Hz, 2H), 3.41 – 3.35 (m, 2H), 3.18 (s, 2H), 1.64 – 1.54 (m, 2H), 1.45 – 1.35 (m, 2H), 0.96 (s, 9H). ^{13}C NMR (101 MHz, Chloroform-d): δ 148.3, 133.9, 133.6, 131.6, 131.2, 124.2, 62.38, 58.7, 49.3, 33.6, 29,8, 28.5, 23.8. HRMS-ESI (m/z) calcd. for $C_{15}H_{25}N_2O_5S$ $[M+H]^+$ 345.1484. Found 345.1484.

*N-(5-hydroxypentyl)-2-nitro-N-propylbenzenesulfonamide (**23c**).* The title compound was obtained as a light yellow oil (1.31 mg, 87% yield) from 5-aminopentan-1-ol (**S3**) (931 mg, 9.02 mmol, 2.0 equiv), triethylamine (1.89 mL, 13.5 mmol, 3 equiv), *o*-nitrobenzenesulfonyl chloride (1.00 g, 4.51 mmol, 1 equiv), *n*-propyl bromide (1.24 mL, 13.6 mmol, 3 equiv), and K_2CO_3 (3.77 g, 27.3 mmol, 6 equiv), following general procedure. Pure material was obtained by column chromatography on silica gel using 50% EtOAc in hexanes. ^{1}H NMR (400 MHz, Chloroform-*d*): δ 8.04 – 7.97 (m, 1H), 7.71 – 7.64 (m, 2H), 7.63 – 7.57 (m, 1H), 3.61 (td, *J* = 6.4, 0.9 Hz, 2H), 3.33 – 3.26 (m, 2H), 3.26 – 3.20 (m, 2H), 1.64 – 1.49 (m, 6H), 1.49 (br s, 1H), 1.39 – 1.29 (m, 2H), 0.85 (t, *J* = 7.3 Hz, 3H). ^{13}C NMR (101 MHz, Chloroform-*d*) δ 148.1, 134.0, 133.4, 131.6, 130.8, 124.2, 62.7, 49.1, 47.3, 32.3, 28.1, 22.9, 21.5, 11.2. HRMS-ESI (m/z) calcd. for $C_{14}H_{23}N_2O_5S$ $[M+H]^+$ 331.1328. Found 331.1335.

*N-(2-hydroxyethyl)-2-nitro-N-propylbenzenesulfonamide (**23d**).* The title compound was obtained as a light yellow oil (607 mg, 93% yield) from 2-aminoethan-1-ol (**S4**) (276 mg, 4.51 mmol, 2 equiv), triethylamine (0.94 mL, 6.77 mmol, 3 equiv), *o*-nitrobenzenesulfonyl chloride (500 mg, 2.26 mmol, 1 equiv), *n*-propyl bromide (0.62 mL, 6.77 mmol, 3 equiv), and K_2CO_3 (1.87 g, 13.5 mmol, 6 equiv), following general procedure. Pure material was obtained by column chromatography on silica gel using 50% EtOAc in hexanes. ^{1}H NMR (400 MHz, Chloroform-*d*): δ 8.04 – 7.97 (m, 1H), 7.72 – 7.64 (m, 2H), 7.64 – 7.57 (m, 1H), 3.71 (t, *J* = 5.6 Hz, 2H), 3.44 (t, *J* = 5.6 Hz, 2H), 3.33 – 3.24 (m, 2H), 1.63 – 1.50 (m, 2H), 0.83 (t, *J* = 7.4 Hz, 3H). ^{13}C NMR (101 MHz, Chloroform-*d*): δ 148.1, 133.7, 133.2, 131.9, 130.8, 124.2, 60.5, 50.2, 49.5, 21.5, 11.0. HRMS-ESI (m/z) calcd. for $C_{11}H_{17}N_2O_5S$ $[M+H]^+$ 289.0858. Found 289.0862.

Synthesis of building blocks 26a-d

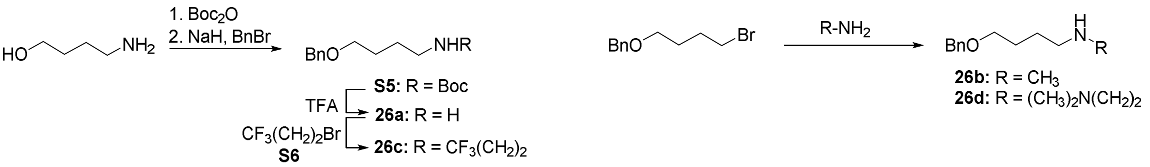

*Tert-butyl (4-(benzyloxy)butyl)carbamate (**S5**).* To a cooled 0°C solution of 4-amino-1-butanol (3.00 g, 33.7 mmol, 1 equiv) in DCM (25 mL) was added a solution of Boc_2O (7.34 g, 33.7 mmol, 1 equiv) in DCM (5 mL). The reaction mixture was stirred at room temperature for 2 h. Solvent was evaporated uneder redused pressure. The residue was dissolved in DMF (33 mL) and cooled to 0°C. NaH (60% in mineral oil) (2.69 g, 67.3 mmol, 2 equiv) and the reaction mixture was stirred at 0°C for 30 min before benzyl bromide (6.00 mL, 50.5 mmol, 1.5 equiv) was added . The reaction mixture was warmed to room temperature and stirred overnight. The

reaction was cooled to 0°C and quenched with water and extracted with EtOAc (3 x 50 mL). Combined organic phase was washed with brine, dried over anhydrous Na_2SO_4, and evaporated under reduced pressure. Purification by column chromatography on silica gel using gradient elution from 5% to 30% EtOAc in hexanes afforded product **S5** as a colorless oil (7.28 mg, 77% yield). ^{1}H NMR (400 MHz, Chloroform-*d*): δ 7.38 – 7.30 (m, 4H), 7.30 – 7.23 (m, 1H), 4.64 (br s, 1H), 4.50 (s, 2H), 3.48 (t, *J* = 6.1 Hz, 2H), 3.14 (q, *J* = 6.5 Hz, 2H), 1.70 – 1.53 (m, 4H), 1.44 (s, 9H). ^{13}C NMR (101 MHz, Chloroform-*d*): δ 156.1, 138.6, 128.5, 127.7, 127.7, 79.1, 73.1, 70.1, 40.5, 28.5, 27.2, 27.0.

*4-(Benzyloxy)butan-1-amine (**26a**).* To a cooled solution of compound **S5** (7.27 g, 26.0 mmol, 1 equiv) trifluoroacetic acid (30.0 mL, 390 mmol, 15 equiv) was added, and the reaction mixture was stirred at 0°C for 1 h before volatiles were evaporated. The residue was neutralised with sat. aq. $NaHCO_3$, extracted with EtOAc (3 x 50 mL), washed with brine, dried over anhydrous Na_2SO_4, and evaporated under reduced pressure, giving product **26a** as yellowish oil (4.43 g, 95% yield), which was used without further purification. ^{1}H NMR (400 MHz, Chloroform-*d*): δ 7.38 – 7.31 (m, 4H), 7.31 – 7.25 (m, 1H), 4.50 (s, 2H), 3.49 (t, *J* = 6.4 Hz, 2H), 2.71 (t, *J* = 7.0 Hz, 2H), 1.70 – 1.62 (m, 2H), 1.57 – 1.48 (m, 2H). ^{13}C NMR (101 MHz, Chloroform-*d*) δ 138.7, 128.5, 127.75, 127.65, 73.1, 70.4, 42.2, 30.7, 27.3.

*4-(Benzyloxy)-N-methylbutan-1-amine (**26b**).* 4-(Benzyloxy)-1-bromobutane (165 µL, 0.87 mmol, 1 equiv) was added to a 33% solution of methyl amine in EtOH (5.40 mL, 43.4 mmol, 50 equiv). The reaction mixture was stirred at RT for 4 h. Volatiles were evaporated under reduced pressure. The residue was dissolved in diethyl ether (20 mL), washed with aqueous 1M NaOH, brine, dried over anhydrous Na_2SO_4, and evaporated under reduced pressure. Crude amine **26b** (145 mg, 87% yield) was used in the next step without purification. ^{1}H NMR (300 MHz, Chloroform-*d*): δ 7.41 – 7.27 (m, 5H), 4.50 (s, 2H), 3.56 (t, *J* = 5.8 Hz, 2H), 2.95 (t, *J* = 7.2 Hz, 2H), 2.48 (s, 3H), 1.99 (p, *J* = 7.2 Hz, 2H), 1.82 – 1.70 (m, 2H).

*4-(Benzyloxy)-N-(3,3,3-trifluoropropyl)butan-1-amine (**26c**).* To a solution of 3-bromo-1,1,1-trifluoropropane (**S6**) (230 mg, 1.30 mmol, 1 equiv) in MeCN (12 mL) was added amine **26a** (1.17 g, 6.5 mmol, 5 equiv). The reaction mixture was stirred at room temperature overnight. Volatiles were evaporated under reduced pressure. The residue was dissolved in DCM (20 mL), washed with aqueous 1M NaOH (20 mL). Aqueous phase was separated and extracted with DCM (2x20 mL). Combined organic phase was washed with brine (20 mL), dried over anhydrous Na_2SO_4, and evaporated under reduced pressure. Purification by column chromatography on silica gel using DCM:MeOH:triethylamine (100:5:1 v/v/v) afforded amine **26c** as a colorless oil (170 mg, 48% yield), as well as unreacted excess of amine **26a**. ^{1}H NMR (400 MHz, Chloroform-*d*): δ 7.38 – 7.27 (m, 5H), 4.50 (s, 2H), 3.49 (t, *J* = 6.2 Hz, 2H), 2.85 (dd, *J* = 7.6, 6.9 Hz, 2H), 2.64 (t, *J* = 6.9 Hz, 2H), 2.27 (qdd, *J* = 11.0, 7.6, 6.9 Hz, 2H), 1.70 – 1.51 (m, 4H). ^{13}C NMR (101 MHz, Chloroform-*d*): δ 138.6, 128.5, 127.8, 127.7, 126.8 (q, *J* = 276.5 Hz), 73.1, 70.3, 49.7, 42.7 (q, *J* = 3.4 Hz), 34.4 (q, *J* = 27.5 Hz), 27.6, 26.9.

*N^1-(4-(Benzyloxy)butyl)-N^2,N^2-dimethylethane-1,2-diamine (**26d**).* To a solution of 4-(benzyloxy)-1-bromobutane (2.56 g, 10.5 mmol, 1 equiv) in MeCN (100 mL) was added *N^1,N^1*-dimethylethane-1,2-diamine (5.80 mL, 52.6 mmol, 5 equiv). The reaction mixture was stirred at room temperature overnight. Volatiles were evaporated under reduced pressure. The residue was dissolved in DCM (40 mL), washed with aqueous 1M NaOH (40 mL). Aqueous phase was separated and extracted with DCM (2 x 40 mL). Combined organic phase was washed with brine (20 mL), dried over anhydrous Na_2SO_4, and evaporated under reduced pressure. Crude amine

26d (1.8 g, 68% yield) was used in the next step without purification. ^{1}H NMR (300 MHz, Chloroform-*d*): δ 7.38 – 7.23 (m, 5H), 4.50 (s, 2H), 3.52 – 3.45 (m, 2H), 2.70 – 2.60 (m, 4H), 2.39 (t, *J* = 6.2 Hz, 2H), 2.21 (s, 6H), 1.72 – 1.51 (m, 4H).

Synthesis of building blocks 38a,b

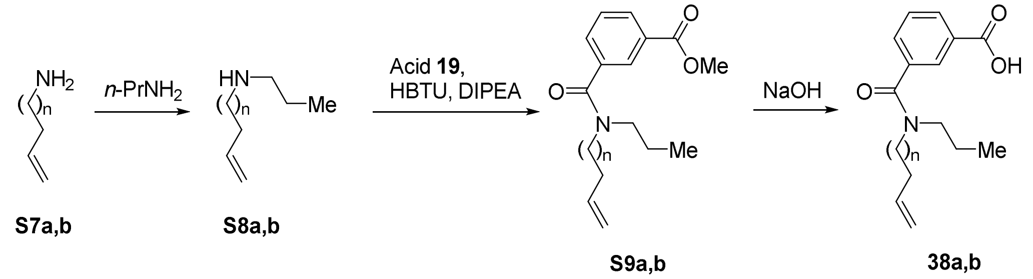

S7a-S9a, 38a: n = 1
S7b-S9b, 38b: n = 2

General procedure for the synthesis of amides

Solution of carboxylic acid (1-2 equiv) in anhydrous DCM (1 mL/ 0.1 mmol of the acid) was cooled to 0°C. DIPEA (1-4 eqiuv) was added, followed by HBTU (1-2 equiv). The reaction mixture was stirred at 0°C for 10 minutes. A solution of the amine (1-2 equiv) in DCM (1 mL/ 0.2 mmol) was added, and the reaction mixture was allowed to warm up to room temperature and stirred for 30 minutes. Solvent was evaporated under reduced pressure. The residue was dissolved in EtOAc (40 mL), and the solution was washed with 1M HCl (40 mL), 1M NaOH (40 mL), brine (40 mL), dried over anhydrous Na_2SO_4, and evaporated under reduced pressure.

General procedure for the hydrolysis of methylbenzoates

An aqueous 1M solution of NaOH (2-6 equiv) was added to a solution of benzoate (1.0 equiv) in THF (2.5 mL/0.1 mmol of the benzoate). The resulting solution was stirred until conversion of starting material is complete. Solvent was evaporated under reduced pressure.

*Methyl 3-(but-3-en-1-yl(propyl)carbamoyl)benzoate (**S9a**).* 4-Bromobut-1-ene (**S7a**) (526 μL, 5.19 mmol, 1 equiv) was added to *n*-propylamine (12.8 mL, 155 mmol, 30 equiv) dropwise at room temperature. The mixture was stirred at room temperature overnight. Volatiles were evaporated under reduced pressure, giving crude amine **S8a** (contains 357 mg of **S8a,** 61% yield), which was used in the next step without further purification. The title compound was obtained as colorless oil (373 mg, 43% yield) from acid **19** (1.14 g, 6.31 mmol, 2 equiv), HBTU (2.39 g, 6.31 mmol, 2 equiv), DIPEA (2.18 mL, 12.6 mmol, 4 equiv) and crude amine from previous step (357 mg, 3.15 mmol, 1 equiv), following general procedure for the synthesis of amides. Pure material was obtained by column chromatography on silica gel using gradient elution from DCM to 4% MeOH in DCM. ^{1}H NMR (400 MHz, Methanol-d_4) for 2 rotamers: δ 8.13 – 8.08 (m, 1H), 8.01 – 7.96 (m, 1H), 7.64 – 7.56 (m, 2H), 5.99 – 5.83 (m, 0.5H), 5.66 – 5.50 (m, 0.5H), 5.24 – 5.06 (m, 1H), 5.05 – 4.94 (m, 1H), 3.93 (s, 3H), 3.60 (t, *J* = 7.2 Hz, 1H), 3.50 (t, *J* = 7.6 Hz, 1H), 3.34 – 3.32 (m, 1H), 3.20 (t, *J* = 7.6 Hz, 1H), 2.47 (q, *J* = 7.2 Hz, 1H), 2.28 (q, *J* = 7.5 Hz, 1H), 1.74 (h, *J* = 7.2 Hz, 1H), 1.57 (h, *J* = 7.3 Hz, 1H), 1.01 (t, *J* = 7.4 Hz, 1.3H), 0.74 (t, *J* = 7.4 Hz, 1.7H). ^{13}C NMR (101 MHz, Methanol-d_4) for 2 rotamers: δ 173.0, 167.6, 138.5, 136.7, 135.6, 132.2, 132.1, 131.8, 131.4, 130.1, 128.6, 128.5, 118.0, 117.3, 52.9, 52.3, 50.0, 48.0, 45.5, 34.0, 33.0, 22.8, 21.6, 11.6, 11.2. HRMS-ESI (m/z) calcd. for $C_{16}H_{22}NO_3$ $[M+H]^+$ 276.1600. Found 276.1610.

*Methyl 3-(pent-4-en-1-yl(propyl)carbamoyl)benzoate (**S9b**).* 5-Bromopent-1-ene (**S7b**) (794 µL, 6.71 mmol, 1 equiv) was added to *n*-propylamine (16.5 mL, 201 mmol, 30 equiv) dropwise at room temperature. The mixture was stirred at room temperature overnight. Volatiles were evaporated under reduced pressure, giving crude amine **S8b** (contains 850 mg of **S8b,** 86% yield), which was used in the next step without further purification. The title compound was obtained as colorless oil (1.10 g, 66% yield) from acid **19** (1.04 g, 5.78 mmol, 1 equiv), HBTU (2.19 g, 5.78 mmol, 1 equiv), DIPEA (4.00 mL, 23.1 mmol, 4 equiv) and crude amine from previous step (735 mg, 5.78 mmol, 1 equiv), following general procedure for the synthesis of amides. Pure material was obtained by column chromatography on silica gel using 30% EtOAc in hexanes. ^{1}H NMR (600 MHz, Methanol-d_4) for 2 rotamers: δ 8.15 – 8.07 (m, 1H), 7.98 (s, 1H), 7.64 – 7.56 (m, 2H), 5.90 (dq, *J* = 16.8, 8.4, 7.6 Hz, 0.5H), 5.59 (dq, *J* = 16.7, 8.3, 7.5 Hz, 0.5H), 5.10 (d, *J* = 17.1 Hz, 1H), 5.01 (d, *J* = 10.2 Hz, 1H), 4.90 – 4.80 (m, 1H), 3.93 (s, 3H), 3.53 (t, *J* = 7.6 Hz, 1H), 3.49 (t, *J* = 7.7 Hz, 1H), 3.26 – 3.18 (m, 2H), 2.17 (q, *J* = 7.3 Hz, 1H), 1.88 (q, *J* = 7.2 Hz, 1H), 1.81 (p, *J* = 7.3 Hz, 1H), 1.73 (h, *J* = 7.2 Hz, 1H), 1.65 (p, *J* = 7.7 Hz, 1H), 1.57 (h, *J* = 7.5 Hz, 1H), 1.00 (t, *J* = 7.5 Hz, 2H), 0.74 (t, *J* = 7.4 Hz, 2H). ^{13}C NMR (151 MHz, Methanol-d_4) for 2 rotamers: δ 172.9, 167.6, 139.1, 138.5, 138.3, 132.1, 131.8, 131.4, 130.2, 128.41, 128.37, 115.8, 115.6, 52.9, 52.3, 50.0, 48.1, 46.0, 32.3, 31.6, 28.8, 27.7, 22.9, 21.7, 11.7, 11.2. HRMS-ESI (m/z) calcd. for $C_{17}H_{24}NO_3$ $[M+H]^+$ 290.1756. Found 290.1761.

3-(But-3-en-1-yl(propyl)carbamoyl)benzoic acid (38a). The hydrolysis of methyl benzoate was performed using compound **S9a** (180 mg, 0.654 mmol, 1 equiv), 1M solution of NaOH (1.3 mL, 1.3 mmol, 2 equiv), following general procedure for the hydrolysis of methyl benzoates. Full conversion was achieved after stirring at 60°C for 2 h. Crude acid was used in the next step without further purification.

*3-(Pent-4-en-1-yl(propyl)carbamoyl)benzoic acid (**38b**).* The hydrolysis of methyl benzoate was performed using compound **S9b** (250 mg, 0.865 mmol, 1 equiv), 1M solution of NaOH (1.73 mL, 1.73 mmol, 2 equiv), following general procedure for the hydrolysis of methyl benzoates. Full conversion was achieved after stirring at 60°C for 2 h. Crude acid was used in the next step without further purification. ^{1}H NMR (400 MHz, Methanol-d_4) for 2 rotamers: δ 8.11 (ddd, *J* = 6.4, 2.5, 1.7 Hz, 1H), 7.99 (q, *J* = 1.3 Hz, 1H), 7.63 – 7.54 (m, 2H), 6.00 – 5.83 (m, 0.5H), 5.67 – 5.53 (m, 0.5H), 5.14 – 4.98 (m, 1H), 3.51 (dt, *J* = 14.8, 7.5 Hz, 2H), 3.28 – 3.18 (m, 2H), 2.17 (q, *J* = 7.5 Hz, 1H), 1.95 – 1.85 (m, 1H), 1.87 – 1.75 (m, 1H), 1.76 – 1.69 (m, 1H), 1.69 – 1.61 (m, 1H), 1.57 (s, 1H), 1.00 (t, *J* = 7.4 Hz, 1.5H), 0.74 (t, *J* = 7.4 Hz, 1.5H).

^{1}H NMR and ^{13}C NMR spectra of products 7a–k

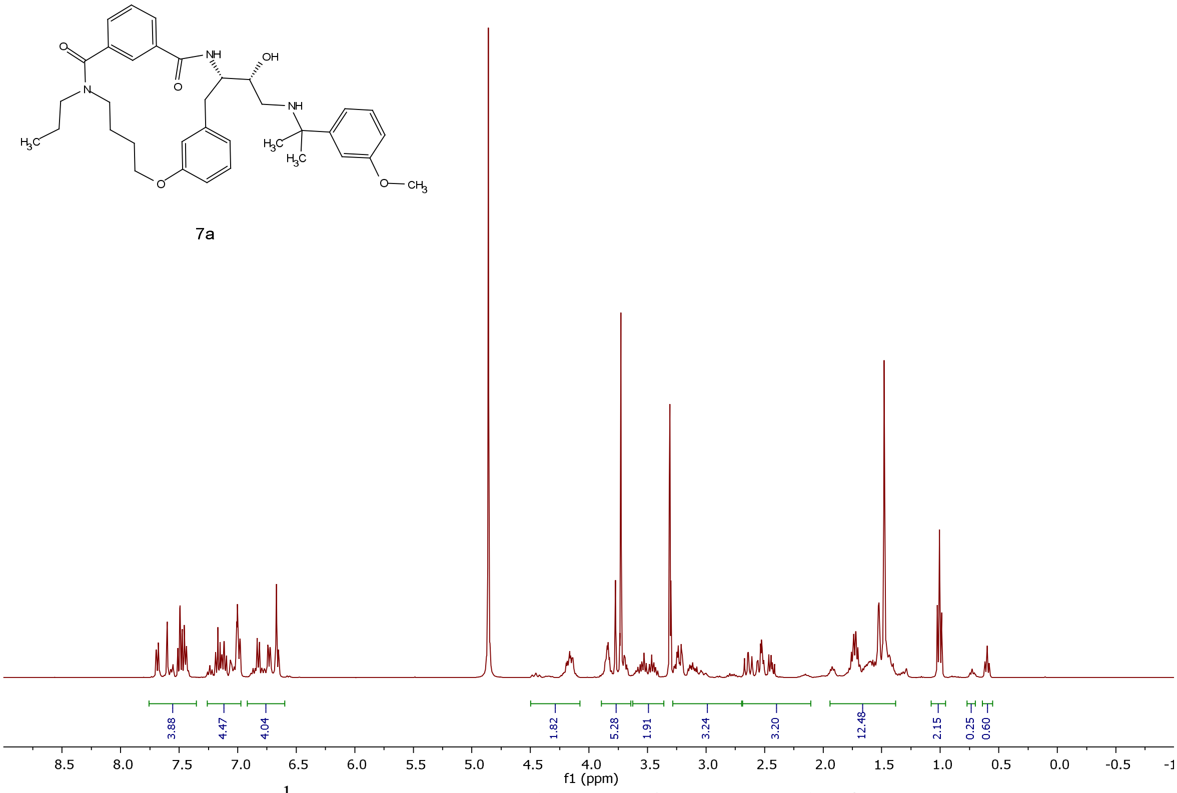

^{1}H NMR spectrum (MeOD-*d4*, 400 MHz) of **7a**

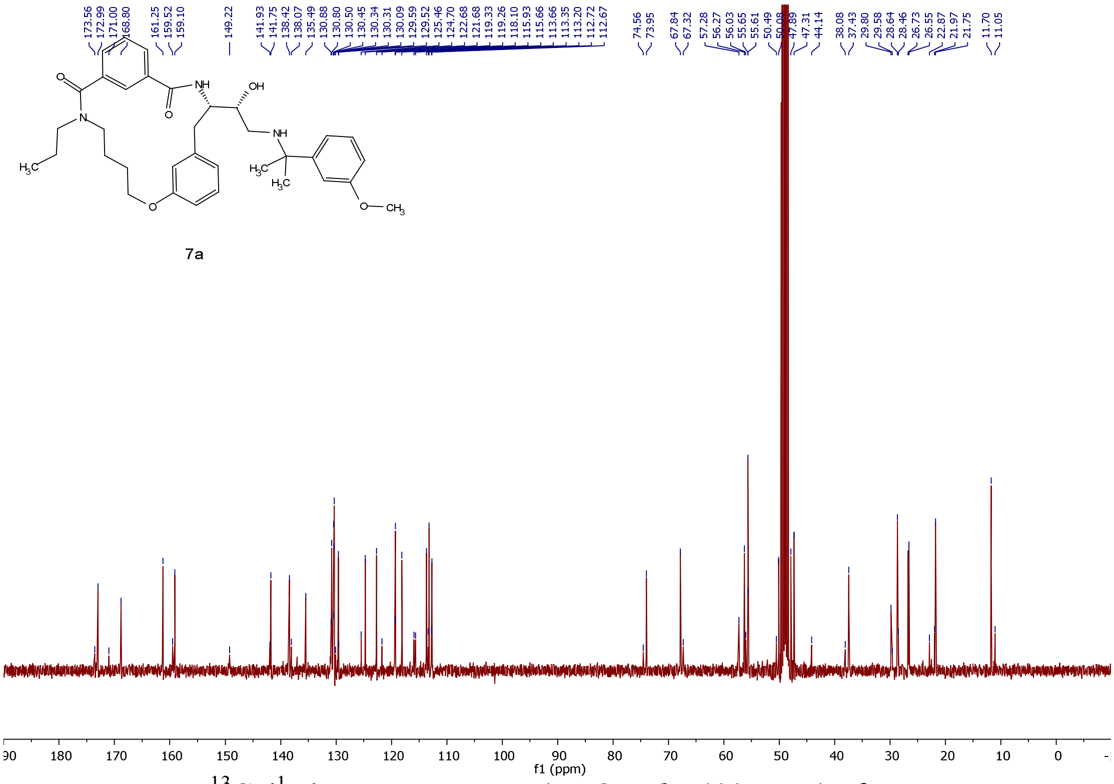

^{13}C {^{1}H} NMR spectrum (MeOD-*d4*, 400 MHz) of **7a**

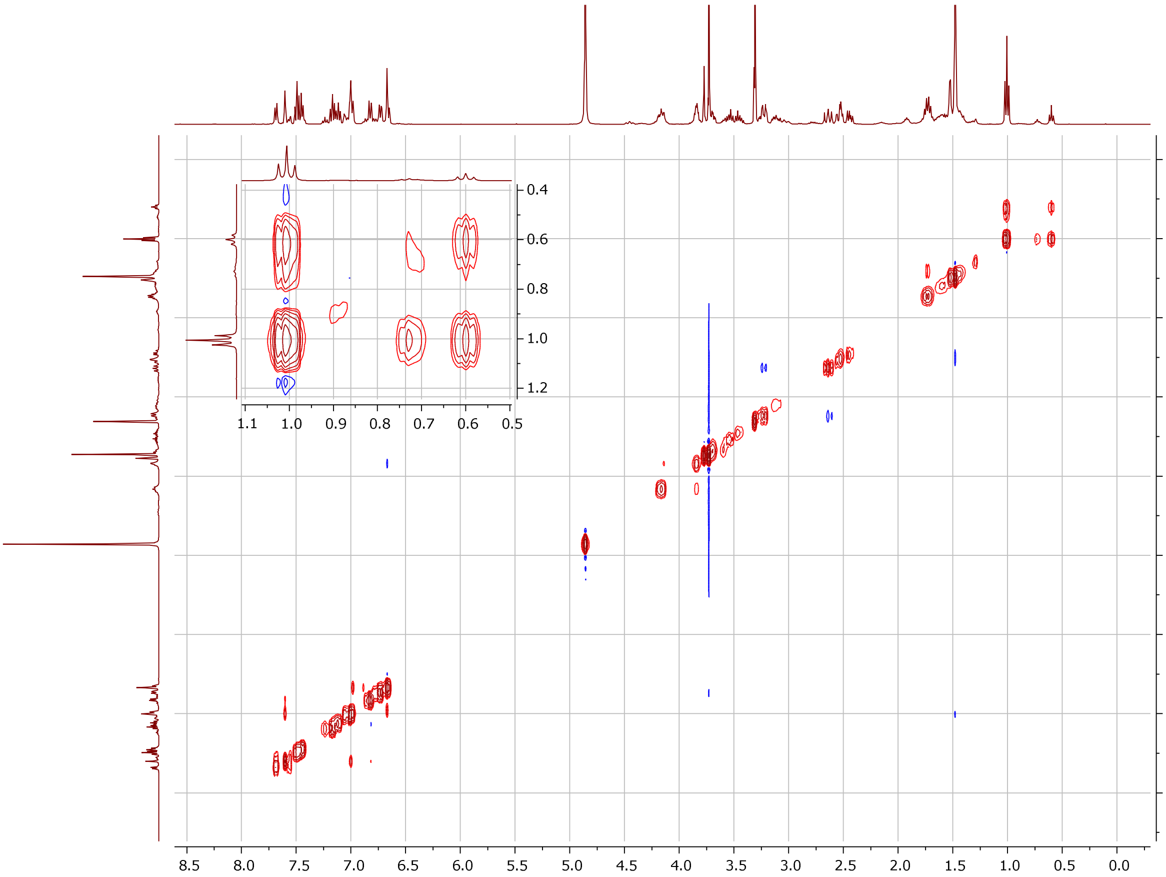

2D NOESY spectrum of compound **7a** (3 rotamers)

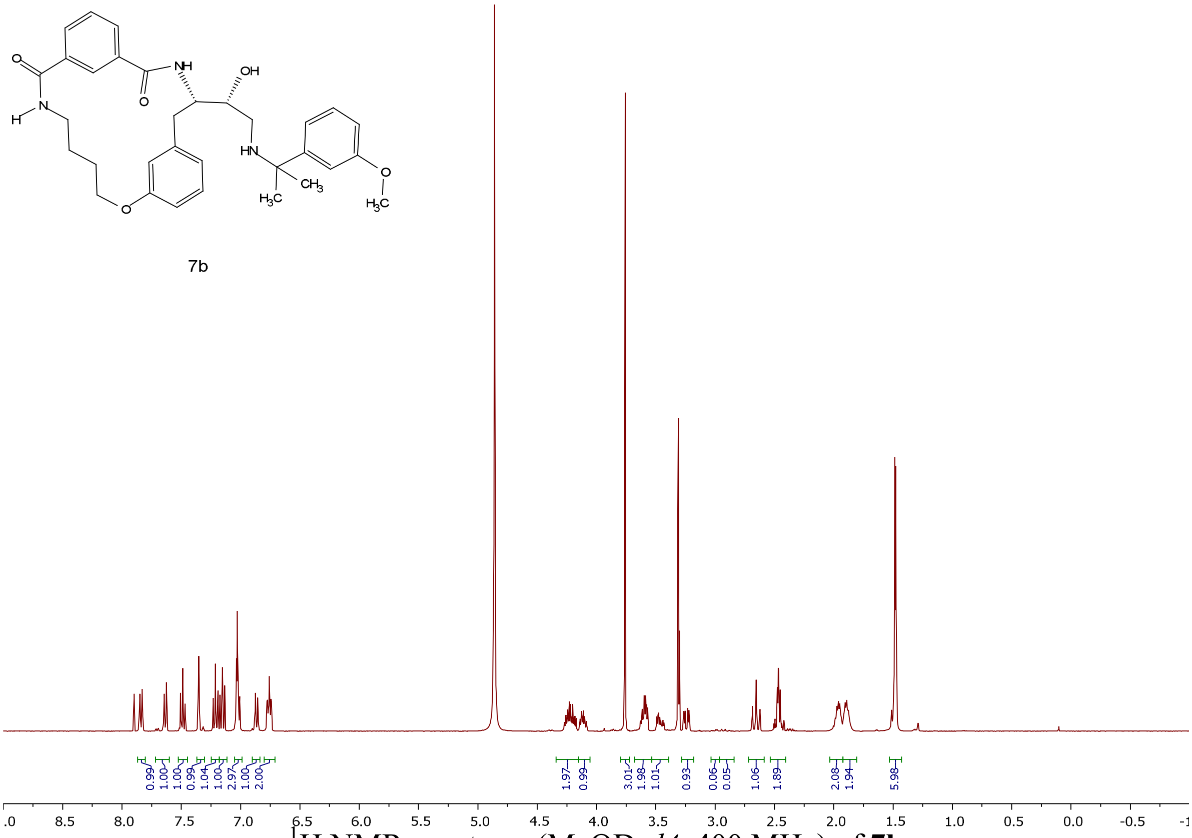

^{1}H NMR spectrum (MeOD-*d4*, 400 MHz) of **7b**

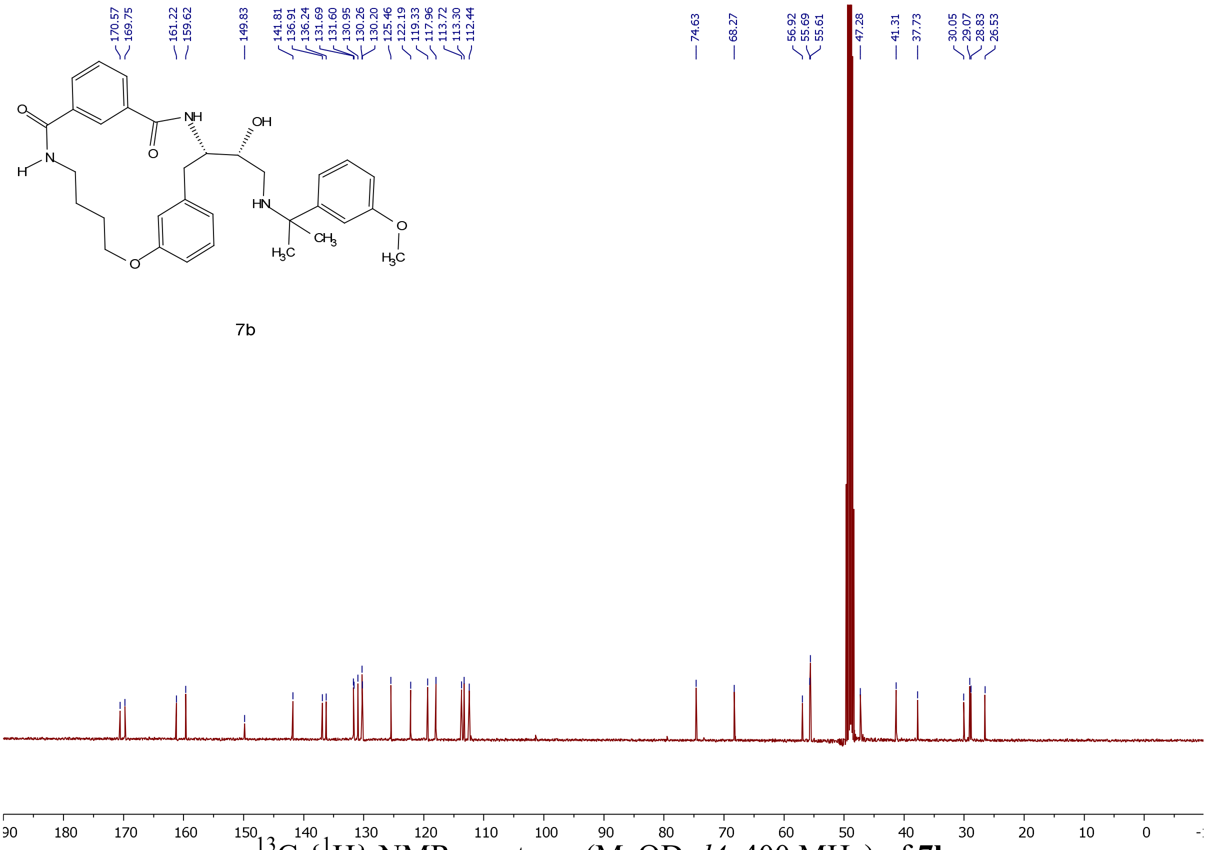

^{13}C {^{1}H} NMR spectrum (MeOD-*d4*, 400 MHz) of **7b**

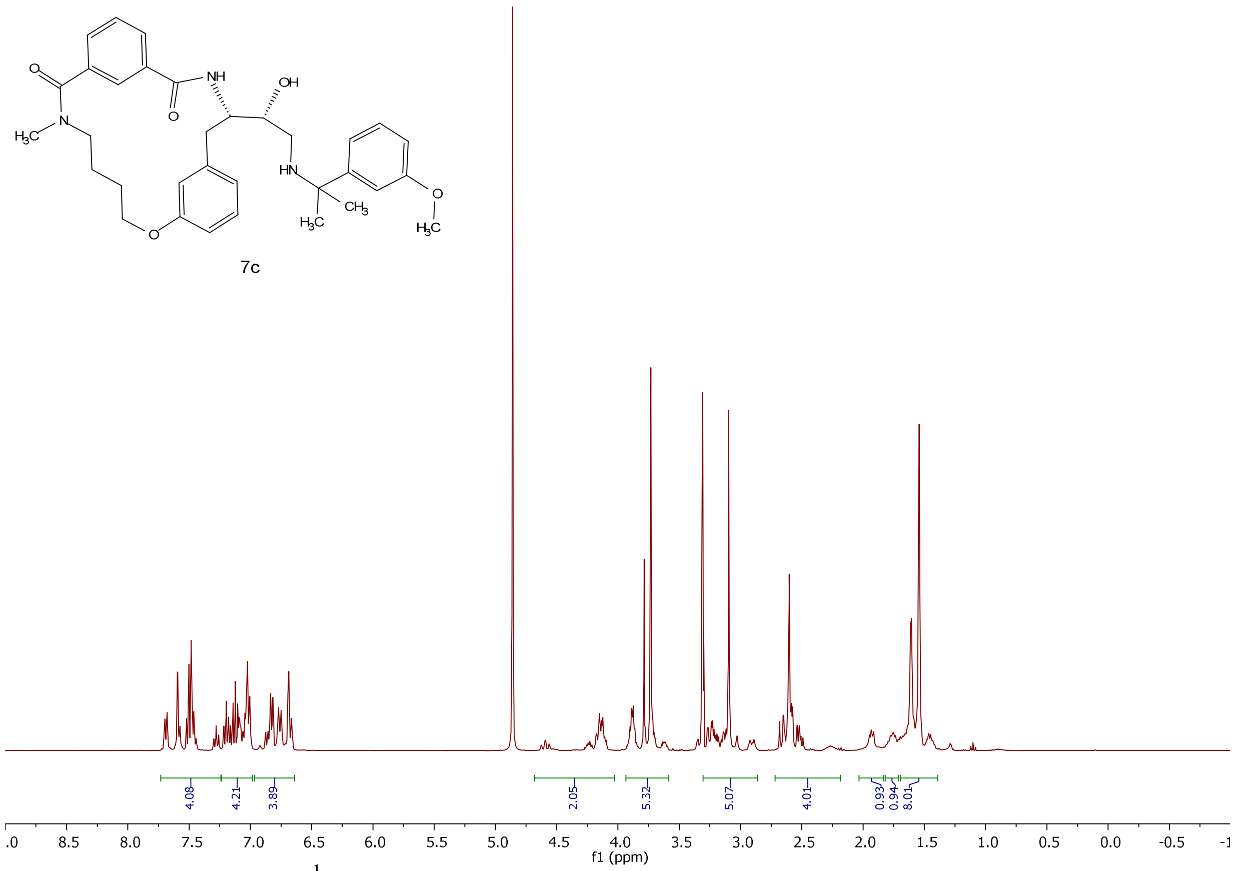

^{1}H NMR spectrum (MeOD-*d4*, 400 MHz) of **7c**

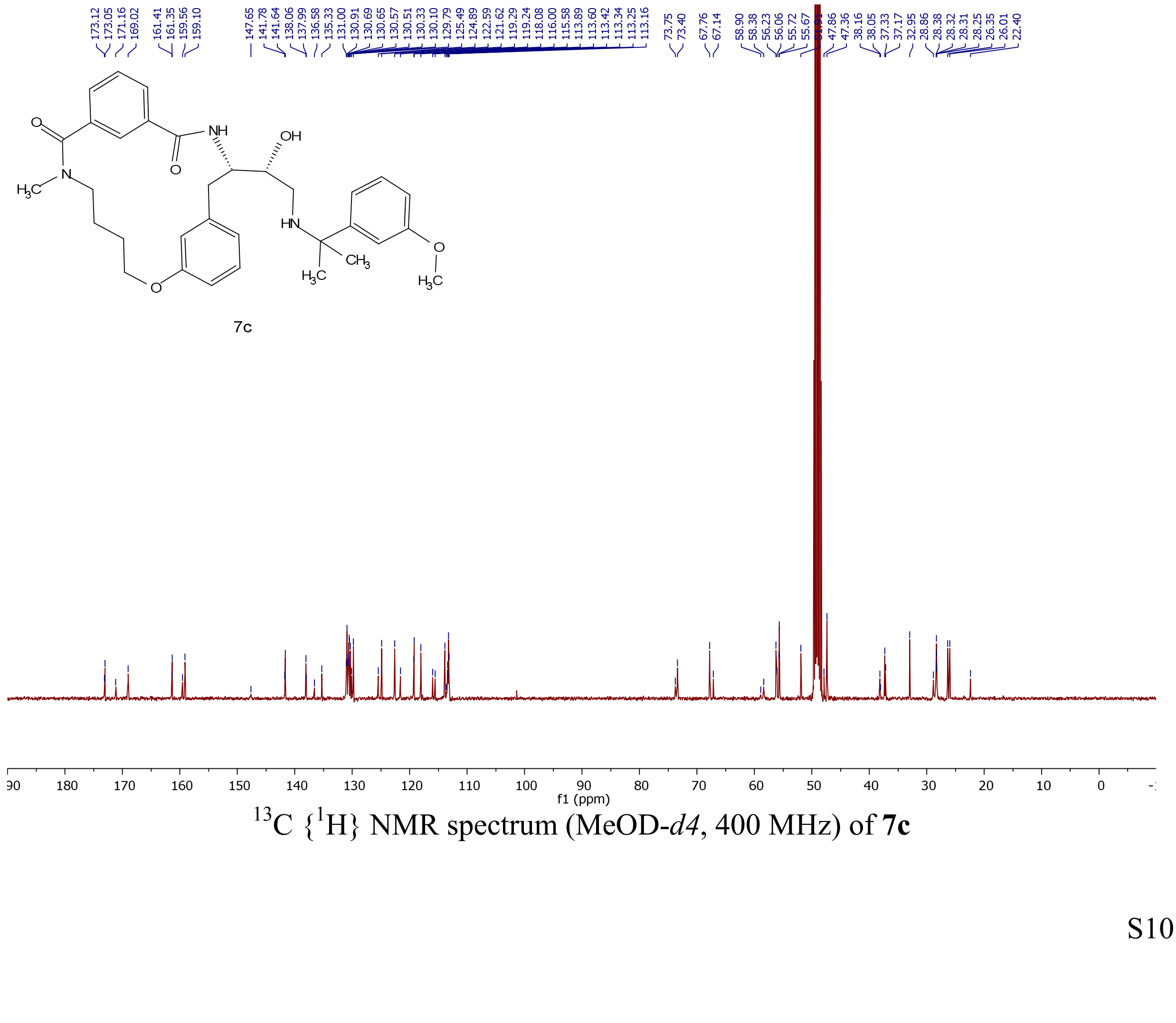

^{13}C {^{1}H} NMR spectrum (MeOD-*d4*, 400 MHz) of **7c**

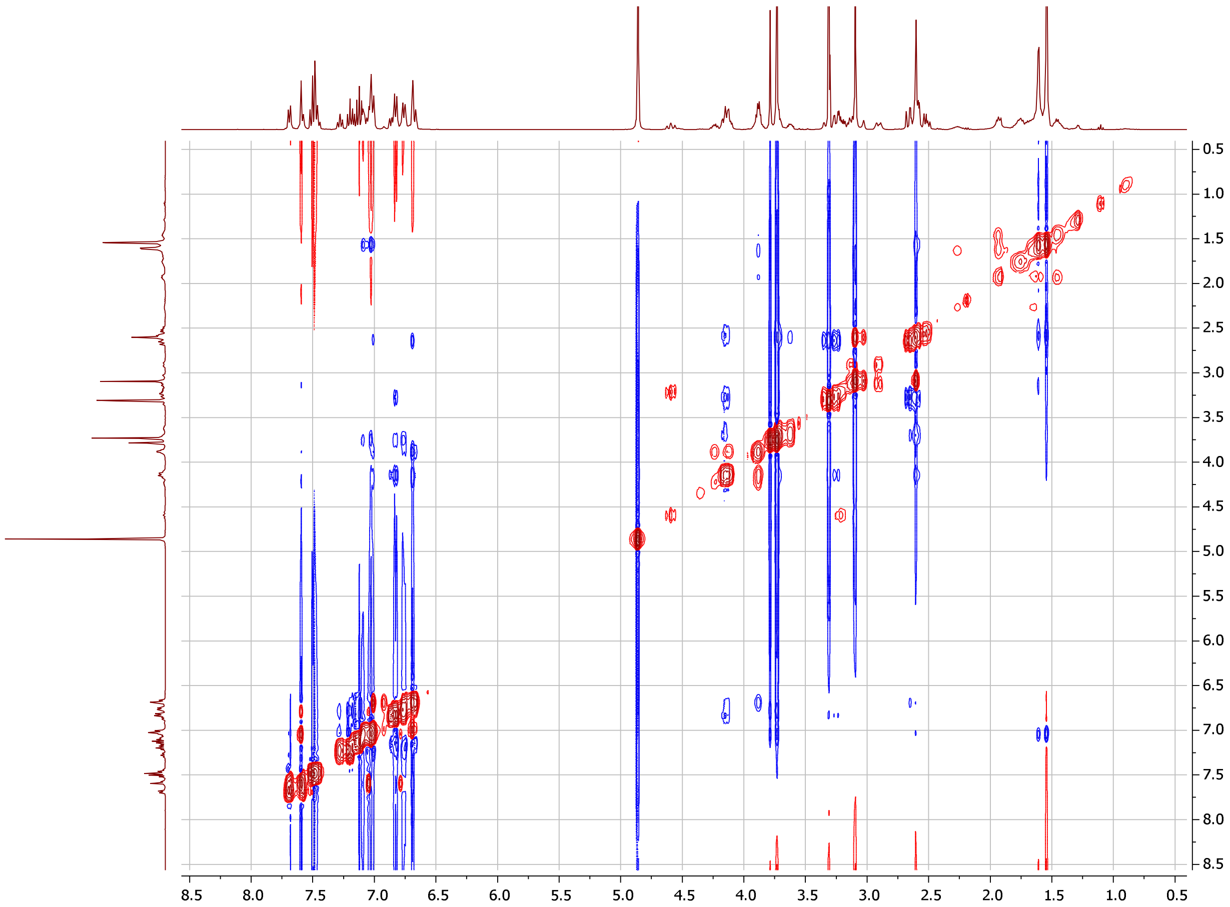

2D NOESY spectrum of compound **7c**

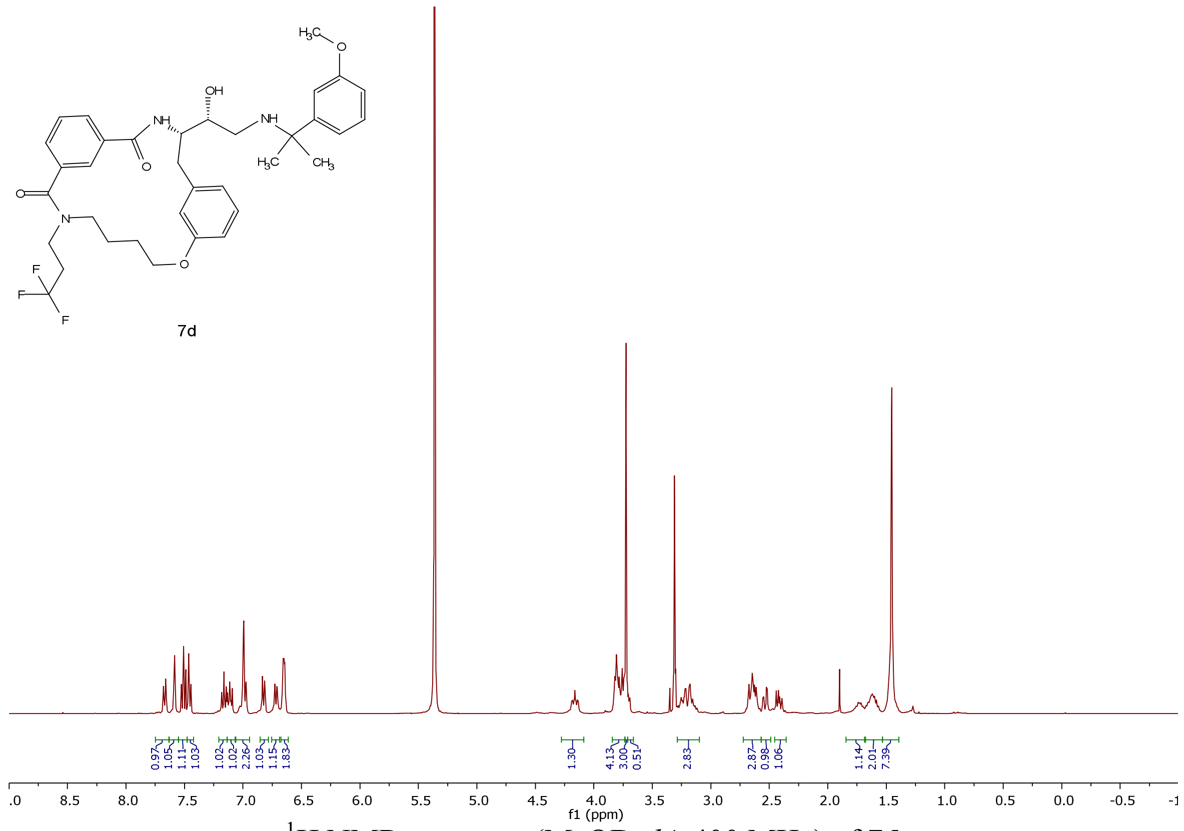

^{1}H NMR spectrum (MeOD-*d4*, 400 MHz) of **7d**

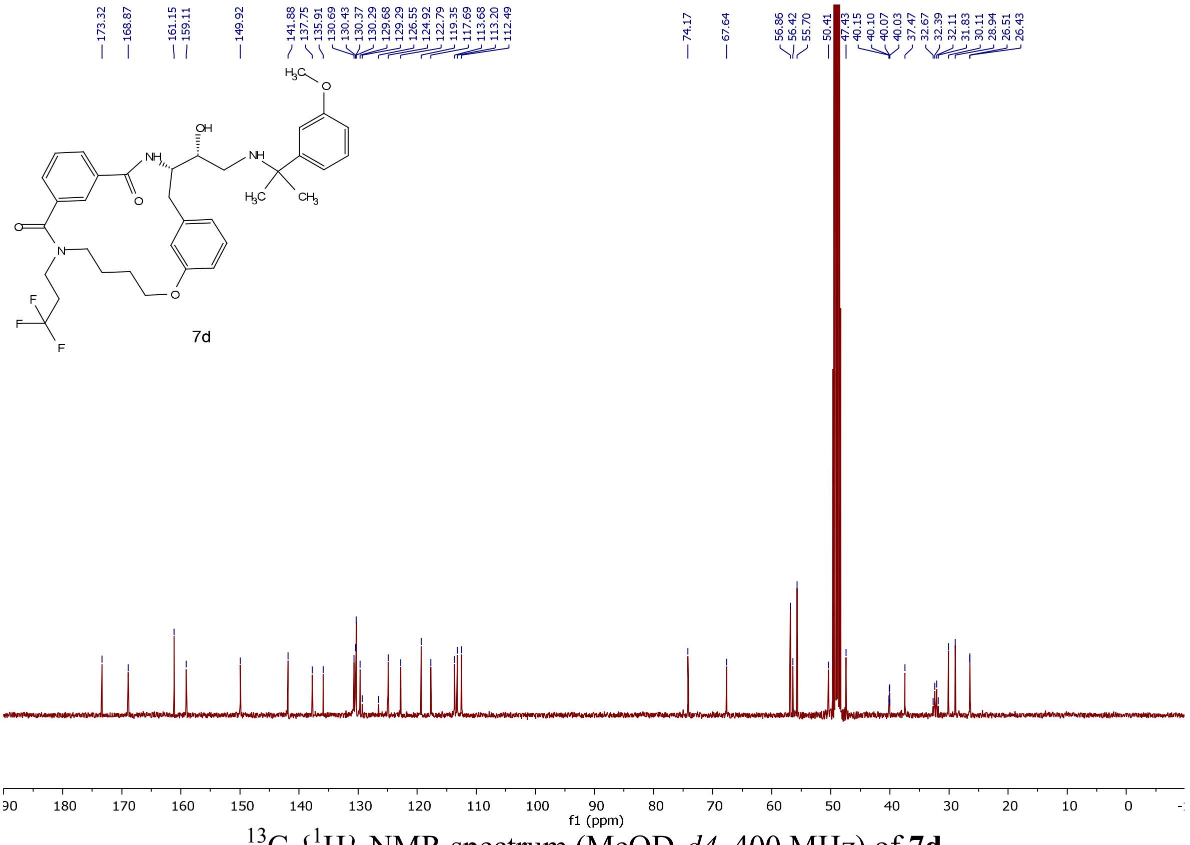

^{13}C {^{1}H} NMR spectrum (MeOD-*d4*, 400 MHz) of **7d**

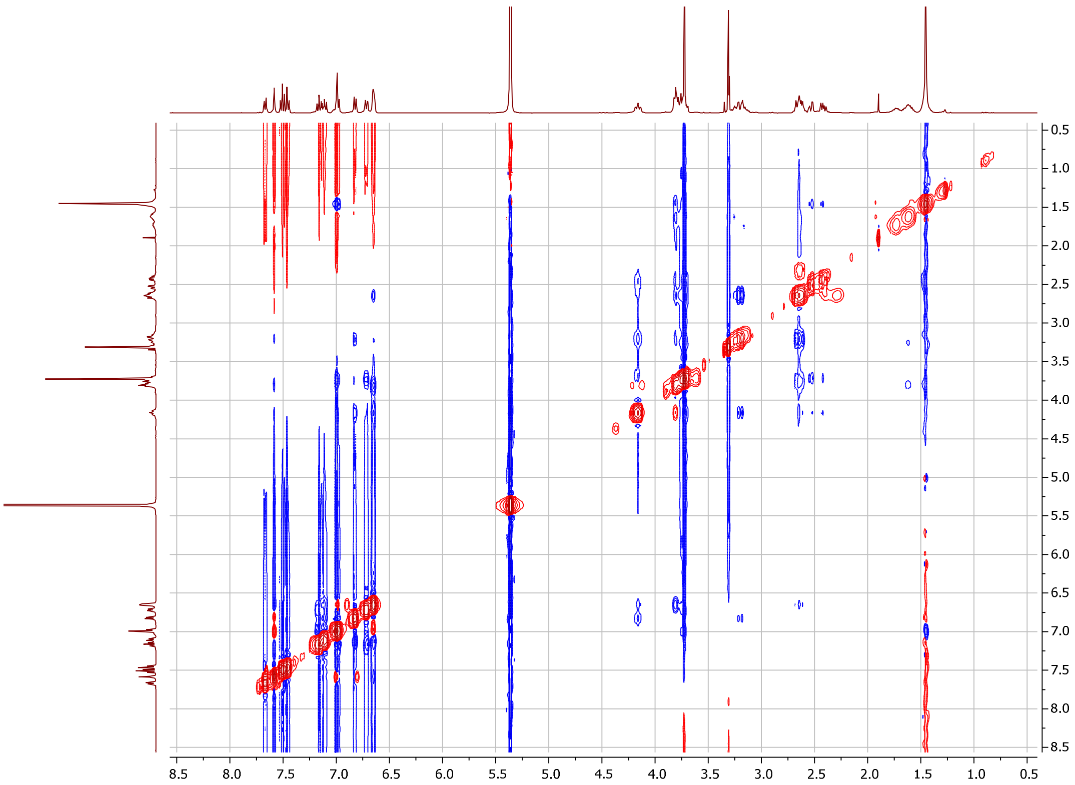

2D NOESY spectrum of compound **7d**

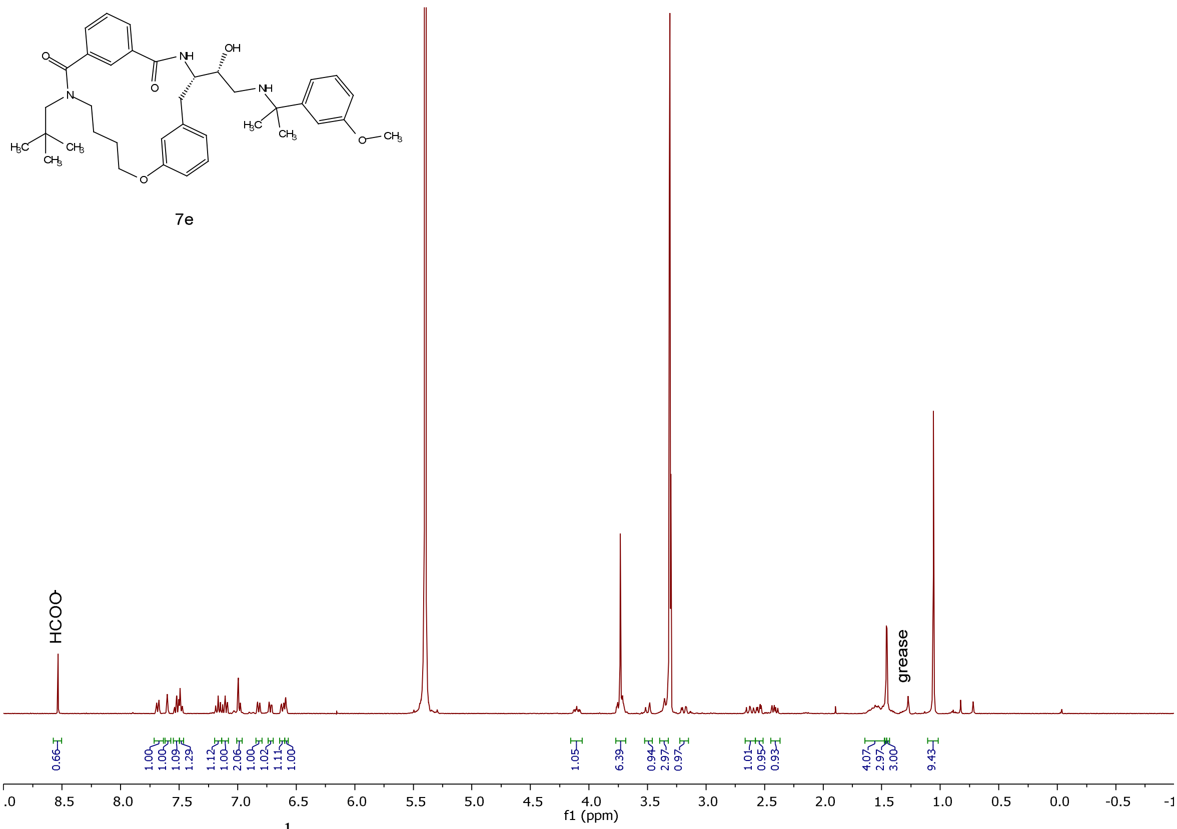

^{1}H NMR spectrum (MeOD-*d4*, 400 MHz) of **7e**

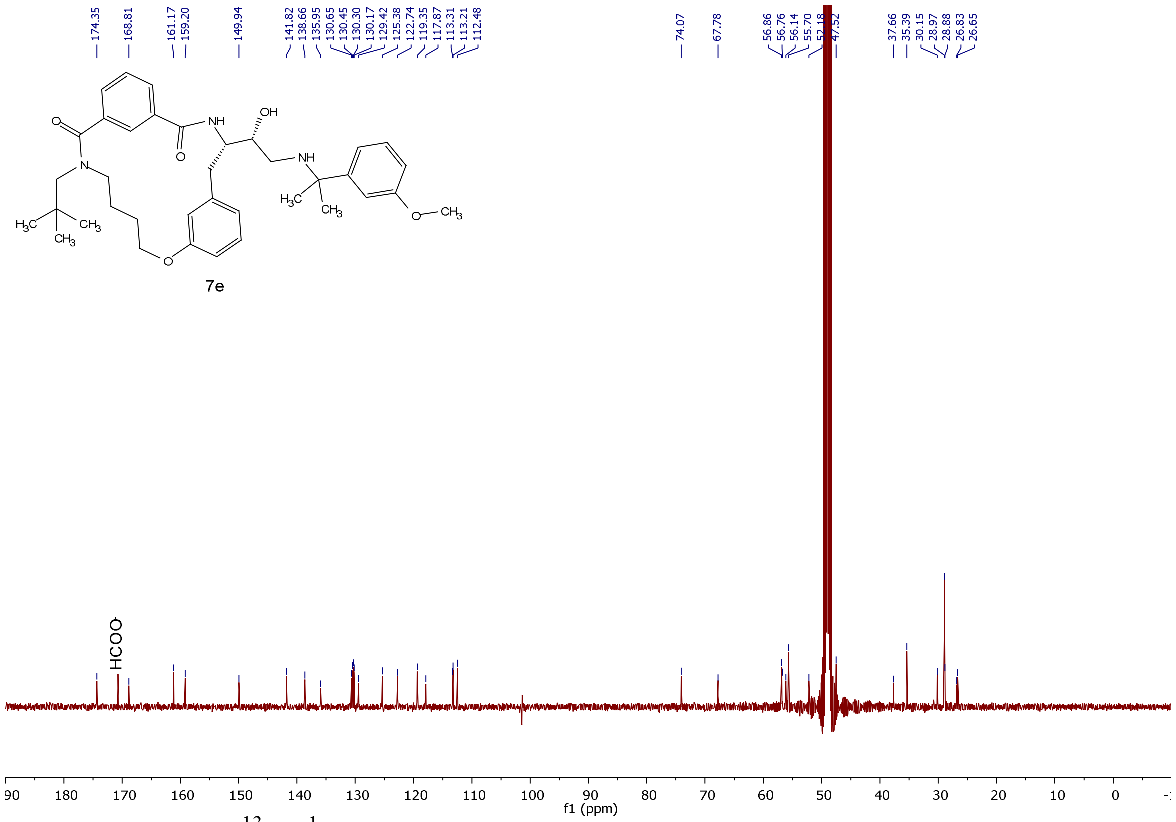

^{13}C {^{1}H} NMR spectrum (MeOD-*d4*, 400 MHz) of **7e**

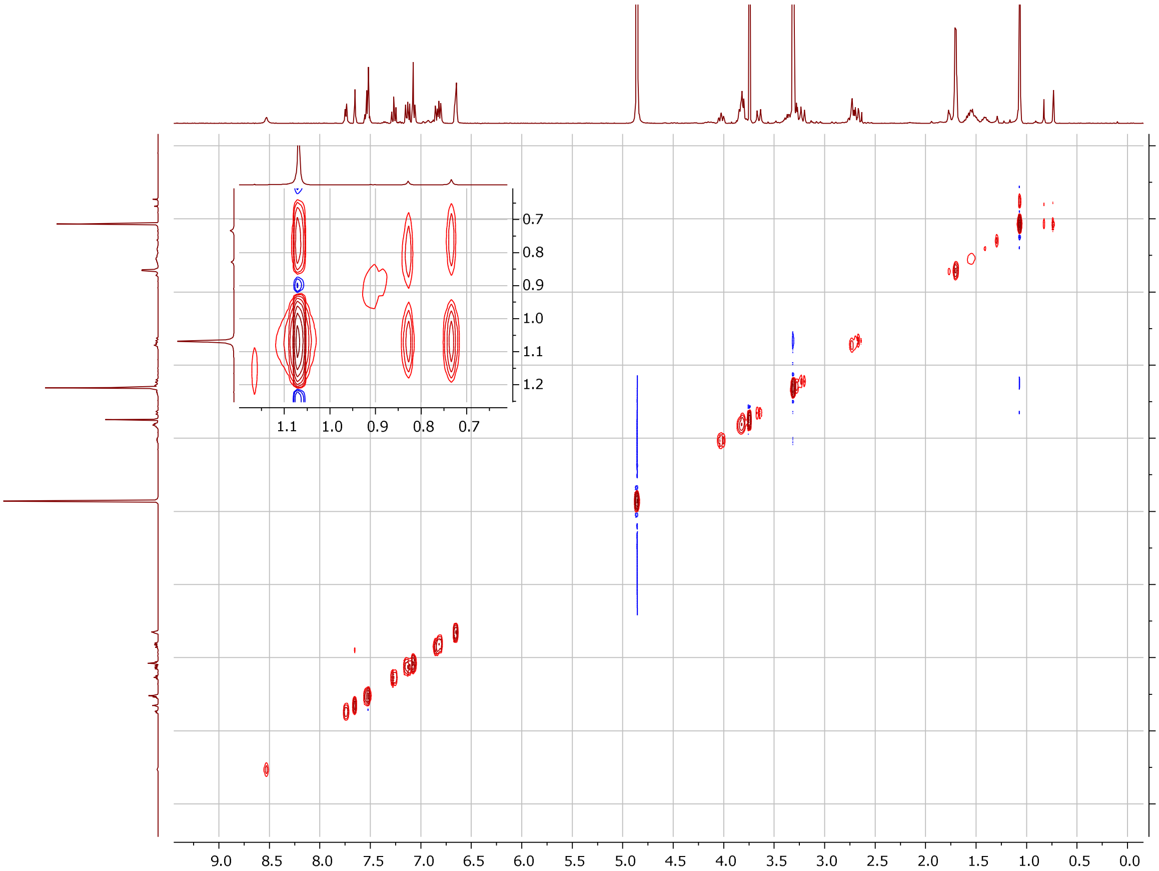

2D NOESY spectrum of compound **7e** (3 rotamers)

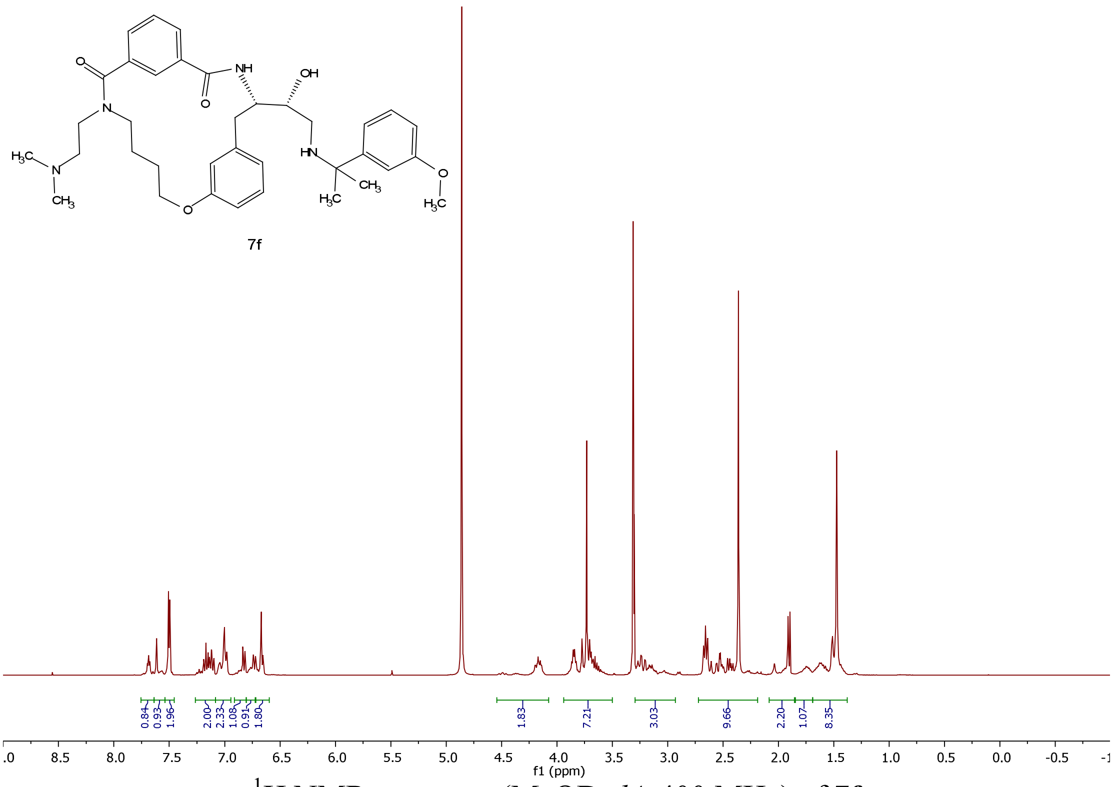

^{1}H NMR spectrum (MeOD-*d4*, 400 MHz) of **7f**

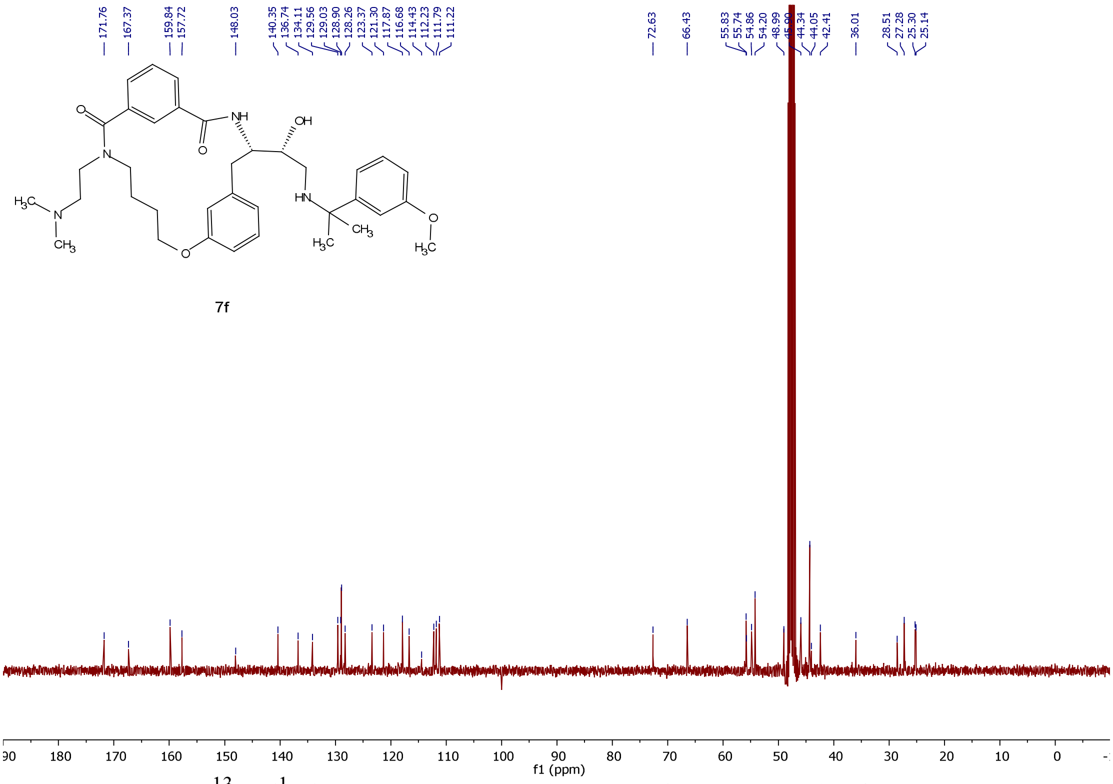

^{13}C {^{1}H} NMR spectrum (MeOD-*d4*, 400 MHz) of **7f**

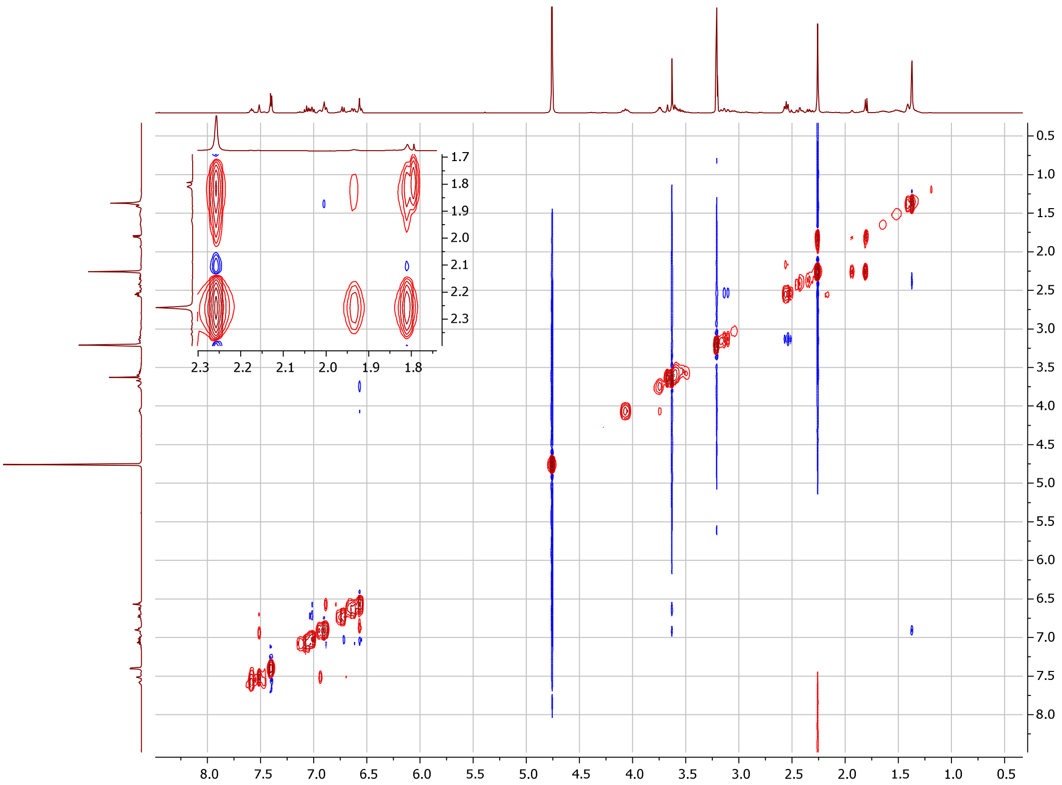

2D NOESY spectrum of compound **7f** (3 rotamers)

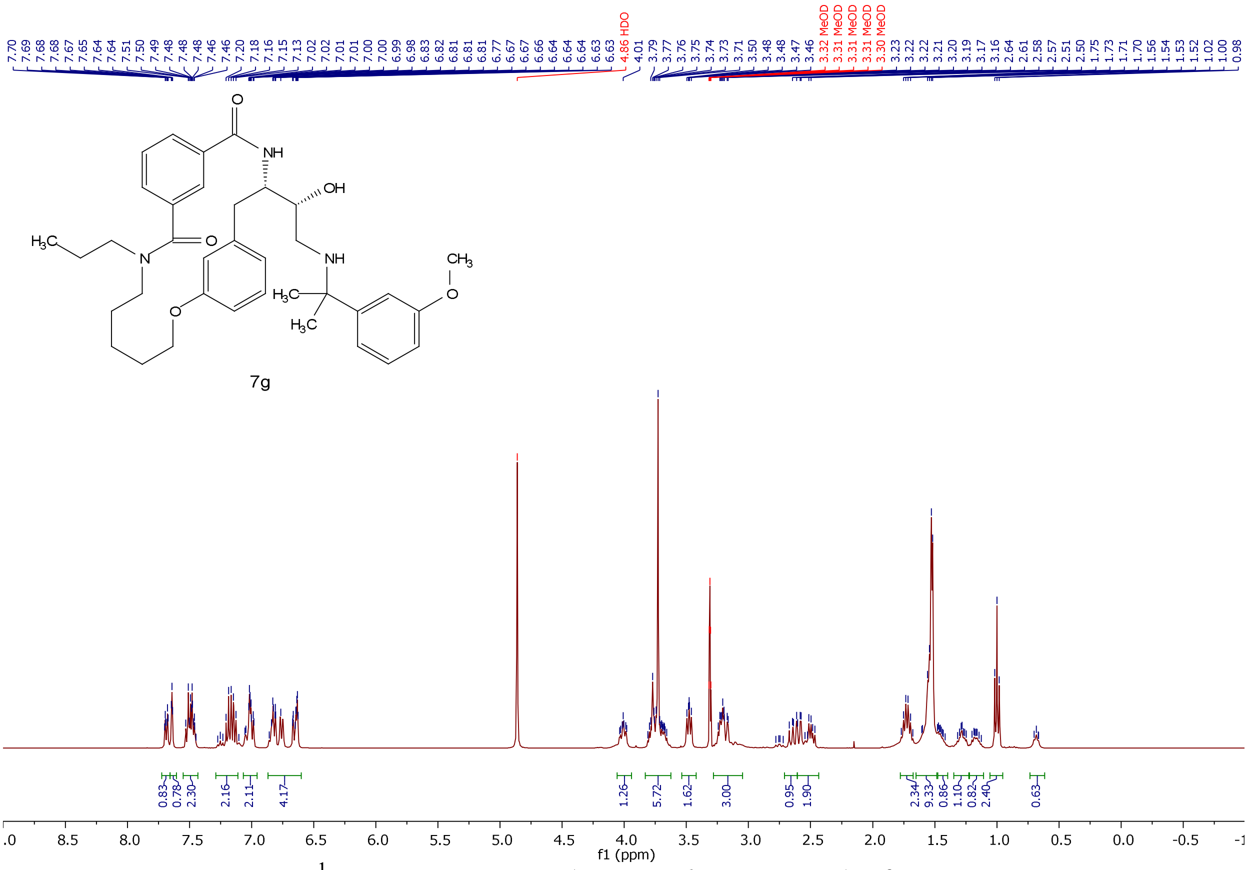

^{1}H NMR spectrum (MeOD-*d4*, 400 MHz) of **7g**

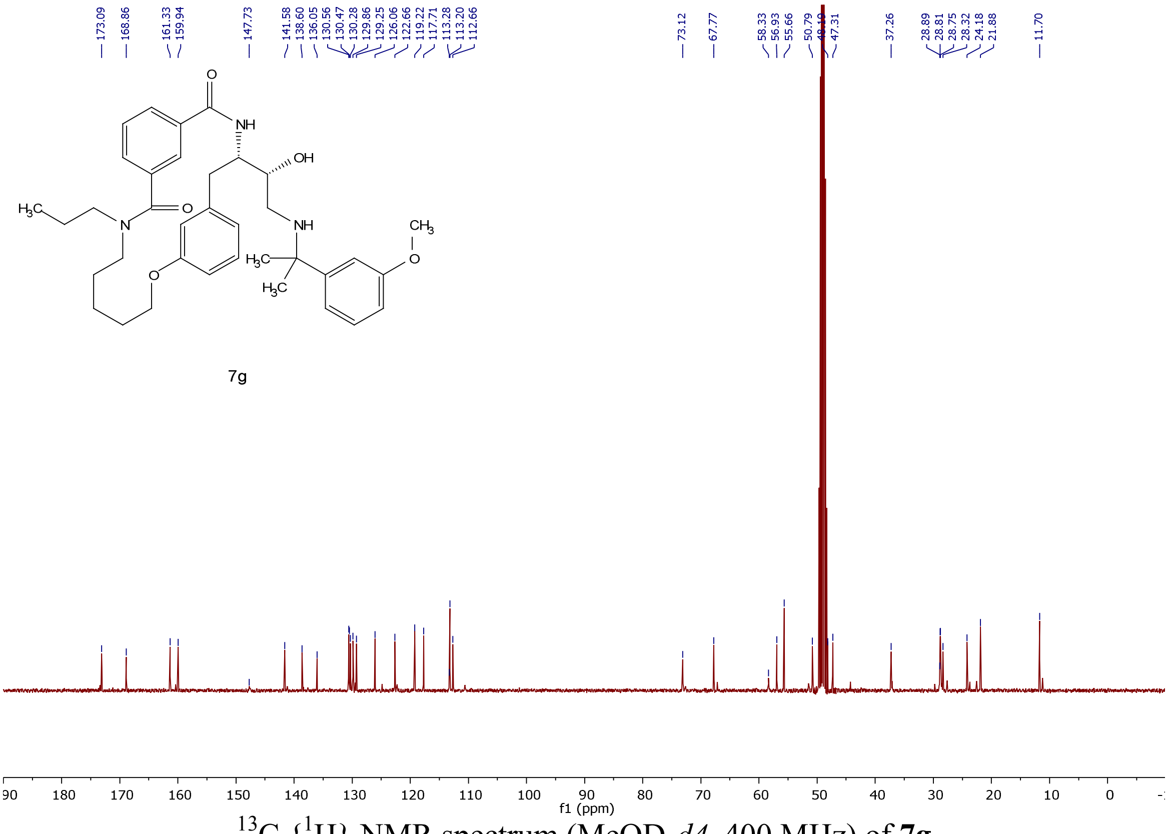

^{13}C {^{1}H} NMR spectrum (MeOD-*d4*, 400 MHz) of **7g**

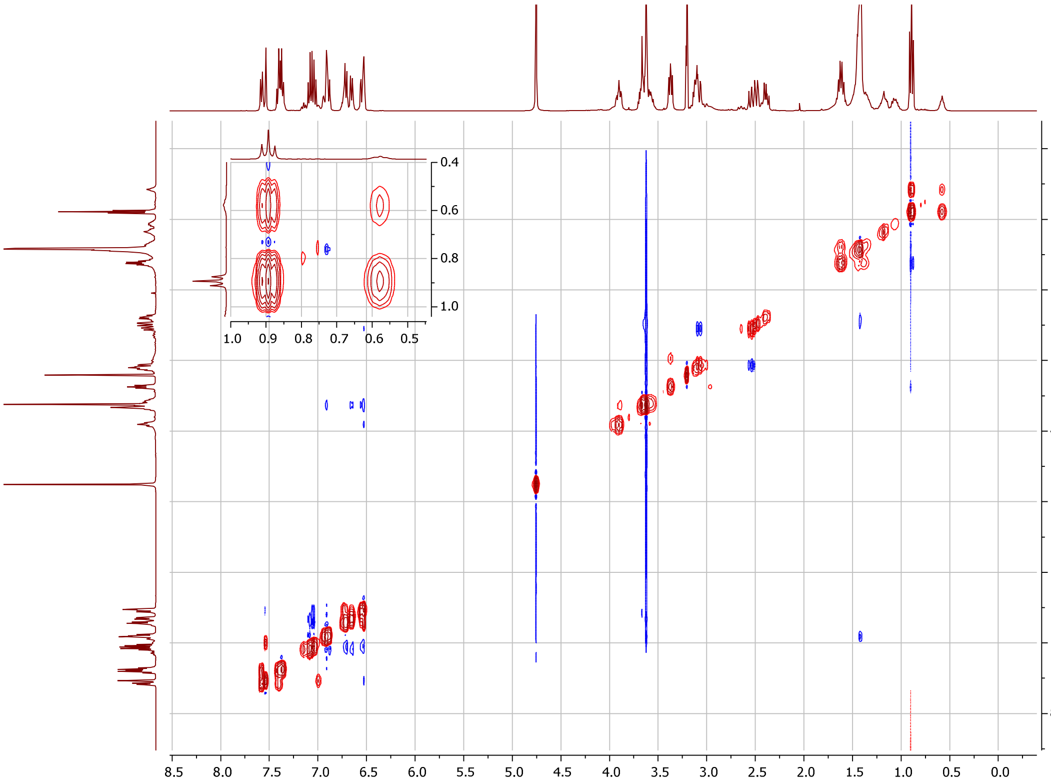

2D NOESY spectrum of compound **7g** (2 rotamers)

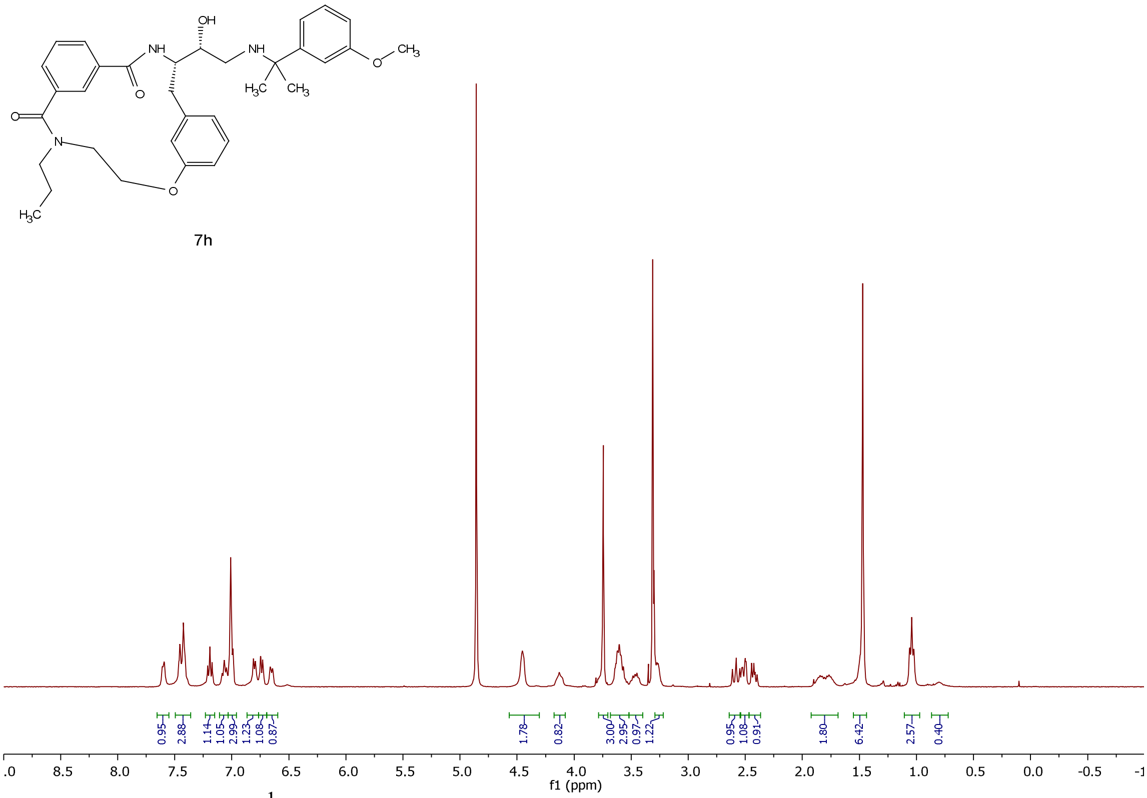

^{1}H NMR spectrum (MeOD-*d4*, 400 MHz) of **7h**

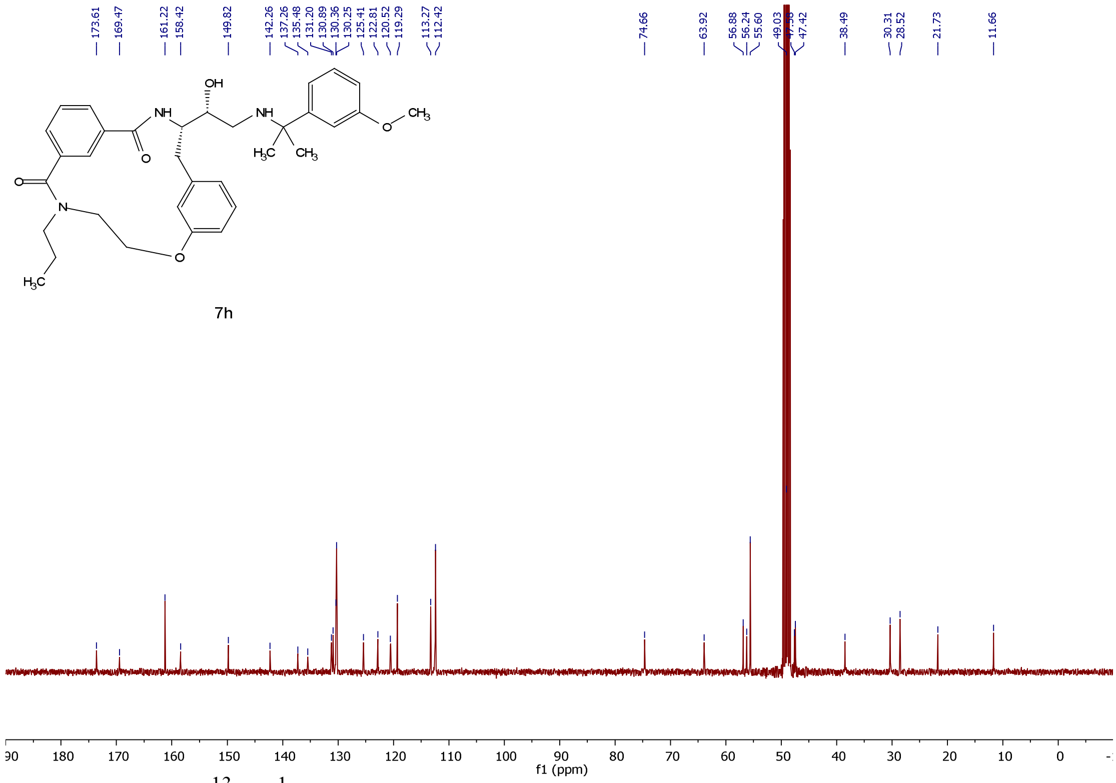

^{13}C {^{1}H} NMR spectrum (MeOD-*d4*, 400 MHz) of **7h**

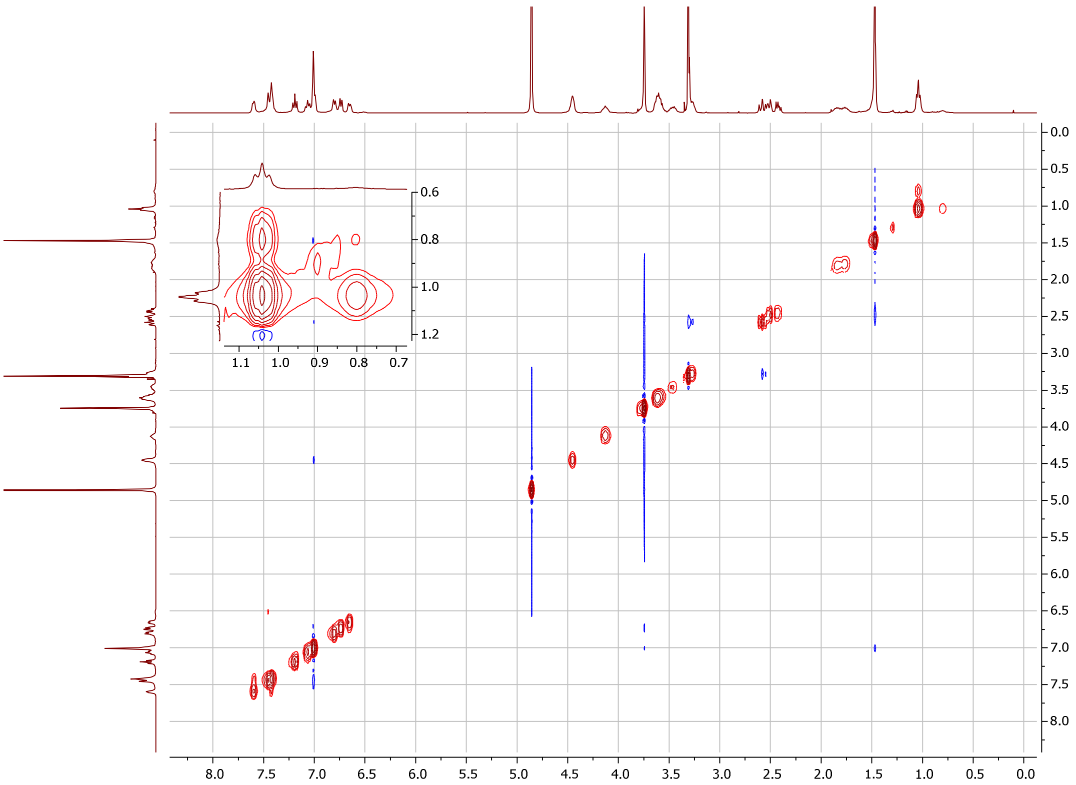

2D NOESY spectrum of compound **7h** (2 rotamers)

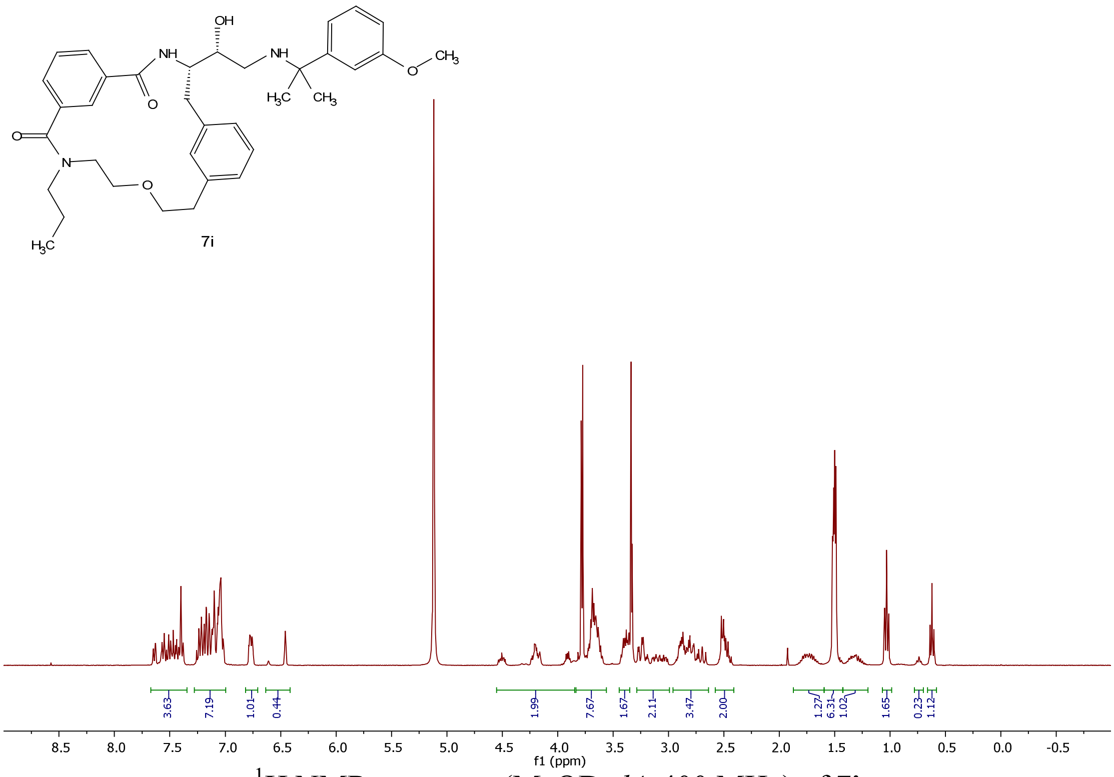

^{1}H NMR spectrum (MeOD-*d4*, 400 MHz) of **7i**

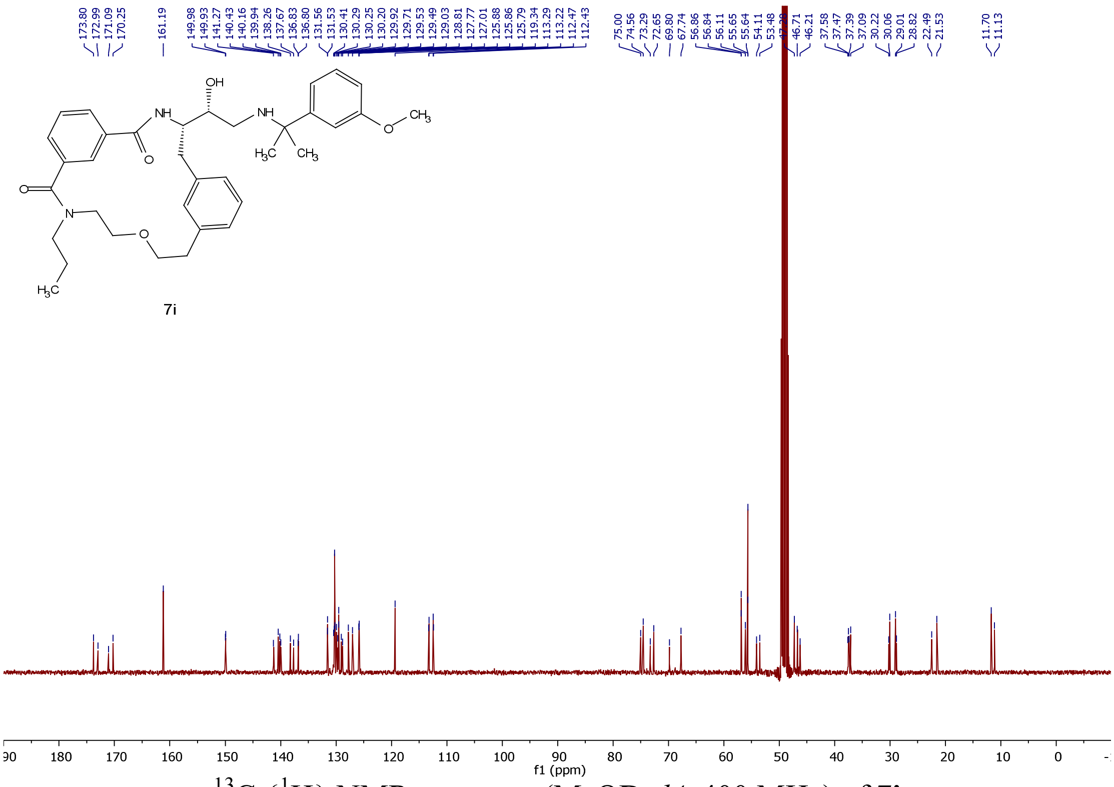

^{13}C {^{1}H} NMR spectrum (MeOD-*d4*, 400 MHz) of **7i**

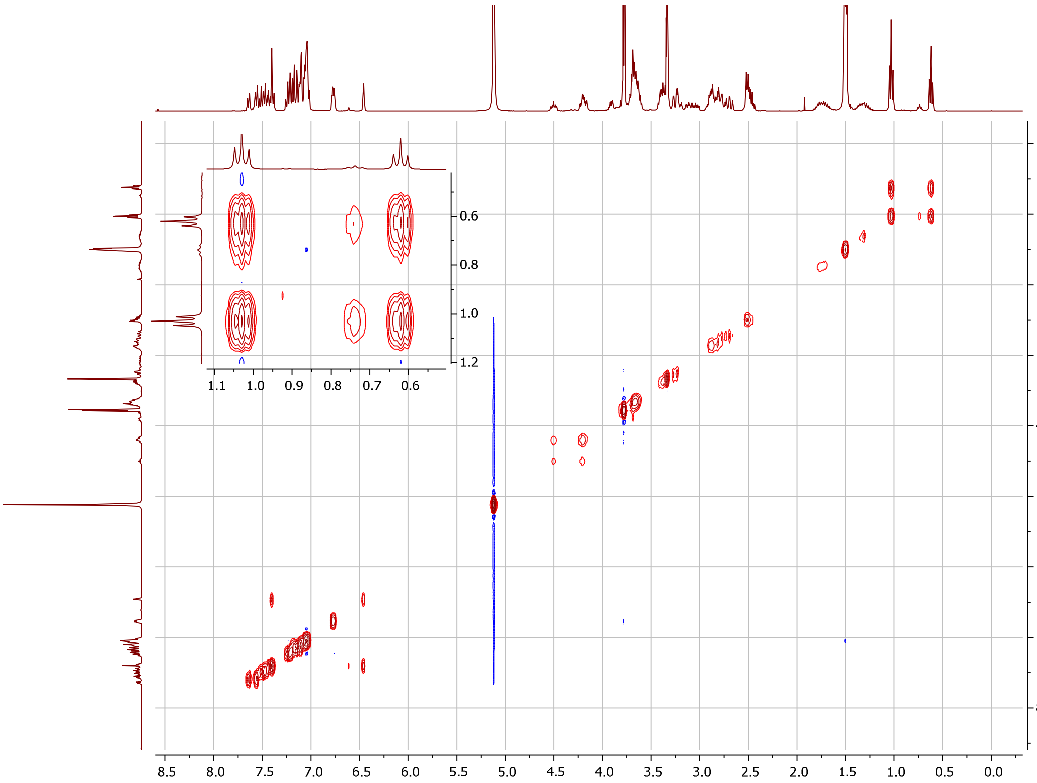

2D NOESY spectrum of compound **7i** (2 rotamers)

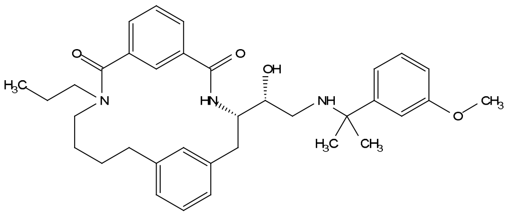

7j

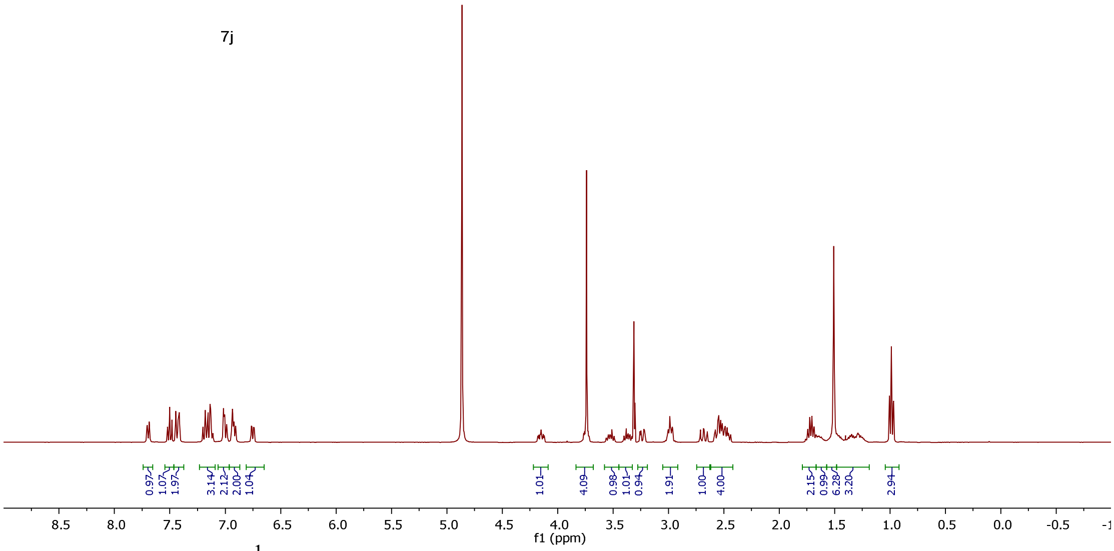

^{1}H NMR spectrum (MeOD-*d4*, 400 MHz) of **7j**

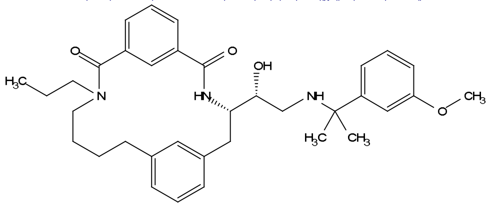

7j

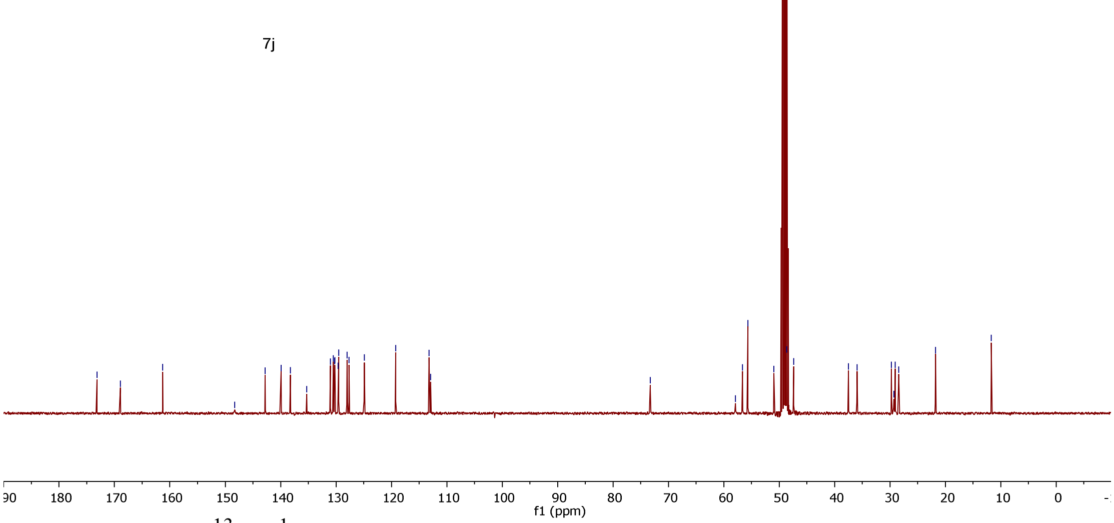

^{13}C {^{1}H} NMR spectrum (MeOD-*d4*, 400 MHz) of **7j**

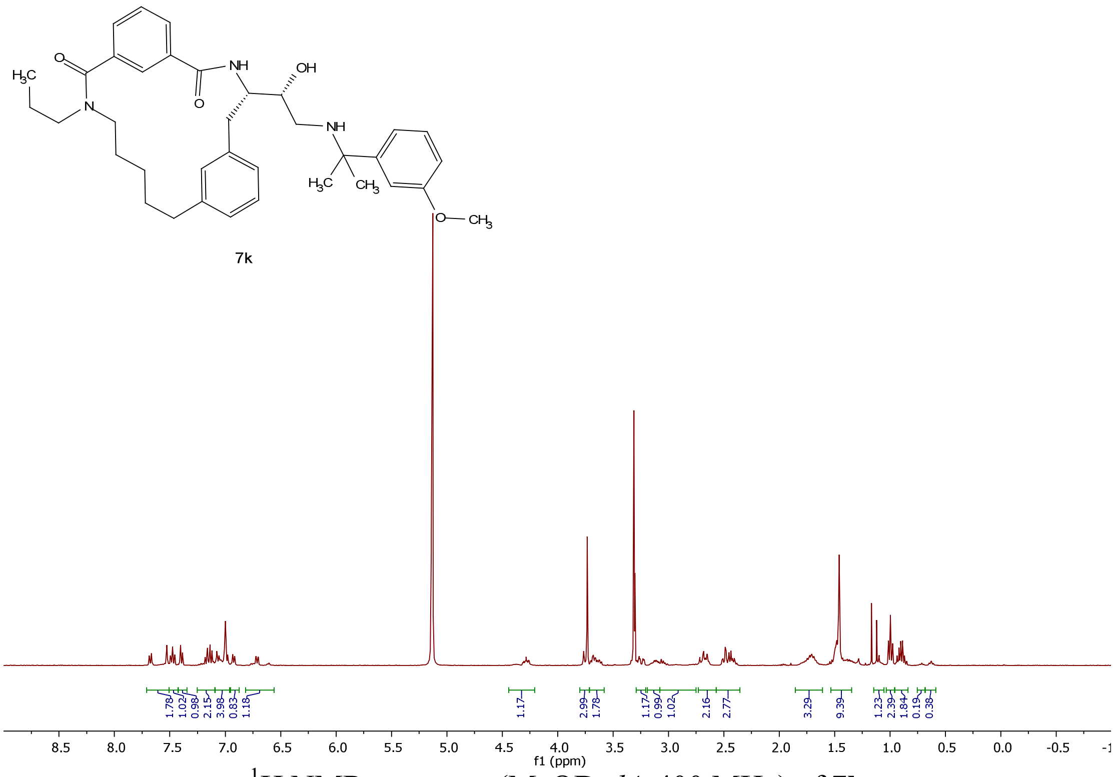

^{1}H NMR spectrum (MeOD-*d4*, 400 MHz) of **7k**

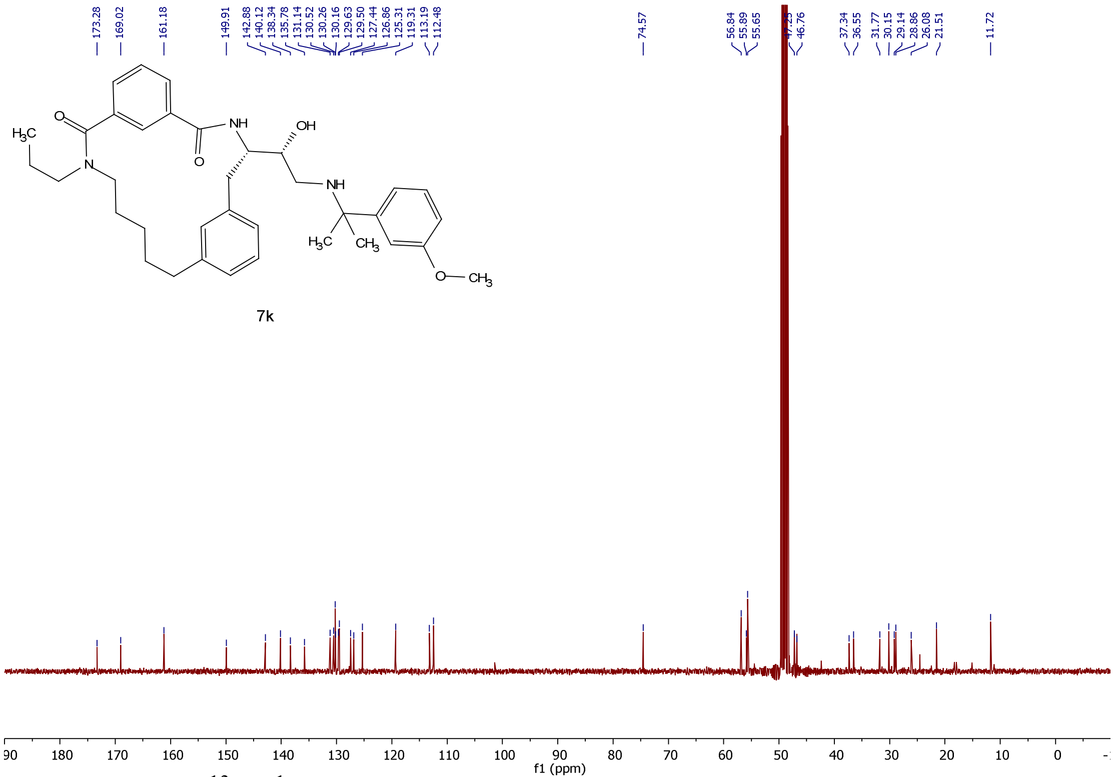

^{13}C {^{1}H} NMR spectrum (MeOD-*d4*, 400 MHz) of **7k**

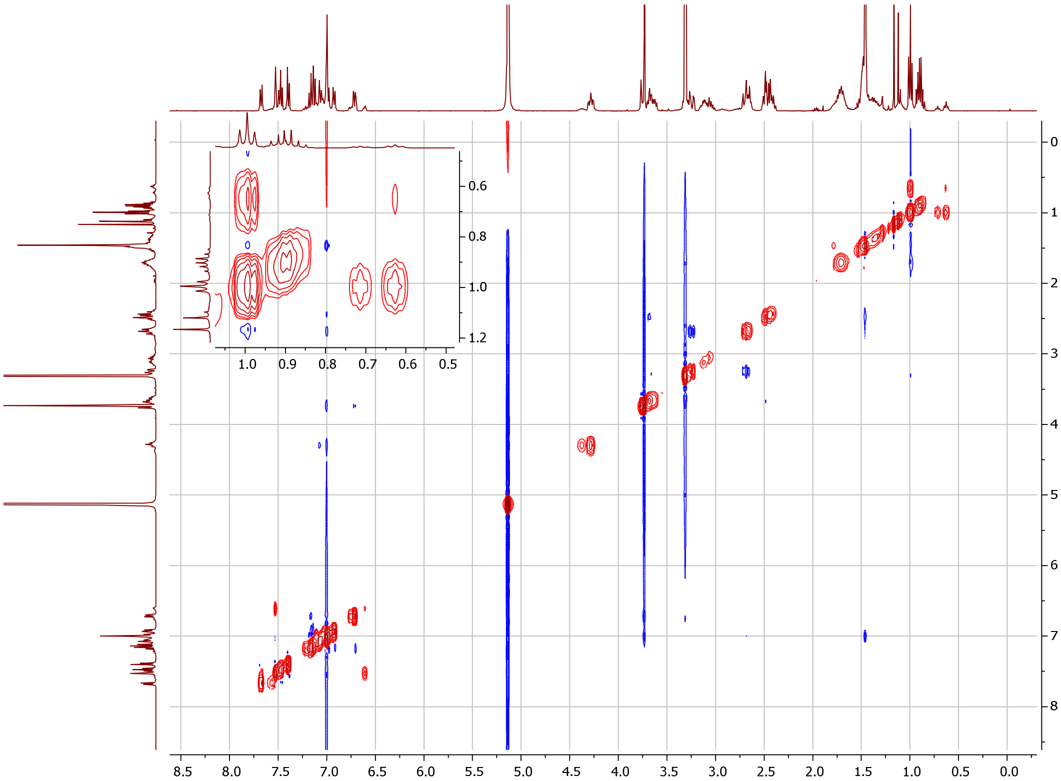

2D NOESY spectrum of compound **7k** (3 rotamers)

HPLC analysis of final products 7a-k

SAMPLE INFORMATION

Sample Name:	112-82-VAD-111	Acquired By:	System
Sample Type:	Unknown	Sample Set Name:	130223_serviss
Vial:	16	Acq. Method Set:	Serviss_Gr5%
Injection #:	1	Processing Method	Gr_5 %
Injection Volume:	10.00 ul	Channel Name:	2998 Ch1 254nm@4.8nm
Run Time:	25.0 Minutes	Proc. Chnl. Descr.:	2998 Ch1 254nm@4.8nm
Date Acquired:	13.02.2023 1:27:38 PM EET		
Date Processed:	13.02.2023 2:37:10 PM EET		

Apollo C18-13 5um (4.6x150 mm)
15 min Gr. 5-95%ACN +0,1%H3PO4; 5 min Iz. 95%ACN; 2 min Gr. 95-5% ACN; 3 min Iz. 5% ACN.
F=1mL/min. T=40oC.

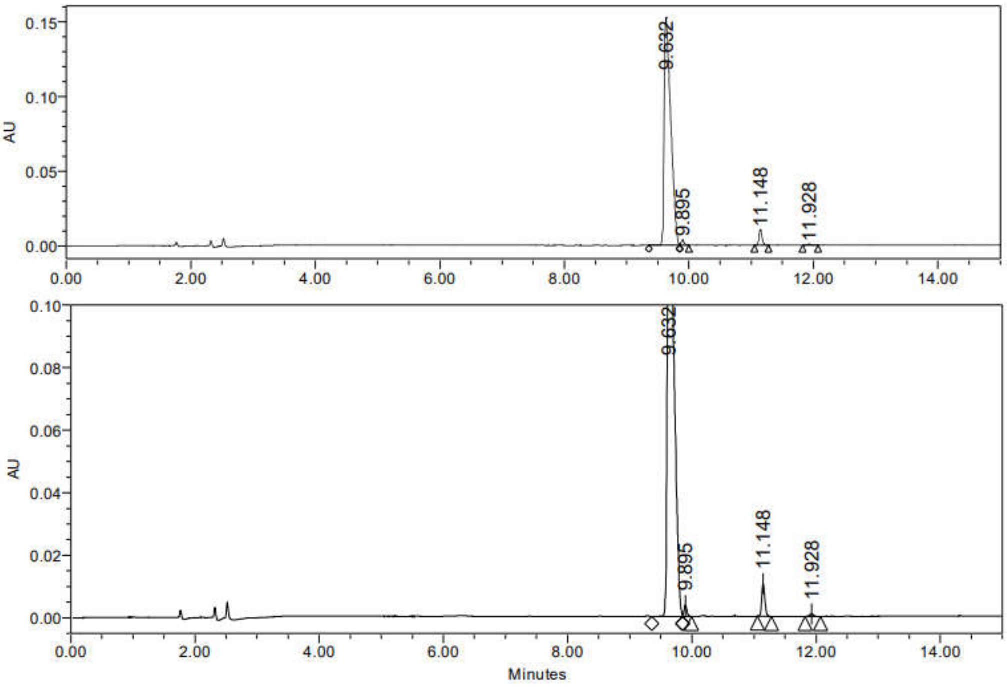

C=0.35mg/ml diluent (10%ACN+0,1%H3PO4)

	RT	Area	% Area	Height	EP Plate Count	Resolution	Selectivity	Width @ 50%
1	9.632	1100731	95.60	152252	38197			0.116
2	9.895	11467	1.00	3529	216655	1.87	1.03	0.050
3	11.148	36030	3.13	10409	240977	14.28	1.15	0.053
4	11.928	3186	0.28	748	167288	7.54	1.08	0.069
Sum		1151414.9						

HPLC of **7a**

SAMPLE INFORMATION

Sample Name:	110-80-VAd-581	Acquired By:	System
Sample Type:	Unknown	Sample Set Name:	130223_serviss
Vial:	14	Acq. Method Set:	Serviss_Gr5%
Injection #:	1	Processing Method	Gr_5 %
Injection Volume:	10.00 ul	Channel Name:	2998 Ch1 254nm@4.8nm
Run Time:	25.0 Minutes	Proc. Chnl. Descr.:	2998 Ch1 254nm@4.8nm
Date Acquired:	13.02.2023 12:10:05 PM EET		
Date Processed:	13.02.2023 12:59:27 PM EET		

Apollo C18-13 5um (4.6x150 mm)
15 min Gr. 5-95%ACN +0,1%H3PO4; 5 min Iz. 95%ACN; 2 min Gr. 95-5% ACN; 3 min Iz. 5% ACN.
F=1mL/min. T=40oC.

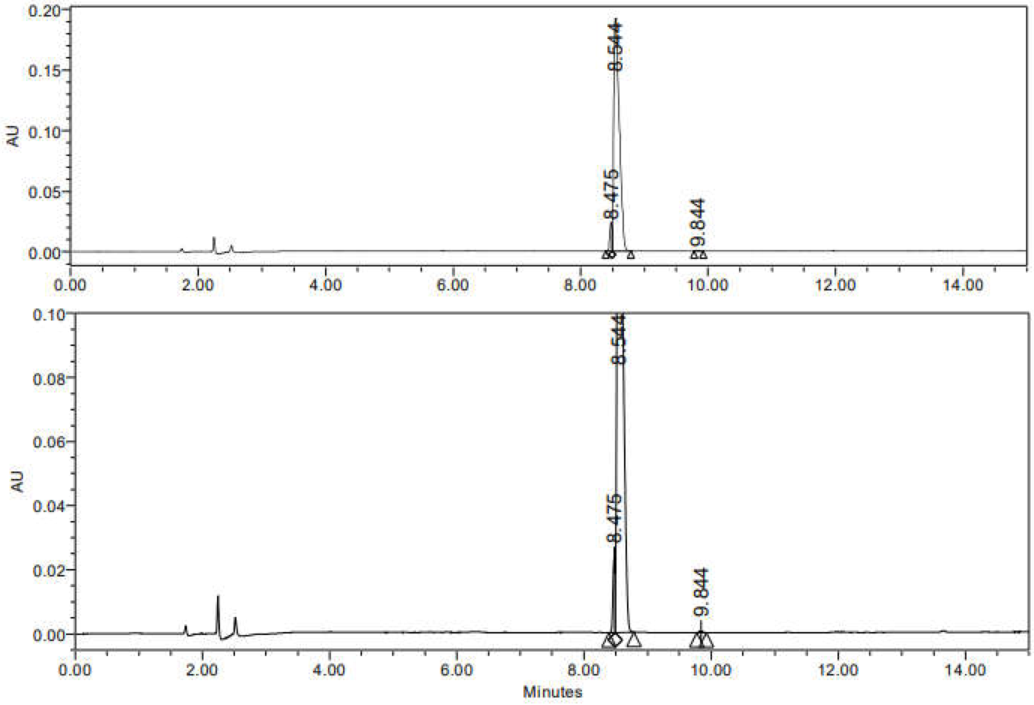

C=0.35mg/ml diluent (50%ACN+0,1%H3PO4)

	RT	Area	% Area	Height	EP Plate Count	Resolution	Selectivity	Width @ 50%
1	8.475	54785	4.50	23332				
2	8.544	1160151	95.37	192122	42757		1.01	0.097
3	9.844	1562	0.13	440	169697	10.00	1.19	0.056
Sum		1216498.5						

HPLC of **7b**

SAMPLE INFORMATION

Sample Name:	167-88-VAD-577	Acquired By:	System
Sample Type:	Unknown	Sample Set Name:	010323_serviss
Vial:	18	Acq. Method Set:	Serviss_Gr5%
Injection #:	1	Processing Method	Gr_5 %
Injection Volume:	5.00 ul	Channel Name:	2998 Ch1 254nm@4.8nm
Run Time:	25.0 Minutes	Proc. Chnl. Descr.:	2998 Ch1 254nm@4.8nm
Date Acquired:	01.03.2023 10:57:13 AM EET		
Date Processed:	01.03.2023 11:15:47 AM EET		

Apollo C18-13 5um (4.6x150 mm)
15min Gr 5-95%ACN + 0.1%H3PO4; 5min IZ 95%ACN; 2min Gr 95-5% ACN; 3min IZ 5%ACN.
F=1mL/min. T=40oC.

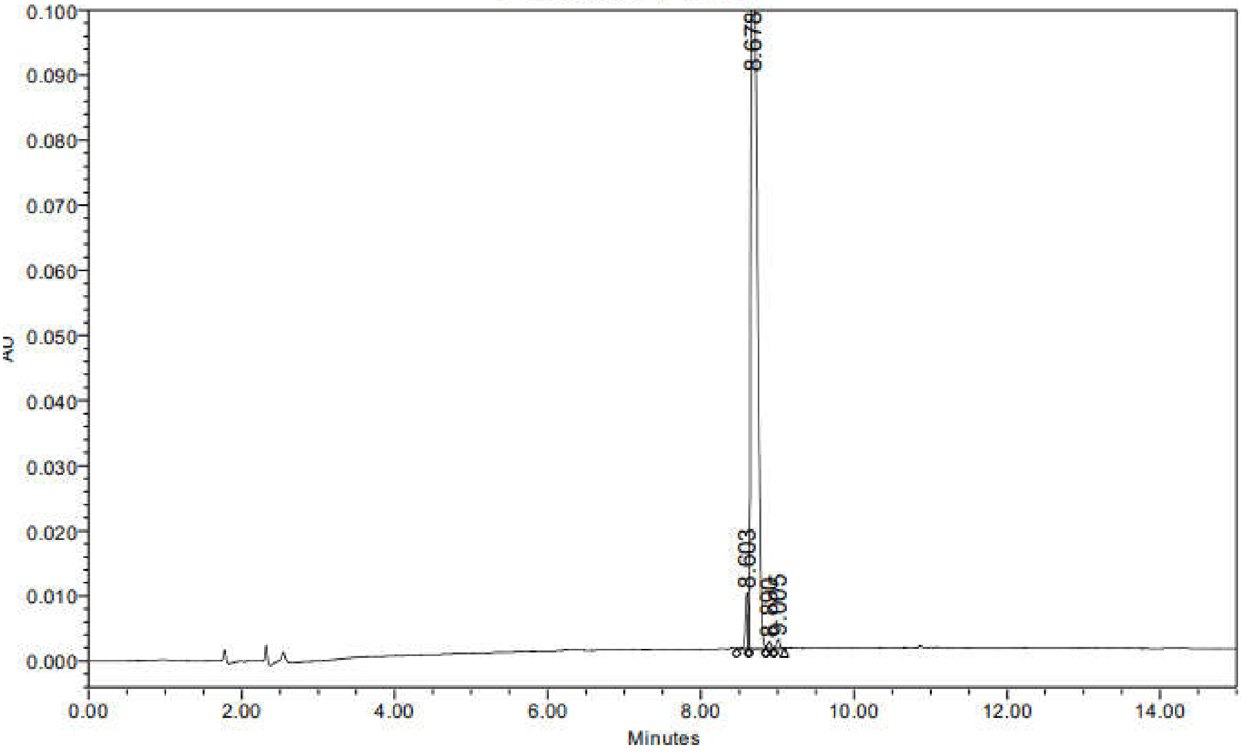

C=0,5mg/ml (25%ACN+0,1%H3PO4)

	RT	Area	% Area	Height	EP Plate Count	Resolution	Selectivity	Width @ 50%
1	8.603	22633	3.13	8592				
2	8.678	693263	95.81	134648	61452		1.01	0.082
3	8.890	3468	0.48	1006	147570	1.83	1.03	0.054
4	9.005	4212	0.58	1346	199927	1.33	1.02	0.047
Sum		723576.8						

HPLC of **7c**

SAMPLE INFORMATION			
Sample Name:	117-87-MBO-120	Acquired By:	System
Sample Type:	Unknown	Sample Set Name:	140223_serviss
Vial:	25	Acq. Method Set:	Serviss_Gr40%
Injection #:	1	Processing Method	Gr_40%
Injection Volume:	10.00 ul	Channel Name:	2998 Ch1 254nm@4.8nm
Run Time:	25.0 Minutes	Proc. Chnl. Descr.:	2998 Ch1 254nm@4.8nm
Date Acquired:	14.02.2023 9:48:30 AM EET		
Date Processed:	14.02.2023 11:23:19 AM EET		

Apollo C18-13 5um (4.6x150 mm)
15 min Gr. 40-95%ACN + 0.1%H3PO4; 5 min Iz. 95%ACN; 2 min Gr. 95-40% ACN; 3 min Iz. 40% ACN.
F=1mL/min. T=40oC.

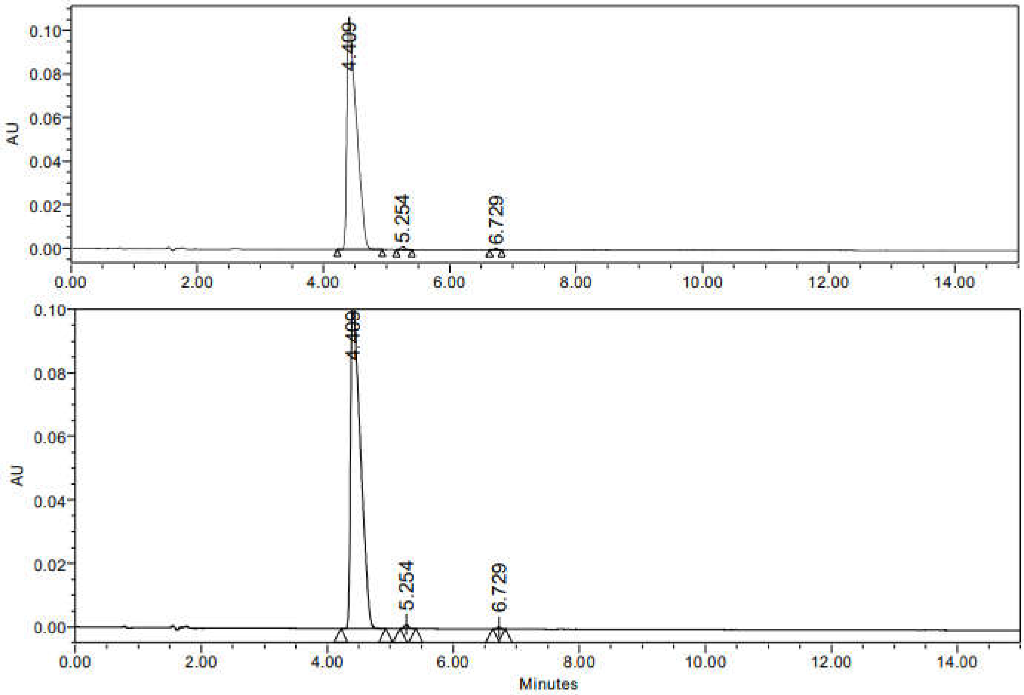

C=0,35mg/ml (50%ACN+0,1%H3PO4)

	RT	Area	% Area	Height	EP Plate Count	Resolution	Selectivity	Width @ 50%
1	4.409	1095146	99.27	106347	3962			0.165
2	5.254	5651	0.51	1260	31668	4.26	1.30	0.069
3	6.729	2449	0.22	591	59837	12.96	1.40	0.065
Sum		1103246.2						

HPLC of **7d**

SAMPLE INFORMATION			
Sample Name:	108-78-VAD-193	Acquired By:	System
Sample Type:	Unknown	Sample Set Name:	130223_serviss
Vial:	20	Acq. Method Set:	Serviss_Gr40%
Injection #:	1	Processing Method	Gr_40%
Injection Volume:	10.00 ul	Channel Name:	2998 Ch1 254nm@4.8nm
Run Time:	25.0 Minutes	Proc. Chnl. Descr.:	2998 Ch1 254nm@4.8nm
Date Acquired:	13.02.2023 5:00:26 PM EET		
Date Processed:	14.02.2023 10:25:32 AM EET		

Apollo C18-13 5um (4.6x150 mm)
15min Gr 40-95%ACN + 0.1%H3PO4; 5min IZ 95%ACN; 2min Gr 95-40% ACN; 3min IZ 40%ACN.
F=1mL/min. T=40oC.

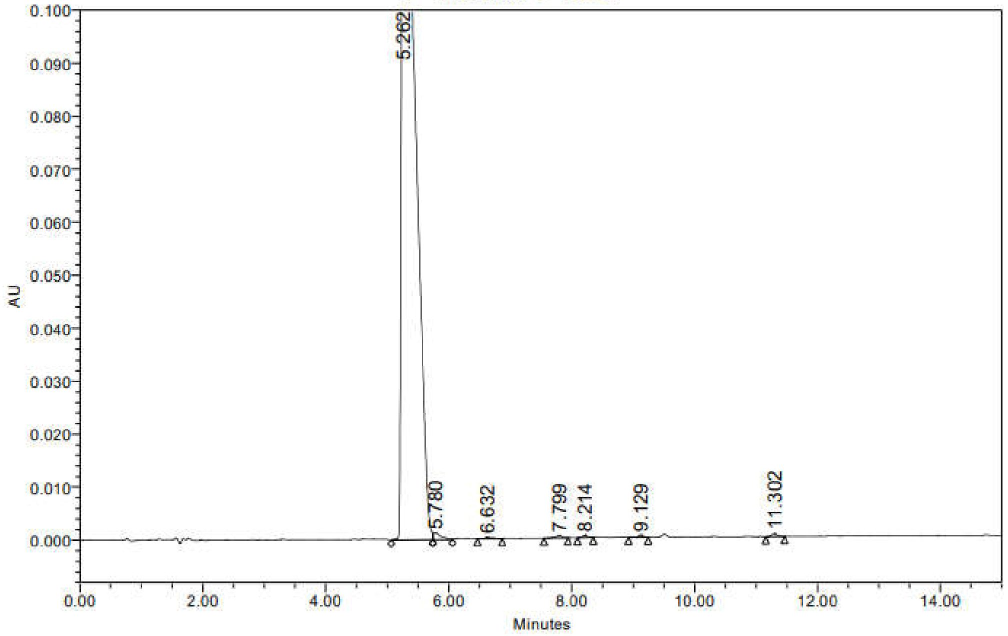

C=0,5mg/m (40%ACN+0,1%H3PO4)

	RT	Area	% Area	Height	EP Plate Count	Resolution	Selectivity	Width @ 50%
1	5.262	2319643	98.90	161191	2833			0.233
2	5.780	11962	0.51	1298			1.14	
3	6.632	2513	0.11	311	10069		1.20	0.156
4	7.799	3649	0.16	527	43654	5.65	1.23	0.088
5	8.214	2371	0.10	462	57209	2.90	1.07	0.081
6	9.129	2239	0.10	419	74898	6.78	1.14	0.079
7	11.302	3174	0.14	585	106236	16.02	1.29	0.082
Sum		2345549.9						

HPLC of **7e**

SAMPLE INFORMATION

Sample Name:	109-79-VAD-614		
Sample Type:	Unknown	Sample Set Name:	09022023_Serviss
Vial:	5	Acq. Method Set:	Serviss Gr5%_iz_UV
Injection #:	1	Processing Method:	Gr 5%
Injection Volume:	10.00 ul	Channel Name:	2998 Ch1 254nm@1.2nm
Run Time:	25.0 Minutes	Proc. Chnl. Descr.:	2998 Ch1 254nm@1.2nm
Date Acquired:	2/13/2023 12:42:45 PM EET	Acquired By:	System
Date Processed:	2/13/2023 1:56:46 PM EET		

Adamas C18-2 (4,6x150 mm)
15 min Gr. 5 - 95% ACN + 0.1% H3PO4; 5 min Iz. 95% ACN; 3 min Gr. 95-5% ACN; 2 min Iz. 5% ACN; F = 1mL/min; 40oC

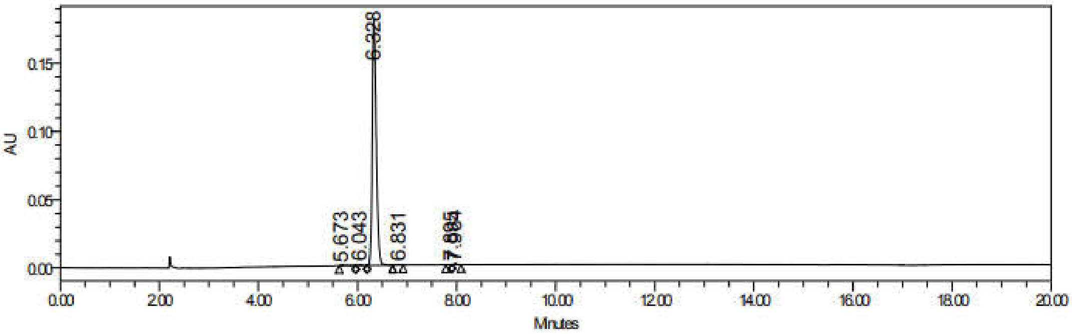

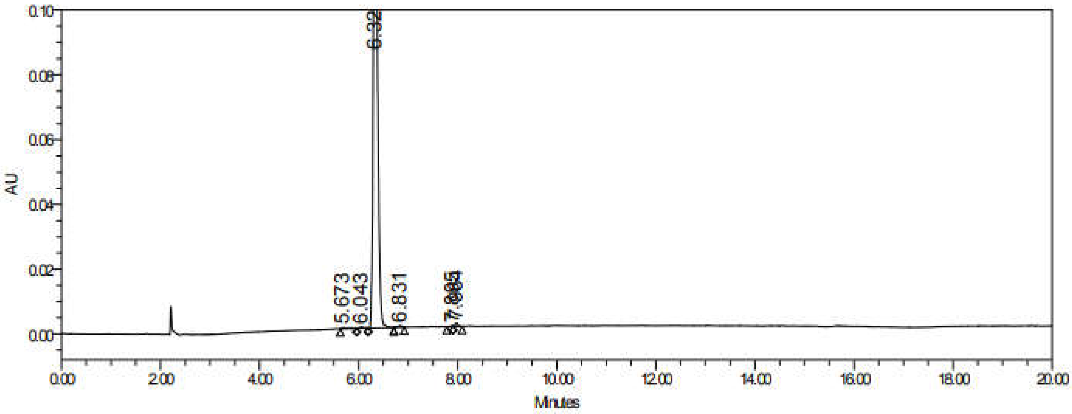

C=0.3 mg/ml (50% ACN/0.1% H3PO4)

	RT	Area	%Area	Height	EP Plate Count	Width@50%	Resolution	Selectivity
1	5.673	4172	0.40	336	27612	0.0804		
2	6.043	3181	0.30	389	5132	0.1986	1.567	1.088
3	6.328	1031952	98.53	180789	29801	0.0863	1.178	1.062
4	6.831	2696	0.26	460	32064	0.0898	3.372	1.103
5	7.895	833	0.08	245				1.198
6	7.964	4542	0.43	936	70035	0.0708		1.011
Sum		1047376.6						

HPLC of **7f**

SAMPLE INFORMATION

Sample Name:	115-85-VAD-180		
Sample Type:	Unknown	Sample Set Name:	14022023_Serviss
Vial:	12	Acq. Method Set:	Serviss Gr5%_iz_UV
Injection #:	1	Processing Method:	Gr 5%
Injection Volume:	10.00 ul	Channel Name:	2998 Ch1 254nm@1.2nm
Run Time:	25.0 Minutes	Proc. Chnl. Descr.:	2998 Ch1 254nm@1.2nm
Date Acquired:	2/14/2023 10:04:27 AM EET	Acquired By:	System
Date Processed:	2/14/2023 10:27:41 AM EET		

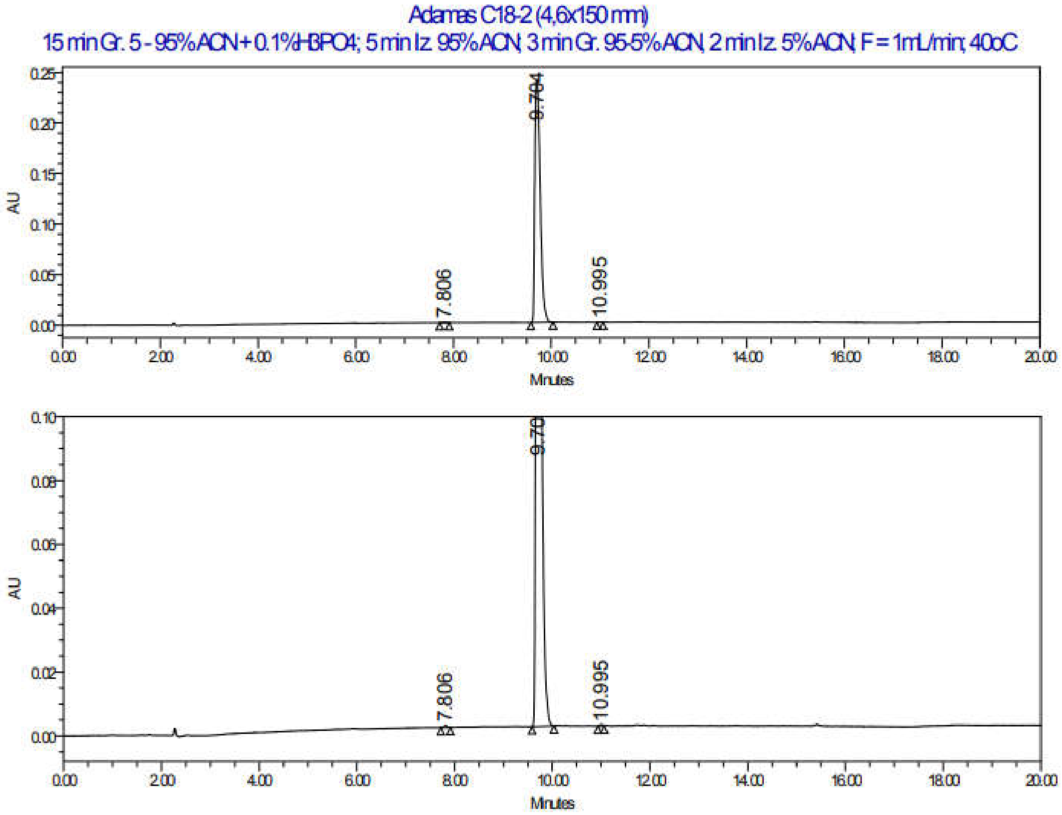

C=0.55 mg/ml (10% ACN/0.1% H3PO4)

	RT	Area	%Area	Height	EP Plate Count	Width @50%	Resolution	Selectivity
1	7.806	2199	0.12	510	90330	0.0611		
2	9.704	1877422	99.81	239946	33896	0.1241	12.094	1.299
3	10.995	1285	0.07	357	238831	0.0530	8.611	1.157
Sum		1880906.2						

HPLC of **7g**

SAMPLE INFORMATION			
Sample Name:	158-83-VAD-114	Acquired By:	System
Sample Type:	Unknown	Sample Set Name:	280223_serviss
Vial:	16	Acq. Method Set:	Serviss_Gr5%
Injection #:	1	Processing Method	Gr_5 %_254
Injection Volume:	10.00 ul	Channel Name:	2998 Ch1 254nm@4.8nm
Run Time:	25.0 Minutes	Proc. Chnl. Descr.:	2998 Ch1 254nm@4.8nm
Date Acquired:	28.02.2023 9:50:43 AM EET		
Date Processed:	28.02.2023 10:15:32 AM EET		

Apollo C18-13 5um (4.6x150 mm)
15 min Gr. 5-95%ACN +0,1%H3PO4; 5 min Iz. 95%ACN; 2 min Gr. 95-5% ACN; 3 min Iz. 5% ACN.
F=1mL/min. T=40oC.

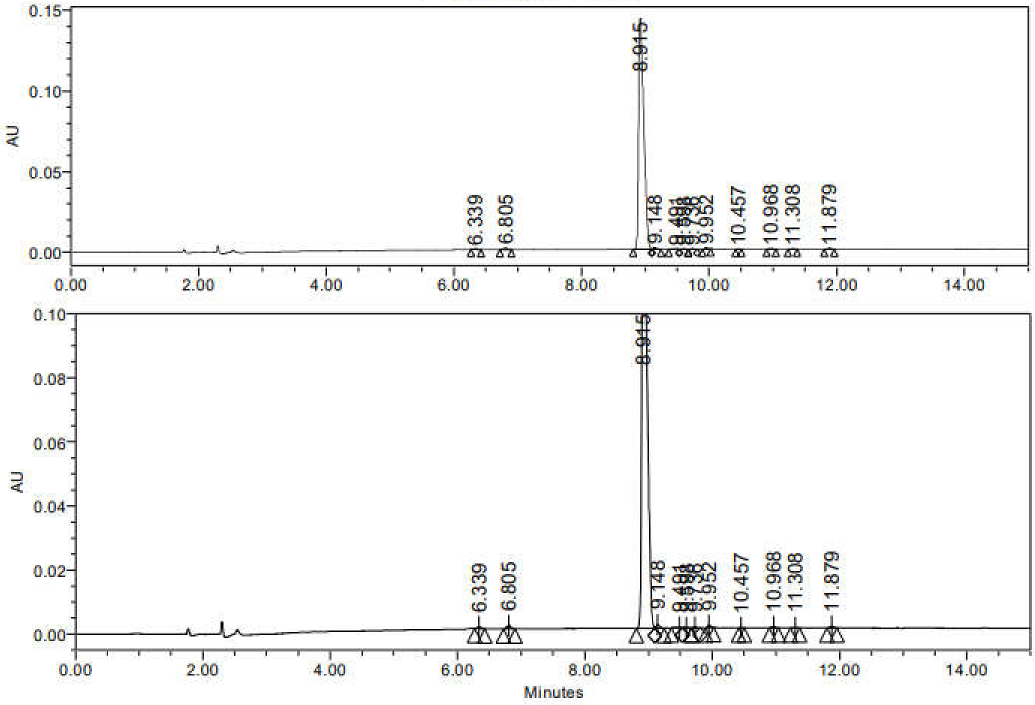

C=0,35mg/ml(20%ACN+0,1%H3PO4)

	RT	Area	% Area	Height	EP Plate Count	Resolution	Selectivity	Width @ 50%
1	6.339	2287	0.27	601	62627			0.060
2	6.805	4069	0.49	1001	63712	4.48	1.10	0.063
3	8.915	817596	97.57	143107	52645	16.07	1.40	0.091
4	9.148	4086	0.49	1074	132721	1.83	1.03	0.059
5	9.491	1501	0.18	258	37811	2.32	1.05	0.115
6	9.598	985	0.12	194	66541	0.62	1.01	0.088
7	9.736	952	0.11	306	250547	1.22	1.02	0.046
8	9.952	2102	0.25	723	267010	2.80	1.03	0.045
9	10.457	297	0.04	98	243316	6.26	1.06	0.050
10	10.968	1557	0.19	449	223034	5.76	1.06	0.055
11	11.308	606	0.07	190	281665	3.83	1.04	0.050
12	11.879	1926	0.23	519	236193	6.25	1.06	0.058
Sum		837965.0						

HPLC of **7h**

SAMPLE INFORMATION

Sample Name:	111-81-VAD-610		
Sample Type:	Unknown	Sample Set Name:	09022023_Serviss
Vial:	7	Acq. Method Set:	Serviss Gr5%_iz_UV
Injection #:	1	Processing Method:	Gr 5%
Injection Volume:	10.00 ul	Channel Name:	2998 Ch1 254nm@1.2nm
Run Time:	25.0 Minutes	Proc. Chnl. Descr.:	2998 Ch1 254nm@1.2nm
Date Acquired:	2/13/2023 2:27:41 PM EET	Acquired By:	System
Date Processed:	2/13/2023 3:47:09 PM EET		

Adamas C18-2 (4,6x150 mm)

15 min Gr. 5 - 95% ACN + 0.1% H3PO4; 5 min Iz. 95% ACN; 3 min Gr. 95-5% ACN; 2 min Iz. 5% ACN; F = 1mL/min; 40oC

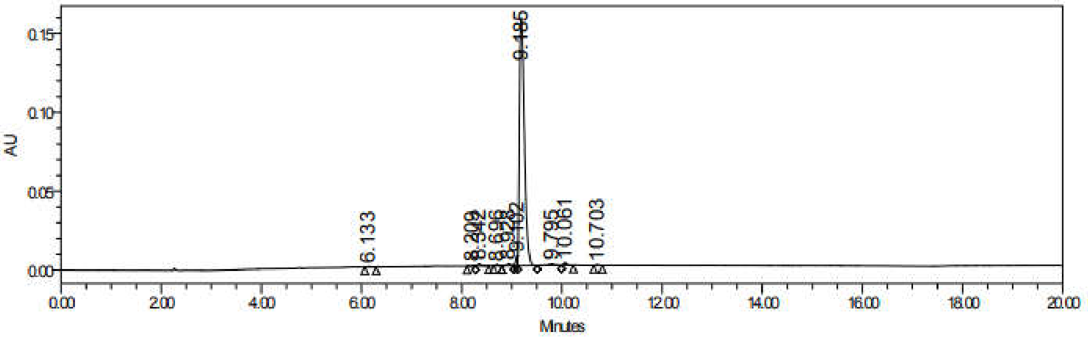

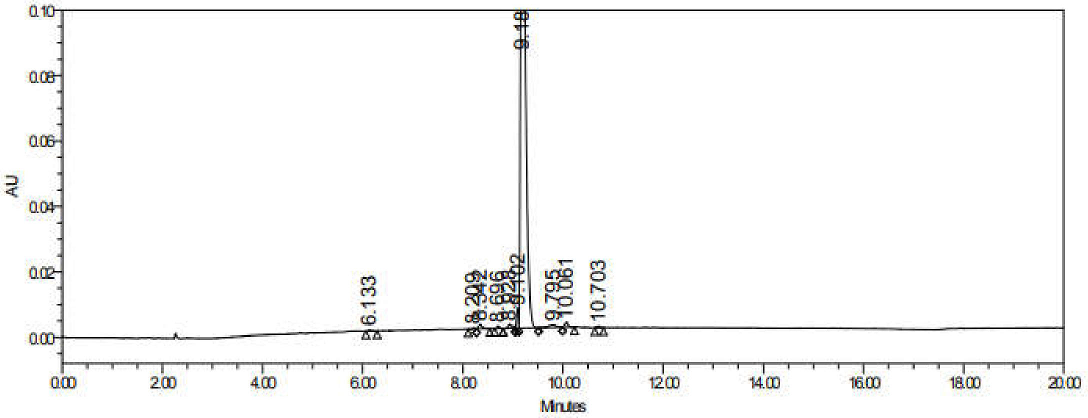

C=0.45 mg/ml (10%ACN/0.1%H3PO4)

	RT	Area	%Area	Height	EP Plate Count	Width@50%	Resolution	Selectivity
1	6.133	1695	0.15	317	36551	0.0755		
2	8.209	1595	0.14	413	122982	0.0551	18.762	1.443
3	8.342	6850	0.59	1307	83057	0.0681	1.275	1.020
4	8.696	2702	0.23	708	123944	0.0581	3.308	1.051
5	8.928	6964	0.60	1120	94381	0.0684	2.156	1.032
6	9.102	14241	1.23	6036				1.023
7	9.185	1106586	95.40	156667	37597	0.1115		1.011
8	9.795	10352	0.89	821	21233	0.1582	2.667	1.079
9	10.061	6787	0.59	1453	143525	0.0625	1.422	1.032
10	10.703	2223	0.19	447	123172	0.0718	5.638	1.075
Sum		1159995.5						

HPLC of **7i**

SAMPLE INFORMATION

Sample Name:	116-86-VAD-245	Acquired By:	System
Sample Type:	Unknown	Sample Set Name:	140223_serviss
Vial:	29	Acq. Method Set:	Serviss_Gr40%
Injection #:	1	Processing Method	Gr_40%
Injection Volume:	10.00 ul	Channel Name:	2998 Ch1 254nm@4.8nm
Run Time:	25.0 Minutes	Proc. Chnl. Descr.:	2998 Ch1 254nm@4.8nm
Date Acquired:	14.02.2023 1:05:42 PM EET		
Date Processed:	14.02.2023 2:15:09 PM EET		

Apollo C18-13 5um (4.6x150 mm)
15 min Gr. 40-95%ACN + 0.1%H3PO4; 5 min Iz. 95%ACN; 2 min Gr. 95-40% ACN; 3 min Iz. 40% ACN.
F=1mL/min. T=40oC.

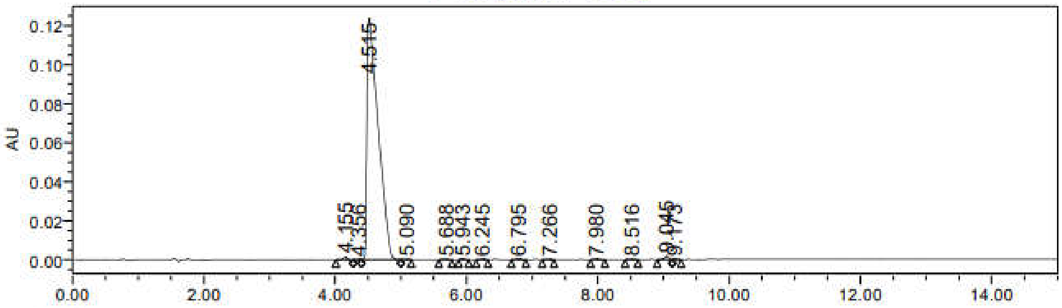

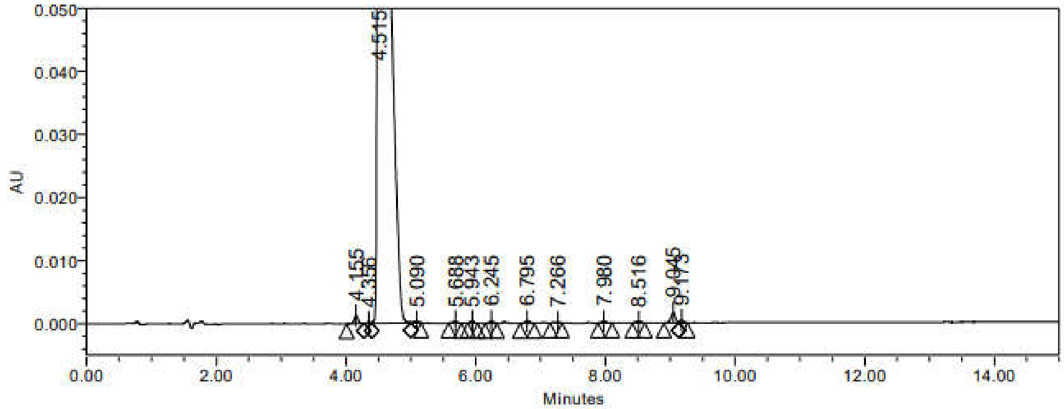

C=0,4mg/ml (50%ACN+0,1%H3PO4)

	RT	Area	% Area	Height	EP Plate Count	Resolution	Selectivity	Width @ 50%
1	4.155	5236	0.35	1312	25187			0.062
2	4.356	736	0.05	171			1.08	
3	4.515	1460658	98.22	123568	3093		1.06	0.191
4	5.090	1373	0.09	226			1.19	
5	5.688	1624	0.11	296	23190		1.17	0.088
6	5.943	1128	0.08	297	52157	2.02	1.06	0.061
7	6.245	1586	0.11	356	46239	2.75	1.07	0.068
8	6.795	1801	0.12	366	42970	4.46	1.12	0.077
9	7.266	871	0.06	160	30545	3.17	1.09	0.098
10	7.980	1377	0.09	308	69741	4.99	1.13	0.071
11	8.516	1331	0.09	235	46657	3.86	1.08	0.093
12	9.045	7774	0.52	1646	88375	3.80	1.08	0.072
13	9.173	1665	0.11	395			1.02	
Sum		1487158.9						

HPLC of **7j**

SAMPLE INFORMATION

Sample Name:	114-84-VAD-259	Acquired By:	System
Sample Type:	Unknown	Sample Set Name:	140223_serviss
Vial:	27	Acq. Method Set:	Serviss_Gr40%
Injection #:	1	Processing Method	Gr_40%
Injection Volume:	10.00 ul	Channel Name:	2998 Ch1 254nm@4.8nm
Run Time:	25.0 Minutes	Proc. Chnl. Descr.:	2998 Ch1 254nm@4.8nm
Date Acquired:	14.02.2023 11:20:55 AM EET		
Date Processed:	14.02.2023 1:08:31 PM EET		

Apollo C18-13 5um (4.6x150 mm)
15 min Gr. 40-95%ACN + 0.1%H3PO4; 5 min Iz. 95%ACN; 2 min Gr. 95-40% ACN; 3 min Iz. 40% ACN
F=1mL/min. T=40oC.

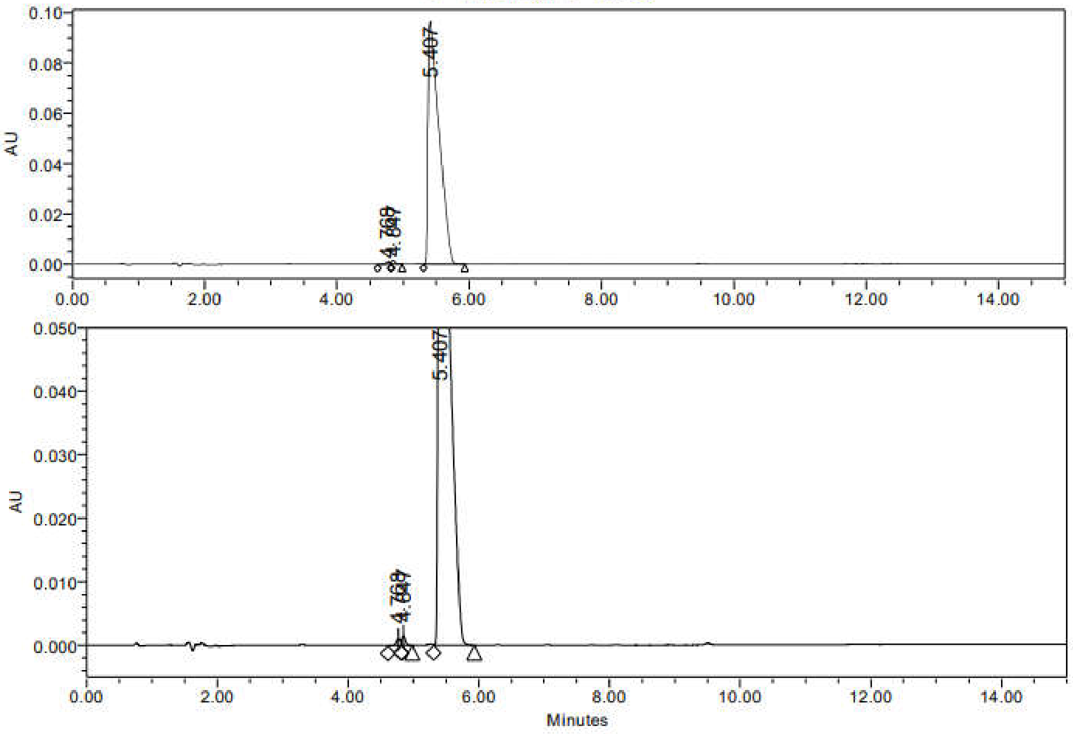

C=0,35mg/ml (50%ACN+0,1%H3PO4)

	RT	Area	% Area	Height	EP Plate Count	Selectivity	Width @ 50%
1	4.768	5187	0.46	1051			
2	4.847	5759	0.51	1537		1.02	
3	5.407	1107586	99.02	96307	4686	1.17	0.186
Sum		1118531.7					

HPLC of **7k**

Therapeutic efficacy of inhibitor 7f against *P. falciparum in vivo*

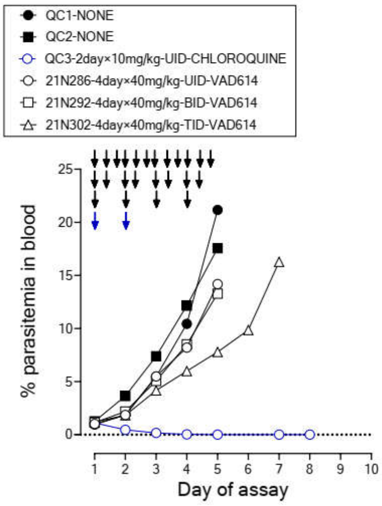

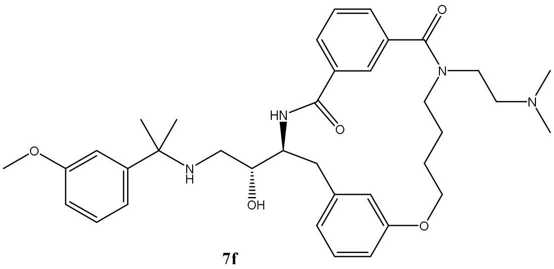

7f

Figure S1. Therapeutic efficacy of inhibitor **7f** against *P. falciparum in vivo*. The figure shows the parasitemia in peripheral blood of PfalcHuMice: untreated (QC1 and QC2), treated with chloroquine (QC3) or treated with inhibitor **7f** at 40 mg/kg, p.o., UID, BID or TID, (individuals 21N286, 21N292, and 21N302, respectively). The arrows indicate the time points of drug administration.
